# International perspectives on measuring national digital public health system maturity through a multidisciplinary Delphi study

**DOI:** 10.1038/s41746-024-01078-9

**Published:** 2024-04-12

**Authors:** Laura Maaß, Hajo Zeeb, Heinz Rothgang

**Affiliations:** 1 University of Bremen, SOCIUM Research Center on Inequality and Social Policy, Department Health, Long-Term Care and Pensions, Bremen, Germany; 2 Leibniz ScienceCampus Digital Public Health Bremen, Bremen, Germany; 3 Leibniz Institute for Prevention Research and Epidemiology—BIPS, Department Prevention and Evaluation, Bremen, Germany

**Keywords:** Health policy, Public health

## Abstract

Unlocking the full potential of digital public health (DiPH) systems requires a comprehensive tool to assess their maturity. While the World Health Organization and the International Telecommunication Union released a toolkit in 2012 covering various aspects of digitalizing national healthcare systems, a holistic maturity assessment tool has been lacking ever since. To bridge this gap, we conducted a pioneering Delphi study, to which 54 experts from diverse continents and academic fields actively contributed to at least one of three rounds. 54 experts participated in developing and rating multidisciplinary quality indicators to measure the maturity of national digital public health systems. Participants established consensus on these indicators with a threshold of 70% agreement on indicator importance. Eventually, 96 indicators were identified and agreed upon by experts. Notably, 48% of these indicators were found to align with existing validated tools, highlighting their relevance and reliability. However, further investigation is required to assess the suitability and applicability of all the suggestions put forward by our participants. Nevertheless, this Delphi study is an essential initial stride toward a comprehensive measurement tool for DiPH system maturity. By working towards a standardized assessment of DiPH system maturity, we aim to empower decision-makers to make informed choices, optimize resource allocation, and drive innovation in healthcare delivery. The results of this study mark a significant milestone in advancing DiPH on a global scale.

## Introduction

The ongoing digital transformation is reshaping various aspects of society, including healthcare systems^1^. Many countries have transitioned from paper-based to digitalized healthcare systems in recent years, bringing numerous benefits such as improved efficiency, cost reduction, real-time monitoring of health outcomes, and enhanced communication among stakeholders (e.g., physicians and patients)^1–6^. However, digitalization also raises data security and privacy concerns, necessitating robust protection measures and regulations^5^. Countries must provide the required infrastructure and data security framework to enable digital technologies in healthcare while protecting their population’s most sensitive data^7^. Additionally, user adoption, sustainability, and ethical design are crucial considerations for successfully implementing digital interventions^2,8,9^.

The digitalization of healthcare systems presents both opportunities and challenges, highlighting the complexity of this transformation. However, with limited resources, the degree of digitalization varies across national healthcare systems^10^. Assessing the maturity of a country’s digital healthcare system through objective measurements allows for benchmarking, policy learning, and informed decision-making. It enables policymakers to identify areas that require improvement, prioritize funding for interventions, education, and technical infrastructure, and track the progress of digitalization efforts over time^2,11^.

The World Health Organization (WHO) and the International Telecommunication Union (ITU) emphasize a comprehensive strategy for assessing electronic health (eHealth) systems, encompassing leadership, governance, implementation, and funding strategies, information-communication-technology (ICT) infrastructure, legal regulations, and digital literacy skills of the workforce and the general population^12^. Assessing the maturity of a healthcare system as a result of this needs to be conducted holistically and include the above-given dimensions. This approach implies the use of various multidisciplinary indicators.

Over the years, numerous indices have been developed to assess different aspects of digital health systems^11,13–25^. However, none of these indices have taken a comprehensive approach to measuring the maturity of digital public health (DiPH) systems as a whole. Instead, they have focused on specific areas such as national ICT infrastructure^12,14,26,27^, legal regulations, political support^1,11,12,27^, social acceptance^28^, or implementing interventions within healthcare systems^11,12,27^. Notably, there is a lack of indices that specifically address DiPH.

To comprehensively evaluate the maturity of DiPH systems, a holistic approach is essential beyond assessing eHealth systems alone, as the WHO & ITU proposed^12^. DiPH encompasses health promotion, disease prevention, and population health surveillance^29^, expanding eHealth’s focus on digitalizing healthcare^30^. Consequently, DiPH tools are upscaled interventions targeting groups or entire populations to enhance user health. They encompass eHealth tools alongside additional services, tools, or devices for health promotion and primary prevention^31^ (see the definitions in Box 1).

To address the lack of a comprehensive and holistic approach, our study aimed to establish international consensus on quality indicators for assessing the maturity of national DiPH systems. Drawing on existing validated indices and WHO recommendations^12^, we identified four areas to collect and categorize maturity indicators:

1. ICT: Examines the necessary ICT infrastructure requirements for integrating DiPH tools into routine care and health promotion programs nationally.
2. Legal: Focuses on political support, legal regulations, and data protection measures for the nationwide implementation and use of DiPH tools.
3. Social: Assesses the general public’s collective willingness and capacity to effectively utilize DiPH tools in routine care and health promotion efforts.
4. Application: Explores the adoption and utilization of DiPH tools within the national healthcare system by government entities or public institutions such as compulsory health insurance.

Our study aims to accomplish three primary objectives. Firstly, we sought to establish consensus on which interventions, technologies, and tools (referred to as DiPH tools) align with the definition of (digital) public health and should be included in the assessment (refer to Textbox 1). Secondly, we aimed to reach a consensus on quality indicators that effectively measure the maturity of DiPH systems in a manner adaptable to diverse national contexts. Finally, we examined how the proposed indicators fit with existing assessment tools used to analyze the maturity of individual aspects of our research topic^11,13–25^.

To address our study goals, we employed a Delphi study, a well-established method for achieving expert consensus on multidisciplinary and complex topics^32–35^. This technique is frequently applied for research in social science^36^ for topics with limited knowledge or uncertainty^37,38^. Compared to focus group discussions, the Delphi technique allows every participant to express their opinions equally without risking individual experts dominating the debate and with the protection of anonymity to express their thoughts freely, reducing the risk of social desirability bias^36^. Additionally, as participants receive feedback on the overall voting behavior after each round, they can change their perspective straightforwardly^39–41^. We are, therefore, confident that the Delphi methodology fits our research goals due to the uncertainties in holistic DiPH-system maturity assessment, pragmatic reasons (as experts can participate cost-efficiently from all around the globe), and its ability to obtain consensus among participants on multidisciplinary and complex topics.”

Box 1 Definition of Public Health and Digital Public Health Public Health is “the science and art of preventing disease, prolonging life and promoting, protecting and improving health through the organized efforts of society”^29^

Digital Public Health is “not a discipline per se, but an asset [the public health] community has to [fulfill] its aims and mission. The health system goals of quality, accessibility, efficiency, and equity of healthcare, embraced by public health professionals, are unaltered by the process of digitalization”^77^

## Results

**Fig. 1:**
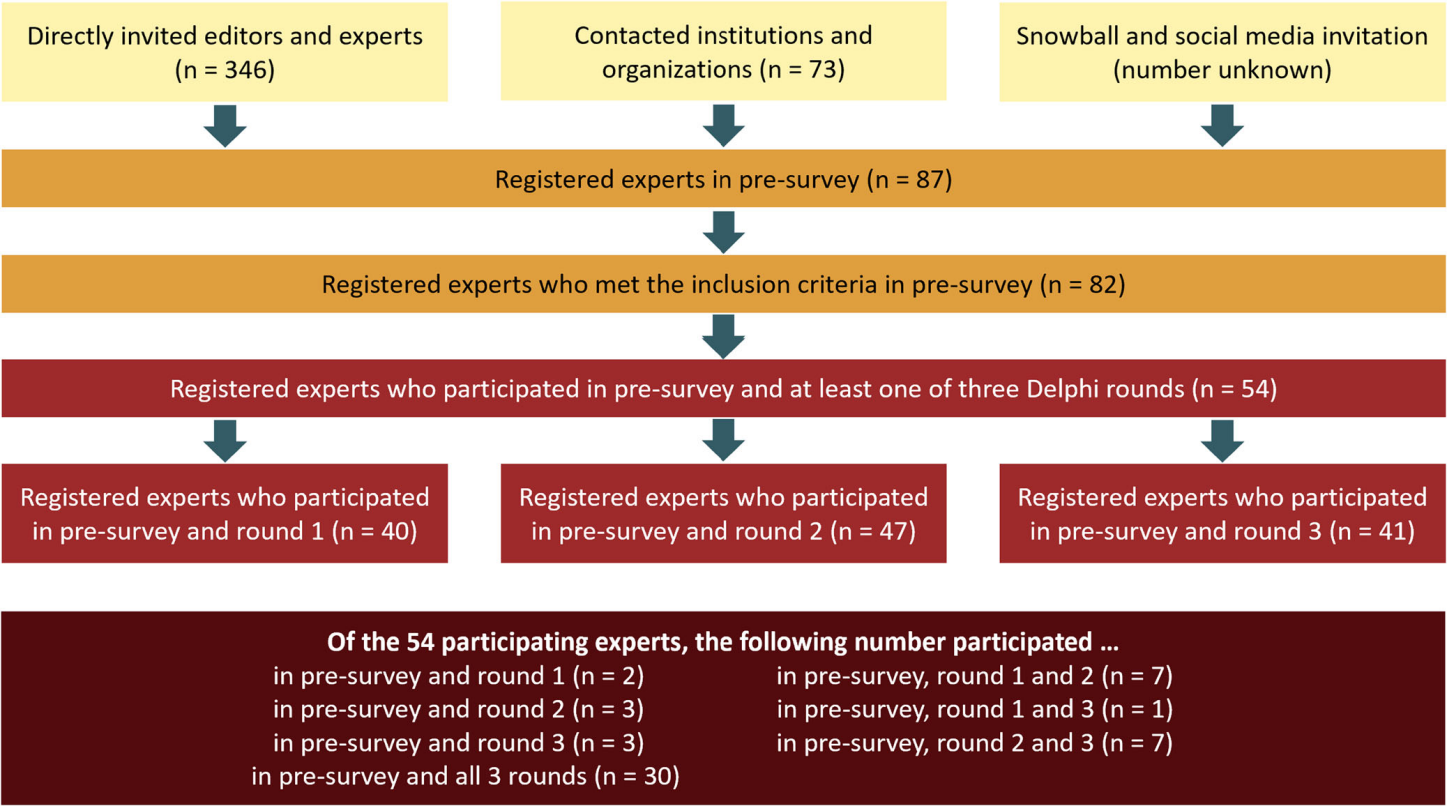
Recruitment flow and final study cohort size. In total, 87 experts registered for the Delphi study, of which 82 met the inclusion criteria. Of these, 54 participated in at least one of the three Delphi panels with 40 to 47 experts per survey round.

During the pre-survey, 87 experts expressed their written interest in contributing to our Delphi study, of which 82 met the inclusion criteria. Eventually, 54 specialists actively participated in at least one of the three official Delphi rounds study by providing and ranking quality indicators. The recruitment flow and study cohort size per round are displayed in Fig. 1. Of the 54 experts, 40 participated in the first round (74%), 47 in the second round (87%), and 41 contributed to the third and final round (76%).

The characteristics and demographics of the 82 experts who met our inclusion criteria and those who participated in the official three Delphi rounds are displayed in Table 1. Amongst the experts with the professional background summarized as “Other” are experts (one each) from health economics, ICT, nursing science, pharmacy, policy analytics, politics, science laboratory technology, and urban planning, displaying a variety of different fields contributing to the study. From a geographical perspective, most experts came from Germany (27 registered, 16 participated in the first round, 17 in the second, and 14 in the third round). They were followed by six registered experts from Portugal of which 4 participated in each Delphi panel (accompanied by experts from Belgium, Croatia, Finland, France, Greece, Italy, Malta, the Netherlands, Norway, Sweden, Switzerland, and the United Kingdom). For Africa and South America, one expert each from Ethiopia, Nigeria, Uganda, Brazil, and Ecuador registered, however only the experts from Ethiopia and Brazil contributed to the study during rounds two and three. For the Australasian region, three experts from Australia registered, accompanied by experts from Sri Lanka, Turkey, the United Arab Emirates, Kazakhstan, and the Philippines. For North America, three Canadians, and one expert from the United States of America registered.

**Table 1:** Panel characteristics

Characteristics	Registered experts (*n* = 82)	Experts in first round (*n* = 40)	Experts in second round (*n* = 47)	Experts in third round (*n* = 41)
Highest Qualification				
Bachelor’s Degree	4 (4.9%)	—	—	—
Master’s Degree	20 (24.7%)	11 (27.5%)	14 (29.8%)	11 (26.8%)
Diploma	4 (4.9%)	2 (5%)	2 (4.3%)	2 (4.8%)
Medical Doctor	4 (4.9%)	3 (7.5%)	3 (6.4%)	4 (10%)
PhD	31 (37.8%)	18 (45%)	19 (40.4%)	16 (40%)
Professor	19 (23.5%)	8 (20%)	9 (19.1%)	7 (17.5%)
Professional Background				
Computer Science	2 (2.4%)	1 (2.5%)	1 (2.1%)	1 (2.4%)
Epidemiology	3 (3.7%)	1 (2.5%)	1 (2.1%)	1 (2.4%)
Ethics	2 (2.4%)	2 (5%)	2 (4.3%)	2 (4.8%)
Law	5 (6.1%)	4 (10%)	4 (8.5%)	3 (7.3%)
Medical Informatics	8 (9.8%)	6 (15%)	6 (12.8%)	5 (12.2%)
Medicine	21 (25.6%)	6 (15%)	9 (19.1%)	7 (17.1%)
Public Health	28 (34.1%)	17 (42.5%)	18 (38.3%)	15 (36.6%)
Sociology	4 (4.9%)	—	1 (2.1%)	2 (4.8%)
Other (each initially less than 2)	9 (11.0%)	5 (12,5%)	5 (10,6%)	4 (9,8%)
Sector				
Academia/Science	53 (64.6%)	31 (77.5%)	33 (70.2%)	30 (73.2%)
Government	12 (14.6%)	4 (10%)	6 (12.8%)	4 (9.7%)
Clinical	5 (6.1%)	2 (5%)	2 (4.3%)	—
Non-Government-Organization	5 (6.1%)	1 (2.5%)	3 (6.4%)	2 (4.8%)
Private Company	4 (4.9%)	3 (7.5%)	3 (6.4%)	3 (7.3%)
Other (less initially than 2 each)	3 (%)	1 (2.5%)	—	1 (2.4%)
Years of experience in the professional field in general				
Min-Max	3–40	3–40	3–40	3–40
Average (SD)	15.5 (10.5)	14.4 (11.2)	14.9 (11.2)	15.6 (11.2)
Years of experience in digital health				
Min-Max	1–30	1–30	1–30	1–30
Average (SD)	6.9 (6.8)	6.3 (6.3)	6.1 (6.0)	6.4 (6.4)
Contributing experts by area (contribution to more than 1 area possible)				
ICT	37*	16	20	14
Legal	39*	14	22	16
Application and Tools	63*	23	31	26
Social	45*	15	19	16
Age				
Min-Max	27–91	27–91	27–91	27–91
Average (SD)	44.2 (12.0)	41-8 (10.3)	41.4 (11.2)	42.2 (11.2)
Gender				
Female	33 (40.2%)	14 (17.1%)	15 (18.3%)	14 (17.1%)
Male	49 (59.8%)	28 (34.1%)	32 (39%)	26 (31.7%)
Region				
Europe	61 (74.4%)	31 (77.5%)	37 (78.7%)	31 (75.6%)
Africa	3 (3.7%)	—	1 (2.1%)	1 (2.5%)
Australasia	11 (13.4%)	6 (15%)	5 (10.6%)	4 (10%)
North America	5 (6.1%)	4 (9.5%)	3 (6.4%)	3 (7.5%)
South America	2 (2.4%)	1 (2.4%)	1 (2.1%)	1 (2.5%)

*Areas of interest for which experts would contribute in rounds 1–3 as declared by experts in the pre-survey. Experts did not contribute to any area during the pre-survey.

**Fig. 2:**
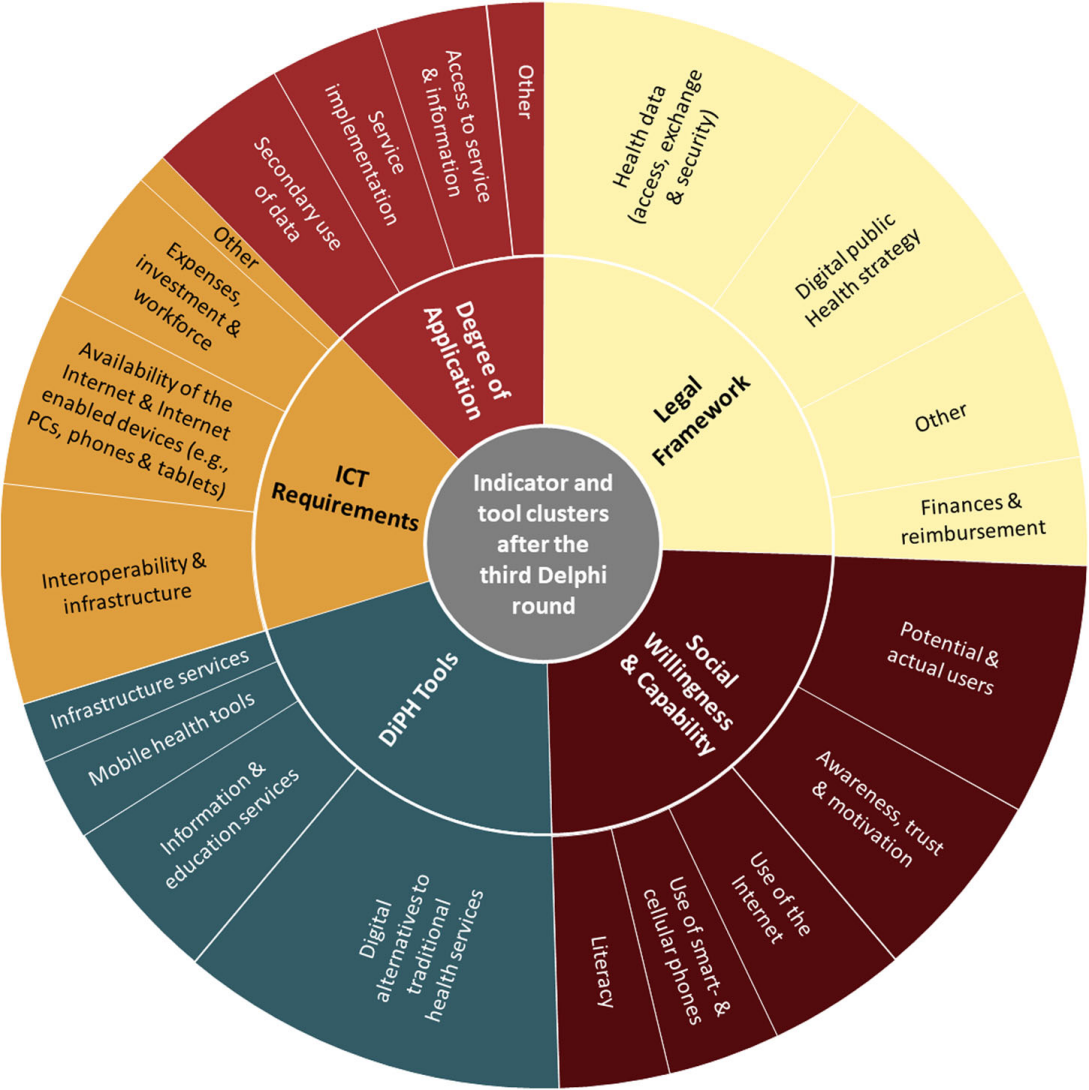
Clusters per sub-dimension after round 3, sorted by size. The finally agreed upon 96 indicators were clustered across 18 clusters. The 25 digital public health tools can be sorted into four clusters. An overview of all indicators proposed during the progress including their agreement rate is available in the Supplementary Information.

Our international and multidisciplinary Delphi study concluded with 96 indicators (21 for *ICT*, 31 for *Legal*, 29 for *Social*, and 15 for *Application*) and 25 *DiPH tools*. After the third round, the indicators and tools were grouped into 22 clusters among the four sub-domains (see Fig. 2). An overview of all indicators and tools, including participation rate and overall rating per indicator, is listed in Supplementary Information 1.

### Representation of proposed indicators among other indices

We assessed how many proposed indicators were already included in other assessment tools and indicator lists. Overall, 48% of the indicators were covered by at least one published and validated index or indicator list^11,13–25^. However, our analysis has shown that particularly those indicators related to general ICT infrastructure, cybersecurity, and the regulation and use of health data were already sufficiently covered among existing indices. Here, we observed covering rates of 57% (for *ICT*) and 55% (for *Legal*). Conversely, we identified a lack of validated indicators specifically targeting DiPH-service implementation (40% inclusion) and measuring population interest and capability nationally (only 38% coverage).

### Indicators on information-communication-technology requirements

ICT-system maturity is crucial for the success of a national DiPH service rollout. Without a sufficient broadband network in the country (especially in rural areas), potential users might be unable to access the system. This digital divide could potentially increase health inequalities^42,43^ and is, therefore, essential when assessing the maturity degree of DiPH systems as a core requirement. Our participants also proposed multiple indicators to determine the general ICT-system maturity (like prices for and availability of broadband internet connections) and financial support to improve the sector. Additionally, the experts emphasized the need for indicators that bring together the healthcare and the ICT sectors through indicators on physician offices with Internet connections, training in DiPH, or the use of electronic documentation systems in hospitals. All ICT indicators with at least 70% importance agreement during R3 are displayed in Table 2.

**Table 2:** ICT indicators that reached the needed agreement in the third panel

ID	Indicator	Elsewhere included
	Indicators on expenses, costs, investment, and workforce	
I02	The average prices for fixed broadband internet connections per month	21
I07	The annual spending by the government on the information-communication-infrastructure	22
I09N	The level of training in the use and billing of digital public health services by healthcare professionals	—
I11	The number of skilled professionals in data science	21
I13N1	The average number of participants in digital public health tool training programs in a year by different target groups (e.g., health professionals or patients)	—
	Indicators on the availability of the Internet and Internet-enabled devices such as PCs, phones, and tablets	
I14	The share of households/hospitals/physician offices connected to the Internet (by bandwidth)	21
I17N	The share of specific locations that are equipped with at least one internet-connected device by location	21
I18	The number of internet-connected devices per 100 people by device (e.g., computers, smartphones, tablets, or notebooks)	—
I19	The percentage of the overall population with access to the Internet (by speed)	13, 16, 18, 21, 22, 24
I20	The percentage of the overall population with access to computers	21
I21	The percentage of the population covered by at least a 3 G mobile network	13, 16, 18, 20–22, 24
I23	The percentage of the overall population with access to smartphones	15, 18, 20, 21, 25
	Indicators on interoperability and infrastructure	
I24N	The percentage of all hospitals that use electronic documentation systems for patient care	—
I25	The availability of health information exchange platforms	11
I26N2	The existence of the possibility to access personal health data based on consent and ID approval	11, 17
I327	The degree of existing telematic infrastructure	—
I28	The degree of interoperability of health systems	11, 17
I29	The degree of compliance of specific interventions with the data exchange and interoperability standards	—
I30	The existence of interoperable data end-to-end encryption	—
I31	The number of integrated digital systems in the healthcare system	—
	Indicators on other topics	
I33N	The number of technical malfunctions per digital public health tool reported per year	—

### Indicators on the legal regulation and political support

Several validated assessment tools for digital health regulation exist^11,15,17^. However, currently none for DiPH. Nevertheless, as digital health (focusing on personalized healthcare) is a sub-dimension of the more holistic DiPH^44^, we argue that the validated digital health regulation indicators might be applicable to some aspects of the developed DiPH indicators. Unsurprisingly, most indicators proposed through the panel assess health data’s access, exchange, and security, which were also of interest for various validated indices (see Table 3). The global increase in the creation and use of health-related data holds promise for evidence-based and data-driven DiPH programs. However, poor data protection regulations pose a risk to individual users of data breaches or misuse. Therefore, countries must implement strong data governance structures and offer political support to protect these sensitive data from being misused^45^. This importance is mirrored in the expert agreement for the need for a legal framework in DiPH data exchange and regulations for accessing health data through EHRs (both received 100% agreement).

**Table 3:** Legal indicators that reached the needed agreement in the third panel

ID	Indicator	Elsewhere included
	Indicators on health data (access, exchange, security)	
L01	The percentage of user consent to health data	—
L02	The coverage of international standards in stored data	11, 17
L03	The existence of a legal framework for exchanging health data digitally between different stakeholders (e.g., between healthcare providers or researchers)	11, 17
L04	The degree of political support in data transfer & exchange	11
L05	The existence of a legal framework for the secondary use of health data	11
L06	The existence of regulations for access to health data through electronic health records (for patients, care providers, and researchers)	11
L07	The existence of legislation regulating the interaction between the digital data of a patient’s health and the data of bioinformatics / genetic information of the biomaterials of this patient	15
L08	The level of encryption of personal and health data	—
L11	The coverage of metadata labels in sensitive data	19
L12	The number of critical failures in security points	—
L13	The number of weaknesses in security perimeters	—
L14	The level of data security	11, 17, 19, 22
	Indicators on the digital public health strategy	
L19	The existence of a political strategy to digitalize the healthcare system	11, 17
L20	The existence of a digital public health strategy within the governmental health strategy	11, 17
L21	The existence of guidelines for planning & implementing digital public health tools	11
L23	The existence of legal supervision of the implementation of national digital public health programs	11
L24	The existence of a department for digital health in the Health Ministry	17
L25	The existence of regulation on the function of e-health products	—
L26	The existence of a legal right for citizens to be provided with digital health services	—
L27	The existence of a digital public health policy engaged with the protection of fundamental rights of vulnerable groups (e.g., children, adolescents, mentally disabled people)	—
L28	The existence of a policy to promote innovation and the development of digital tools in the public health system	—
	Indicators on finances and reimbursement	
L29	The existence of a public funding scheme for digital public health interventions on a regional and national level	11
L30	The existence of investment and reimbursement possibilities from the government	11
L31	The annual spending by the government as support for the implementation of digital technologies in healthcare	17
L33	The existence of financial incentives for health professionals to participate in offering digital public health services	11
	Indicators on other topics	
L17	The existence of a policy or legislative reform to allow access to digital assets	—
L41	The existence of legal liability for public health managers for digital health contracts that harm patients or the public interest	—
L42	The existence of policy standards for transparency and the protection of fundamental rights in using artificial intelligence in digital public health	—
L43	The level of transparency and accessibility of digital health contracts to public oversight and law enforcement agencies for anti-corruption purposes	—
L44	The effectiveness of informed patient consent for using personal health data in adult patients	—
L45	The existence of unique procedures to protect children, adolescents and mentally ill patients who are unable to give their consent	—

### Indicators on the social willingness and capability to use DiPH tools

The sub-dimension *Social willingness and capability* to use DiPH tools in healthcare and health promotion had the lowest share of indicators covered among already existing validated assessment tools (only 38%). Adding to this observation, no proposed indicator received over 94% agreement, pointing towards a potentially lower consensus of experts in this field than ICT requirements or needed legal regulations and potentially bigger research gaps. The proposed indicators are displayed in Table 4. Most of the indicators focused on the users of DiPH tools. Still, the panel also deemed more general indicators on digital and health literacy and the use of mobile devices and the Internet as crucial for assessing the capability of a population to use DiPH tools.

**Table 4:** Social indicators that reached the needed agreement in the third panel

ID	Indicator	Elsewhere included
	Indicators on potential and actual users	
S01	The number of people that are willing to use a digital public health tool or participate in a digital public health intervention	11, 17
S02	The number of potential users of the same digital public health tool	—
S03	The number of potential users of a digital public health tool who have adequate access to the web	—
S04	The share of the eligible population who have used at least one digital public health intervention for routine care and health promotion in the previous year	—
S06	The number of patients using apps to interact with local services	—
S08	The adherence of users to the intervention (in percentage)	—
S09	The ratio of electronic health records to the total number of user records	15
S11N2	The share of the population that uses any health or medical app by reason (e.g., health promotion, wellness, tracing)	11
S12	The number of digital public health professionals	—
	Indicators on awareness, trust, and motivation	
S17	The perceived usefulness of specific digital public health tools by groups on a Likert scale	—
S18	The share of the population that trusts digital health services	—
S19	The motivation to access electronic health services by groups of people	—
S20	The awareness of health professionals about the value of data and the possibility of their use by information-communication-technology	—
S21	The awareness of groups of people that the intervention exists	—
S23N1	The self-reported satisfaction rate with digital public health intervention by user group	—
S23N2	The share of the population that is more satisfied with using the digital public health intervention compared to standard care	—
	Indicators on literacy	
S24	The average level of digital literacy by different target groups on a Likert Scale	16, 20, 22
S25	The average level of digital health literacy by different target groups on a Likert Scale	—
S26	The average level of health literacy by different target groups on a Likert Scale	—
S27	The average level of information-communication-technology skills by groups of people	14, 16, 18, 21
	Indicators on use of smart- and cellular phones	
S28	The share of the population that uses a smartphone by different target groups	21, 25
S29	The share of the population that owns a smartphone by different target groups	21, 25
S30	The smartphone penetration rate versus the use of mobile devices for routine care and health promotion in the population	—
S32	The proportion of healthcare users using smartphones and other digital devices	—
	Indicators on use of the Internet	
S33	The active mobile-broadband subscriptions per 100 inhabitants	13–16, 22
S34	The fixed broadband internet subscriptions per 100 inhabitants	18, 20, 25
S35	The percentage of Internet users in the country	14–16, 18, 20–23, 25
S36	The share of the population that uses the Internet for gathering health information by different target groups	16
S37	The number of searches for specific digital public health interventions measured on Google Trends	—

### Indicators on the application degree of digital public health tools

Although various validated indices cover the aspects of the service’s implementation degree or secondary use of health data^11,15,17^ our participants consented that additional indicators are needed, especially in evaluating the implementation and access to the DiPH service. Table 5 displays the final distribution among the four clusters. All indicators focusing on the *secondary use of health data* were included in already developed assessment tools measuring digital health service maturity. Nevertheless, one needs to remember that digital health maturity tools might differ in their requirements compared to DiPH maturity assessment models.

**Table 5:** Application indicators that reached the needed agreement in the third panel

ID	Indicator	Elsewhere included
	Indicators on access to the service and information	
A02N2	The availability of reliable information on specific digital public health services	—
A04N	The percentage of the overall population with access to the digital public health tool	—
A05	The availability of reliable health information in a digital format	—
A06	The proportion of persons who cannot access their digital data and the provision of alternative access	—
	Indicators on secondary use of data	
A07N1	The share of patient health data used for evaluating healthcare services	11
A07N2	The share of population health data used for public health monitoring	11, 15
A09N	The degree of technical/syntactic/semantic interoperability	15
A10N	The availability of a unique identifier to link health data for a person between different digital public health tools/platforms	11
A11	The degree of data accessibility	11, 17
	Indicators on service implementation	
A12	The degree to which an intervention is established (e.g., local pilot, communal, regional, national)	11
A13N	The extent of redundancy in workflows created by introducing digital public health tools/interventions to complement existing workflows	—
A16	The proportion of digital public health interventions considering health equity in their planning, implementation, and evaluation	—
A18	The number of regulated digital health services included in routine care	—
	Indicators on other topics	
A35	The degree of change of specific health indicators as an outcome measure of the impact of digital tools	—
A40	The average rating of a digital health service in a relevant rating portal	—

### Digital public health tools

**Fig. 3:**
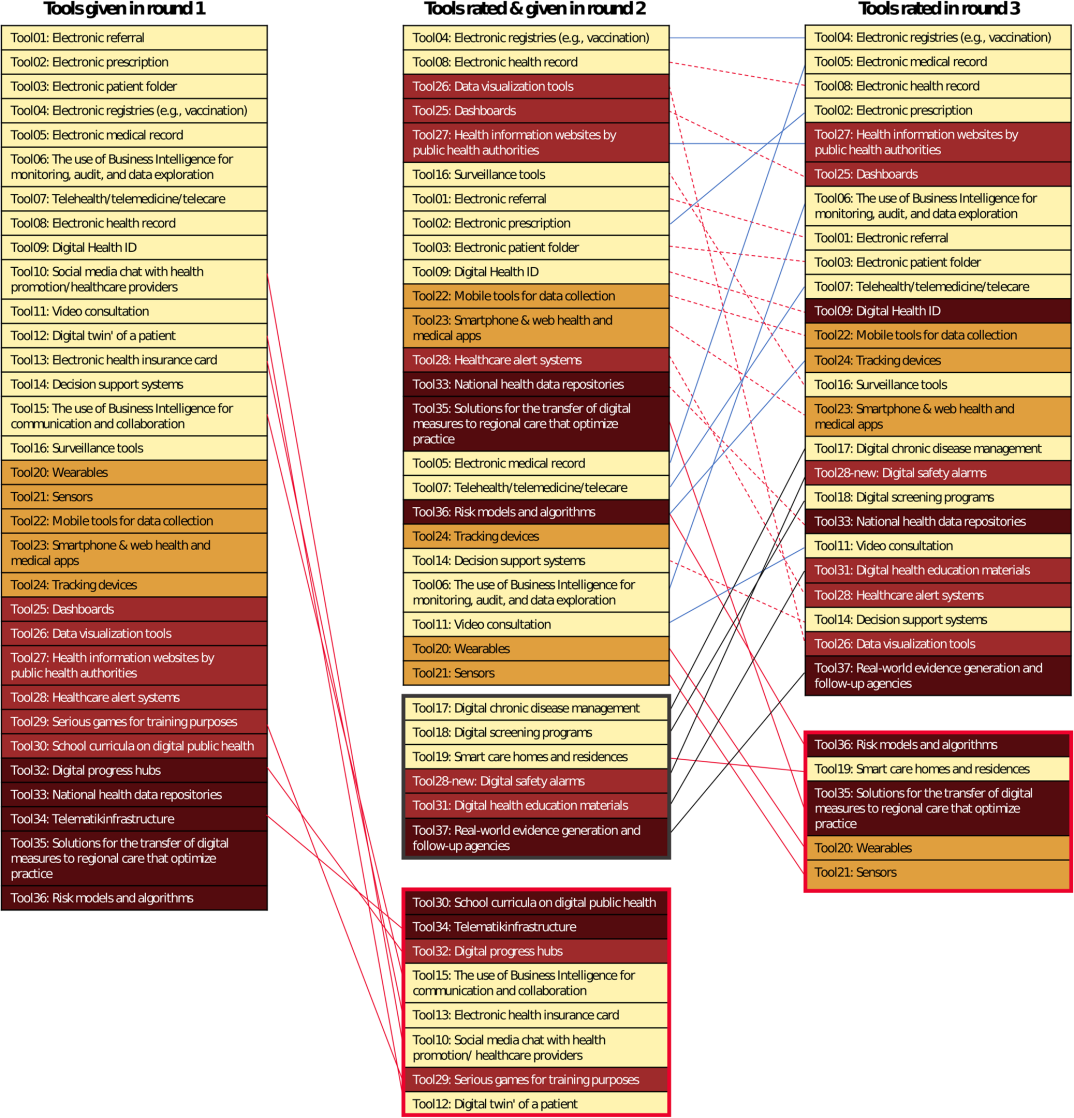
Change in ranking for DiPH tools according to expert agreement. The tools are highlighted according to their cluster. Yellow Digital alternatives to traditional public health tools, Orange Mobile health tools, Red Information or education tools, Brown Infrastructure tools. In round 1, tools were only proposed and the first rating happened in round 2. Here, all tools with less than 70% agreement were excluded (red lines leading to red box; same for round 2 to round 3). Newly added tools, first mentioned in round 2, are displayed in the black box below the original tools from round 1. As these tools were only rated once (from round 2 to 3), the connecting line from round 2 to round 3 is black for all tools with at least 70% agreement. Whereas tools which were rated in round 2 already but received a lower rating in the third round (with still at least 70%) are connected through a red dashed line. On the other hand, tools with increased agreement in round 3 compared to round 2 are connected through a blue line.

25 *DiPH tools* were named and agreed upon as DiPH tools. During R3, wearables and sensors received the lowest rating (65% each), whereas electronic registries (e.g., for vaccination) scored highest with 100% agreement on suitability. Figure 3 summarizes the agreement change for somewhat and very suitable between all three rounds. A blue line displays an increase in agreement. In contrast, a dashed red line shows a decline in agreement (however, the tool still received at least 70% agreement), and a thick red line explains which tools received below 70% agreement and were, therefore, excluded. Tools in the black box in the middle column were introduced during the second panel (R2) and only ranked in the final round (R3). Although the position change among the columns might appear drastically, the average share of agreement among those tools with at least 70% agreement did not change severely between R2 (87%) and R3 (84%).

## Discussion

This Delphi study aimed to comprehensively assess the maturity of national DiPH systems by collecting 96 indicators aligned with WHO and ITU recommendations^12^. Interestingly, only a minority (48%) of these indicators were already covered by existing validated indices. This discrepancy may be due to our focus on DiPH maturity, which encompasses population-oriented services for health promotion, surveillance, monitoring, and research^44,46,47^, in contrast to the currently more dominantly recognized and evaluated perspective of digital health that primarily emphasizes individual patient healthcare, treatment, and personalized medicine^48^. Consequently, the assessment requirements might differ, impacting the implementation of specific tools or services, required ICT infrastructure, legal regulations, and societal willingness and capacity to utilize such tools.

While the Delphi process sought consensus among experts from diverse regions to develop indicators applicable in multiple settings and healthcare systems, cultural and geographical differences likely influenced the consensus-building process. Our subgroup analysis (see Supplementary Information 2) revealed varying votes among participants from different regions, particularly concerning *DiPH tools*. For instance, during R3, experts from Germany advocated towards excluding business intelligence and intelligent care homes as DiPH tools, while experts from other regions supported their inclusion. Conversely, German experts voted to include risk models, solutions for transferring digital measures to regional care, data visualization tools, and wearables as DiPH tools, which were deemed unsuitable by other experts.

These discrepancies may arise from divergent interpretations of public health itself. In Germany, public health is deeply rooted in social medicine, prioritizing health promotion and primary prevention. The definition by Winslow in 1920 characterizes public health as “the science and art of preventing disease, prolonging life and promoting physical health and efficiency through organized community efforts […]”^49^. Consequently, German public health experts are more inclined to view interventions as DiPH tools if they target primary prevention, health promotion, or population health surveillance goals (e.g., wearables, risk models, or data visualization tools)^50^.

However, public health perspectives in other countries may differ. Alternatively, one could argue that health and public health interventions are a public good^51,52^. As long as digital interventions for health purposes are accessible to the user group without charge (e.g., covered by the state or compulsory health insurance), they could be considered DiPH tools. This broader approach could encompass system services like telemedicine but exclude wearables, as users typically bear the cost of smartphones or smartwatches (as supported by the multinational experts’ general agreement in the Delphi study). These varying ratings are closely associated with differing understandings of public health and DiPH and need to be reconsidered when developing assessment tools for multinational settings.

One of the key strengths of our study lies in its robust and inclusive design. We implemented a comprehensive, multi-pronged, and international recruitment strategy, contacting experts through various channels. This approach yielded in 82 participating experts from 27 countries across six continents, representing a diverse range of scientific fields. This range of expertise, combined with the Delphi method for consensus-building, enabled us to develop a comprehensive set of indicators of internationally agreed importance that can be used to measure the maturity of DiPH systems in different settings and healthcare systems.

The decrease in agreement regarding the importance of vaguely worded indicators demonstrates our study’s methodological effectiveness. This decline indicates that participants understood and acted upon our feedback after each round. Including probes in ranking such indicators further facilitated the convergence and precision necessary for indicator formulation and inclusion in our study. Last, the achieved response rates reinforce the strength and suitability of our chosen methodology. We observed strong interest and commitment from the participating experts among those who participated at least once in our study (54 of the registered 82 experts contributed to at least one round, and 30 experts participated in all three surveys). This level of engagement highlights the significance of our research and demonstrates the experts’ dedication to shaping the future of DiPH systems. Nevertheless, although 66% of all registered experts participated in at least one round (54/82), shedding light on their sound commitment, attrition during the process can also be considered a limitation. We did not ask for indicators during the pre-survey so that vital contributions may have been lost. However, we can only speculate on the motives why experts did not take part in subsequent rounds since we did not receive any additional comments from them.

In terms of limitations, it should be noted that we cannot guarantee that all participants used the “I cannot rate this indicator due to lacking expertise” option accurately when assessing the indicators and DiPH tools. However, the overall rating for poorly worded indicators decreased over the study period. Further, it is essential to acknowledge that the 70% threshold criterion for consensus on indicator importance was chosen arbitrarily despite incorporating best practice guidelines and reviewing other Delphi approaches for guidance^2,53,54^. Another potential limitation is the presence of language bias since the study was conducted in English, requiring participants to submit indicators in English. This language requirement may have excluded experts without English proficiency and could have led to misunderstandings of developed indicators or our instructions. Nonetheless, considering the inclusion of experts from diverse continents, we remain confident that the identified indicators, with appropriate translation, have the potential to be accepted and applied in non-English-speaking settings.

Additionally, we did not ask registered experts how they became aware of our study. Therefore, it remains unclear how many participants registered after being invited by colleagues and friends (snowballing recruitment) after seeing the call for participation on social media or reading about our study in their organization’s or association’s newsletter.

We recognize that our study panel has an overrepresentation of experts from Europe, more precisely Germany, and those with a background in public health. However, through sub-group and sensitivity analysis (see Supplementary Information 3), we demonstrated that significant differences in R2 occurred only for comparing European experts and experts from other continents in the sub-domains *ICT* and *DiPH Tools*. As we did not observe any significant differences for the overrepresented group of public health or German experts during R2, the conflicts for the European sub-group seem to stem from the experts from other European countries. The differentiation in *DiPH Tools* might hint at a varying understanding of public health, as discussed earlier. Overall, the analysis supports our assumption that the selection of indicators during R2 was not influenced by an academic background in public health as represented in the study and, therefore, did not influence the indicators included at the beginning of R3.

We put substantial effort into recruiting experts from all continents, e.g., by repeatedly contacting major organizations and directly contacting published authors in the overall field. Including more expertise from these countries is clearly desirable, nevertheless, our results provide broad insights into digital maturity assessment that certainly carries meaning beyond the countries currently included.

Finally, we point out that there is a lack of standardized procedures regarding the inclusion of non-participating experts in later Delphi rounds. While some Delphi studies refrain from including experts in the continuous iteration process who have not contributed to previous Delphi studies^55,56^, we have invited all experts for each round who registered during the pre-survey regardless of their prior contribution. We decided for this approach not to reduce our cohort size further. While this method was in line with other Delphi studies^57,58^, it might have raised issues in the iteration process as previously non-participating experts might have influenced the overall voting result of the cohort. However, our balanced panel analysis (see Supplementary Information 3) displayed no statistically significant differences in overall voting among the 30 experts who contributed to all rounds and the general participants in R2 and R3. Therefore, we are confident that our approach did not negatively impact the iteration process of our Delphi study.

Future research needs to be conducted to investigate how data can be effectively collected for the proposed indicators and to assess their suitability in comprehensively evaluating the maturity of DiPH systems across diverse cultural and geographical contexts. Furthermore, conducting regression analysis using real-world data to explore potential correlations among the individual indicators is crucial. We can gain valuable insights contributing to evidence-based decision-making and assessments by exploring these relationships.

We are planning to use the proposed DiPH indicators together with validated digital health, ICT, regulation, and sociological indicators to form a measurement tool that will assess the national maturity of DiPH systems according to the WHO toolkit: The Digital Public Health Maturity Index (DIPHMI). Its potential to inform policy decisions and improve resource allocation will make it a valuable tool for policymakers and stakeholders.

Another area requiring further research is the ever-evolving nature of DiPH: While some established tools and services, such as telehealth and health apps, have been identified, the integration of emerging technologies like blockchain, big data analytics, and artificial intelligence remains unclear. Understanding their contributions and identifying the specific needs they may pose to DiPH systems necessitates ongoing exploration.

Lastly, we want to point out that our results are purely based on expert opinion. However, due to the complexity and interdisciplinarity of the topic, it is also crucial to understand the needs of practitioners and representatives from the general population (such as patient representatives). Participatory approaches and citizen science can lead to an increased research capacity, better knowledge, and benefits for citizens^59^. Adding lay and traditional knowledge to scientific data can lead to a more effective response to complex problems or topics, such as measuring the maturity of DiPH systems. Especially for the social component regarding the willingness and capability of practitioners and laypeople to use DiPH tools in their routine healthcare and health promotion, it will be crucial to include representatives from these groups. This is why we encourage future research on the topic for more inclusive approaches that also include representatives from groups other than scientific experts.

In conclusion, the collaborative efforts of our multidisciplinary and multinational Delphi panel have culminated in a remarkable list of 96 indicators to be considered when assessing a national DiPH system’s maturity. This study holds immense promise, as its findings will resonate with a wide range of stakeholders, including public health authorities, governments, researchers, and industry professionals. The relevance of our research extends far beyond academia, creating a ripple effect that will positively impact the international public health landscape. By fostering a global vision for a comprehensive evaluation of DiPH systems, our consensus study serves as a first step towards international policy learning, benchmarking, and an improved allocation of limited resources in DiPH systems worldwide. By embracing the insights gleaned from our study, policymakers, researchers, and practitioners will be empowered to strengthen their digital infrastructure, enhance collaboration, and ultimately improve the health and well-being of populations on a global scale.

## Methods

### Structure of the Delphi study

**Fig. 4:**
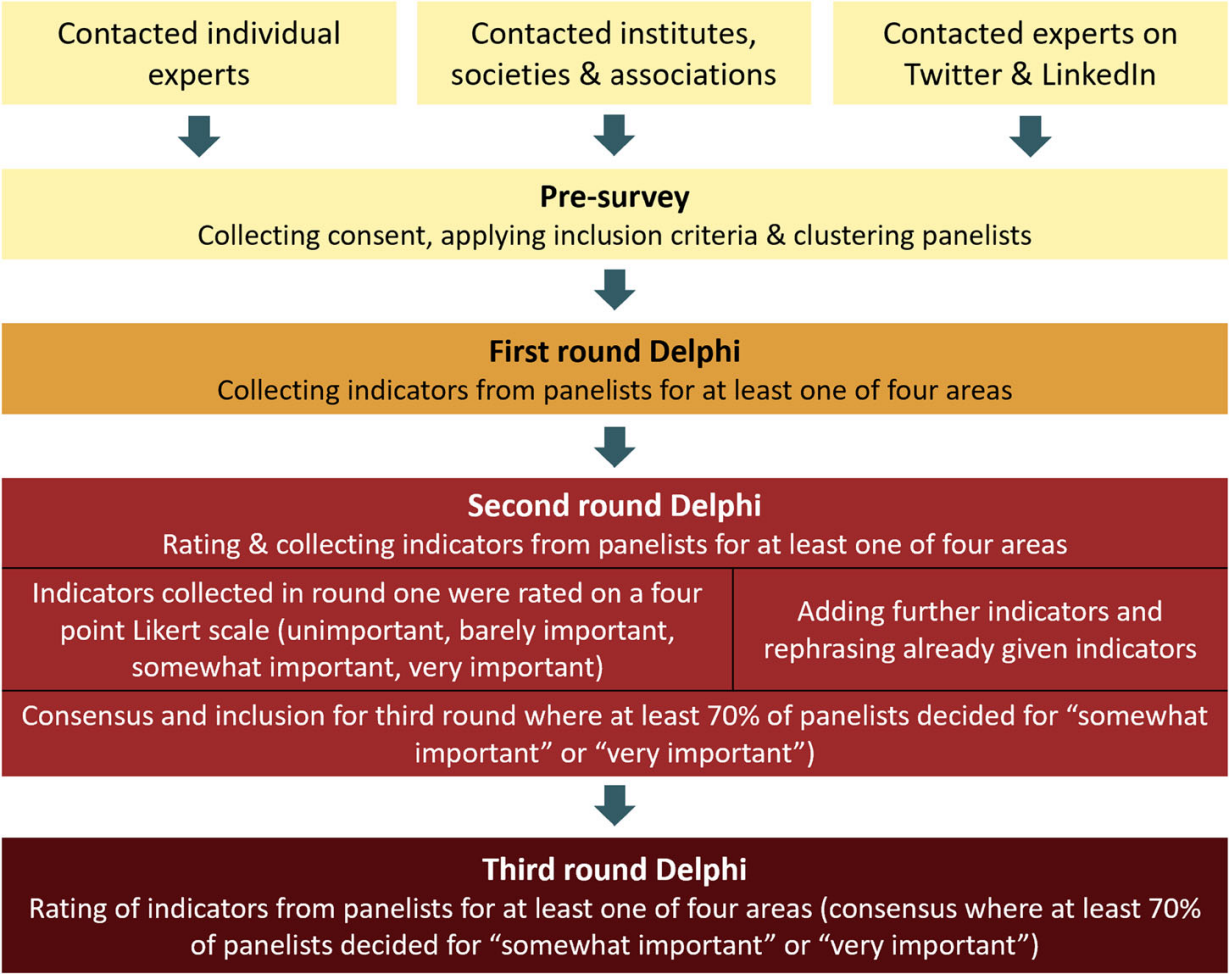
Structure of the Delphi study. The Delphi study was structured in a pre-survey to register interested experts, check for inclusion criteria, and assess interest in contributing to the four overarching domains. This round was followed by three official Delphi panels in which indicators and tools were proposed (round 1 and 2), rephrased (round 2), and rated on a four-point Likert scale regarding their importance for digital public health (round 2 and 3). Indicators and tools with at least 70% agreement on their importance (measured as the share of 3/4 and 4/4 votes on the scale) were included for the upcoming round.

The general structure of our Delphi is displayed in Fig. 4. This Delphi study consisted of one pre-survey (R0) to assess the eligibility of experts for participation as well as their socio-demographic and educational information. Further, all participants were electronically provided with data processing and protection information during R0. All experts provided electronic informed consent during R0 after being provided with sufficient electronic information to make an informed decision as to whether they want to take part in our study. As there is no explicit agreement on how many assessment cycles (survey rounds) are needed in a Delphi study^60,61^, this study consisted of three online panel rounds following R0 (R1–R3). All rounds were conducted through online questionnaires on the commercial platform QuestionPro (QuestionPro GmbH, Berlin, Germany) and piloted by persons with expertise in DiPH who did not belong to the research team. During each survey period, participants who registered in R0 but did not participate in the current survey round were reminded weekly via email to contribute.

During the first round (R1) from 16 May to 6 June 2022, all panelists were provided with the definition of public health, and DiPH used for this study (see Textbox 1). Participants then selected at least one of the four areas of interest and provided indicators to measure the maturity of DiPH systems from this perspective. Following the grounded theory approach by Glaser & Strauss^62^ we did not present existing indicators during R1. For research on interdisciplinary topics, it is crucial not to channel responses too much in advance but rather to be as open as possible to give representatives from different disciplines space to express their opinions^63^. Although presenting such criteria already for R1 might have led the participants in a desired direction and would have resulted in better comparisons for rating behavior, the disadvantages of this approach were seen as more prominent: Providing a list of developed indicators with the option to name additional ones might create a bias in answer categories and a loss of spontaneous response (as participants might not use the “other” option)^64,65^.

The clustered items from R1 were presented to the participants in R2 from 13 June to 27 June 2022. Experts were asked to rate the indicators and tools on a four-point Likert scale (unimportant to very important). Additionally, participants could select “I cannot rate this indicator due to a lack of expertise” if they felt an indicator was beyond their scope of expertise. The consensus was defined a priori if the item or tool was rated between 3–4 (important to very important) by at least 70% of the participants. This approach is commonly used in Delphi studies and suggested in gold-standard guidelines^54,66–70^. Further, we encouraged panelists to propose additional indicators or tools and rephrase them if necessary.

For R3 from 8 July to 15 September 2022, all indicators and tools that had received at least 70% consensus or were offered as alternatives in R2 were displayed for a final rating (same approach as in R2). This time, however, participants were unable to provide alternative formulations or comments.

All registered experts who met the inclusion criteria were invited to contribute to each Delphi round, even if they had not participated in the previous panel. Although such approaches might risk an attrition bias, we decided that this risk does not outweigh having a sufficient number of at least 15 experts contributing to each of the four domains during every panel. We conducted a balanced panel analysis to test if the ratings from experts who contributed to all three rounds differed significantly from the overall voting. This was not the case as displayed in Supplementary Information 3.

We did not apply strict exclusion rules as we expected that experts could meaningfully contribute to the further discussion even if they had missed one round (of all actively contributing experts, 30 participated in all three rounds). The rationale behind this approach was to not limit our cohort size further. Additionally, some Delphi studies start with already a pre-defined set of variables for the participants to rate. Due to this, we did not find it problematic that 10 participants only contributed to rounds 2 and/or 3 exclusively. Therefore, experts were encouraged to contribute to the following panels even when they did not contribute to the previous one.

### Panelists

In total, 346 experts were identified and contacted by the authors via email based on their position as an editor for an internationally published and peer-reviewed digital health or DiPH health journal (*n* = 183), as contact persons for digital health or DiPH institutions, associations, or networks (*n* = 73), and based on relevant publications or teaching relevant digital health or DiPH classes at universities (*n* = 163). The email contained information regarding why the study is conducted, how it will be conducted, why the experts were selected for participation, and a link to the questionnaire platform. During the study, we encouraged participants to contribute to every round, and sent multiple reminders to increase the participation rate. These approaches were aimed to minimize attrition bias and equalize recruitment by geographical region and field of expertise. The study was also advertised on social media (Twitter and LinkedIn). Further, we applied a snowball sampling method^71^ and asked contacted experts to share the invitation in their professional network.

Due to the lack of standards regarding the ideal number of experts per round^60^, we followed the RAND/UCLA Appropriateness Method User’s Manual^66^, which suggests a panel with 7 to 15 experts. We calculated a priori with a 50% dropout rate throughout our study. Therefore, in our study protocol, we decided to start the official Delphi study with R1 once at least 30 experts per domain who met the inclusion criteria confirmed their interest in participation in R0. We followed a criterion sampling strategy as displayed in Table 6. Although heterogeneous panels tend to find consensus slower than homogeneous groups^39,60,72,73^, we deemed this approach necessary to reflect the interdisciplinary nature of the topic and, therefore, invited experts with diverse backgrounds in geography and scientific disciplines. Our method is supported by other Delphi studies where a panel needed to achieve consensus on a broader and more diverse topic^39,72^. This is also the case for holistically assessing the maturity of DiPH systems.

**Table 6:** Inclusion criteria

Topic	Inclusion criteria
Educational Background	At least a Bachelor’s degree.
Professional Background	Computer Science, Epidemiology, Ethics, Law, Medical Informatics, Medicine, Politics, Public Health, Sociology, or comparable discipline
Years of experience in the discipline	At least three years
Years of experience in developing, designing, implementing, regulating, or evaluating digital (public) health tools	At least one year
Age	At least 21 years old
Internet	Access to the Internet during the study period
Language	Ability to read, write, and understand English

### Data collection and analysis

The pre-survey and the official Delphi study were conducted anonymously through online questionnaires on the commercial platform QuestionPro. Further research shows that anonymous approaches in Delphi studies empower participants to present their ideas more freely while reducing the risk of individual panelists dominating the discussion^74,75^. The survey was piloted by four persons with expertise in DiPH who did not belong to the research team to reduce the risk of misinterpretation of statements and instructions.

**Fig. 5:**
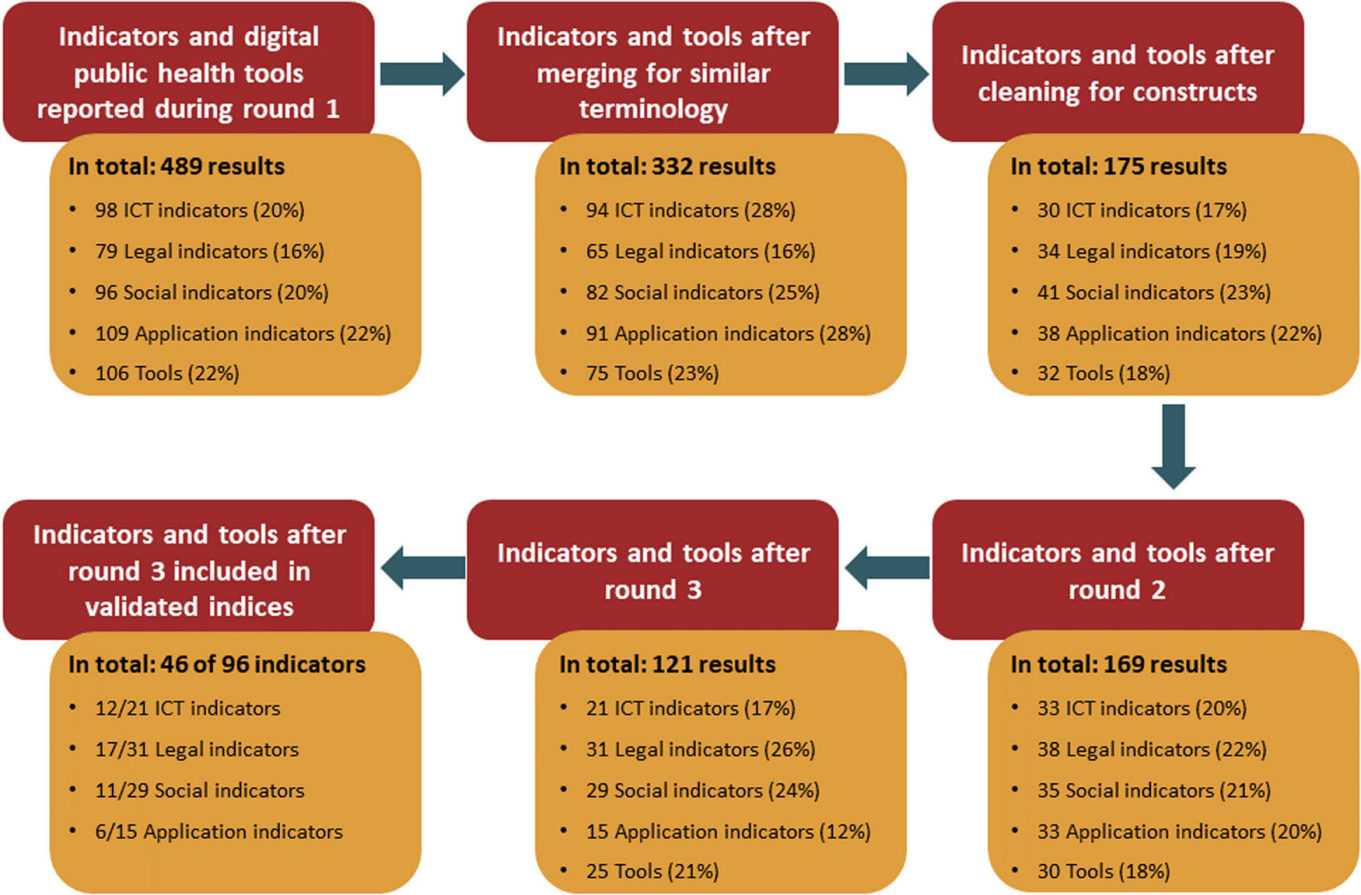
Data consolidation process and change in indicator and tool numbers during the Delphi study. Initially, 303 indicators and 106 digital public health tools were proposed during round 1. After data cleaning, this resulted in 136 indicators and 32 tools which were presented during round 2. As new indicators and tools were proposed during round 2, 135 indicators and 30 tools were kept for the final round. Here, 96 indicators and 25 tools were agreed upon by the participants to be important for digital public health. Of these indicators, only 48% were covered by already existing indices.

We qualitatively assessed and clustered the given indicators and DiPH tools following the thematic analysis approach by Braun & Clarke^76^, displayed in Fig. 5. The clusters were developed from the empirical data given by the study participants (inductive approach) and merged during the Delphi process when clusters included less than three indicators or tools.

### Sub-group and sensitivity analysis

We assumed that different understandings of usable and practical indicators could arise based on national regulations or the scientific background of participating experts. Therefore, we conducted a sub-group analysis to see if the share of “somewhat important” and “very important” replies differed between over-represented sub-groups (namely participants from Germany and experts in public health) and the rest of the participating experts. A conflict was defined if one sub-group showed at least 70% agreement on keeping an indicator (70% of the participants choosing “somewhat important” or “very important”) while the other group had a lower overall rating of somewhat or very important. Building on this evaluation, we conducted a sensitivity analysis to see if the overall share of these ratings for each of the domains was significantly biased by decisions from the two sub-groups. After testing for normal distribution through the Kolmogorov-Smirnov test, we conducted a two-sided Gaussian test with alpha 5%. The results of this analysis are displayed in Supplementary Information 2, 3.

### Ethical approval

This Delphi study sought to identify essential indicators to map digital health system maturity. We did not intend to gather any personal information (beyond basic socioeconomic data for group characterization) from the international participants. All interested experts were provided with written electronic information regarding the study design, aim, data protection, and data processing. We actively collected electronic written consent as soon as invited panelists indicated their willingness to take part. As in other Delphi studies with experts where the core aim is to work towards consensus (e.g., Greenhalgh et al.^57^) formal ethics approval was deemed not necessary as explicit informed consent by participants to share their expertise was obtained by the research group.

### Reporting summary

Further information on research design is available in the Nature Research Reporting Summary linked to this article.

### Supplementary information

Supplementary Information

Reporting Summary

## Data Availability

The anonymized datasets generated during and/or analyzed during the current study are available from the corresponding author upon reasonable request.

## References

[1] The World Bank. World development report 2016. Digital dividends. (The World Bank, 2016).

[2] Marwaha JS, Landman AB, Brat GA, Dunn T, Gordon WJ (2022). Deploying digital health tools within large, complex health systems: key considerations for adoption and implementation. npj Digit. Med..

[3] Ricciardi W (2019). How to govern the digital transformation of health services. Eur. J. Public Health.

[4] Marques ICP, Ferreira JJM (2019). Digital transformation in the area of health: systematic review of 45 years of evolution. Health Technol..

[5] Gavrilov, G., Simov, O. & Trajkovik, V. Analysis of Digitalization in Healthcare: Case Study. In *ICT Innovations 2020. Machine Learning and Applications* (eds Dimitrova, V., Dimitrovski, I.). Communications in Computer and Information Science, Vol. 1316 (Springer, Cham, 2020).

[6] Colldén C, Hellström A (2022). From “Invented here” to “Use it everywhere!”: a Learning health system from bottom and/or top?. Learn Health Syst..

[7] Solimini R, Busardò FP, Gibelli F, Sirignano A, Ricci G (2021). Ethical and legal challenges of telemedicine in the era of the COVID-19 pandemic. Medicina.

[8] Azzopardi-Muscat N, Sørensen K (2019). Towards an equitable digital public health era: promoting equity through a health literacy perspective. Eur. J. Public Health.

[9] Scott Kruse C (2018). Evaluating barriers to adopting telemedicine worldwide: a systematic review. J. Telemed. Telecare.

[10] European Union. Assessing the impact of digital transformation of health services. Report of the Expert Panel on effective ways of investing in Health (EXPH). (Publications Office of the European Union, 2019).

[11] Thiel, R. et al. #SmartHealthSystems. *International comparison of digital strategies* (Bertelsmann Stiftung, 2019).

[12] World Health Organization (WHO) & International Telecommunication Union (ITU). *National eHealth Strategy Toolkit*. (WHO, 2012).

[13] Bahia, K. & Agnoletto, F. *Mobile Connectivity Index Methodology* (GSMA, 2022).

[14] cisco. *Cisco Digital Readiness Index*, https://www.cisco.com/c/en/us/about/csr/research-resources/digital-readiness.html (2023).

[15] Economist Impact, Nuclear Threat Initiative (NTI) & Johns Hopkins Bloomberg School of Public Health. *Global Health Security Index. GHS Index Methodology*. (NTI, 2021).

[16] European Commission. *The Digital Economy and Society Index (DESI). Key Indicators*, https://digital-agenda-data.eu/datasets/digital_agenda_scoreboard_key_indicators/indicators (2023).

[17] Global Development Incubator. *Global Digital Health Index. Indicators*, http://index.digitalhealthindex.org/indicators_info (2023).

[18] International Telecommunication Union (ITU). *The ICT Development Index (IDI): conceptual framework and methodology*, https://www.itu.int/en/ITU-D/Statistics/Pages/publications/mis2017/methodology.aspx (2017).

[19] International Telecommunication Union (ITU). ITU-D Cybersecurity Program Global Cybersecurity Index – GCIv5 Reference Model (Methodology), 2023).

[20] Legatum Institute. *The 2023 Legatum Prosperity Index. A tool for transformation*. Vol. 16th edition (Legatum Institute Foundation, 2023).

[21] Partnership on Measuring ICT for Development. *Core List of ICT Indicators* (ITU, 2022).

[22] Portulans Institute. The Network Readiness Index 2022. Stepping into the new digital era. How and why digital natives will change the world (Portulans Institute, 2022).

[23] United Nations (UN). E-Government Survey 2022. The Future of Digital Government. (UN, 2022).

[24] World Bank. *Digital Dividends*. (World Bank, 2016).

[25] World Bank. *World Development Indicators*, https://data.worldbank.org/indicator?tab=all (2023).

[26] European Commission. *DESI composite index*, https://digital-decade-desi.digital-strategy.ec.europa.eu/datasets/key-indicators/indicators (2024).

[27] Mechael, P. & Edelman, J. K. *The State of Digital Health 2019. Global Digital Health Index* (Global Development Incubator, 2019).

[28] Hecht, V. J. & Hribernik, N. in *Praxis der Sinus-Milieus. Gegenwart und Zukunft eines modernen Gesellschafts- und Zielgruppenmodells* (eds Barth, B., Flaig, B. B., Schäuble, N., Tautscher, M.) 103–112 (Springer VS, 2018).

[29] Great Britain Committee of Inquiry into the Future Development of the Public Health Function. Public health in England: The report of the Committee of Inquiry into the Future Development of the Public Health Function. (H.M.S.O., 1988).

[30] Eysenbach G (2001). What is e-health?. J. Med Internet Res..

[31] Maass L, Pan CC, Freye M (2022). Mapping digital public health interventions among existing digital technologies and internet-based interventions to maintain and improve population health in practice: protocol for a scoping review. JMIR Res. Protoc..

[32] Hasson F, Keeney S, McKenna H (2000). Research guidelines for the Delphi survey technique. J. Adv. Nurs..

[33] Dalkey N, Helmer O (1963). An experimental application of the DELPHI method to the use of experts. Manag. Sci..

[34] Hsu C-C, Sandford BA (2007). The Delphi technique: making sense of consensus. Pract. Assess. Res. Eval..

[35] Fusfeld, A. R. *Research Program on the Management of Science and Technology: The Delphi Technique, Survey and Comment* (Massachusetts Institute of Technology, 1971).

[36] Landeta J (2006). Current validity of the Delphi method in social sciences. Technol. Forecast. Soc. Change.

[37] McKenna HP (1994). The Delphi technique: a worthwhile research approach for nursing?. J. Adv. Nurs..

[38] Cresswell KM (2013). Global research priorities to better understand the burden of iatrogenic harm in primary care: an international Delphi exercise. PLOS Med..

[39] Boulkedid R, Abdoul H, Loustau M, Sibony O, Alberti C (2011). Using and reporting the Delphi method for selecting healthcare quality indicators: a systematic review. PLoS One.

[40] Lam K, Iqbal FM, Purkayastha S, Kinross JM (2021). Investigating the ethical and data governance issues of artificial intelligence in surgery: protocol for a Delphi study. JMIR Res. Protoc..

[41] Sinha IP, Smyth RL, Williamson PR (2011). Using the Delphi technique to determine which outcomes to measure in clinical trials: recommendations for the future based on a systematic review of existing studies. PLOS Med..

[42] Price-Haywood EG, Arnold C, Harden-Barrios J, Davis T (2023). Stop the divide: facilitators and barriers to uptake of digital health interventions among socially disadvantaged populations. Ochsner J..

[43] Ronquillo C, Currie L (2012). The digital divide: Trends in global mobile and broadband Internet access from 2000-2010. Ni 2012 (2012).

[44] Wienert J, Jahnel T, Maaß L (2022). What are Digital public health interventions? First steps toward a definition and an intervention classification framework. J. Med. Internet Res..

[45] Tiffin N, George A, LeFevre AE (2019). How to use relevant data for maximal benefit with minimal risk: digital health data governance to protect vulnerable populations in low-income and middle-income countries. BMJ Glob. Health.

[46] Public Health England. *Digital-first public health: Public Health England’s digital strategy*, https://www.gov.uk/government/publications/digital-first-public-health/digital-first-public-health-public-health-englands-digital-strategy (2017).

[47] Wong BLH (2022). The dawn of digital public health in Europe: implications for public health policy and practice. Lancet Reg. Health—Eur..

[48] U.S. Food & Drug Administration (FDA). *What is Digital Health?*, https://www.fda.gov/medical-devices/digital-health-center-excellence/what-digital-health (2020).

[49] Winslow CEA (1920). The untilled fields of public health. Science.

[50] Zeeb H, Pigeot I, Schuz B, Leibniz-WissenschaftsCampus Digital Public Health, B. (2020). Digital public health-an overview. Bundesgesundheitsblatt Gesundheitsforschung Gesundheitsschutz.

[51] Anomaly J (2023). What is public health? public goods, publicized goods, and the conversion problem. Public Choice.

[52] Abdalla SM, Maani N, Ettman CK, Galea S (2020). Claiming health as a public good in the post-COVID-19 era. Development (Rome).

[53] Diamond IR (2014). Defining consensus: a systematic review recommends methodologic criteria for reporting of Delphi studies. J. Clin. Epidemiol..

[54] Jünger S, Payne SA, Brine J, Radbruch L, Brearley SG (2017). Guidance on Conducting and REporting DElphi Studies (CREDES) in palliative care: Recommendations based on a methodological systematic review. Palliat. Med..

[55] Dreesen M (2013). Quality of care for cancer patients on home parenteral nutrition: development of key interventions and outcome indicators using a two-round Delphi approach. Support. Care Cancer.

[56] Douillet D (2020). Outpatient management or hospitalization of patients with proven or suspected SARS-CoV-2 infection: the HOME-CoV rule. Intern. Emerg. Med..

[57] Greenhalgh T (2020). What items should be included in an early warning score for remote assessment of suspected COVID-19? qualitative and Delphi study. BMJ Open.

[58] Guckenberger M (2020). Practice recommendations for lung cancer radiotherapy during the COVID-19 pandemic: An ESTRO-ASTRO consensus statement. Radiother. Oncol..

[59] Den Broeder L, Devilee J, Van Oers H, Schuit AJ, Wagemakers A (2018). Citizen Science for public health. Health Promot. Int..

[60] Donohoe HM, Needham RD (2009). Moving best practice forward: Delphi characteristics, advantages, potential problems, and solutions. Int. J. Tour. Res..

[61] Erffmeyer RC, Erffmeyer ES, Lane IM (1986). The Delphi technique: an empirical evaluation of the optimal number of rounds. Group Organ. Stud..

[62] Glaser, B. & Strauss, A. *Discovery of Grounded Theory: Strategies for Qualitative Research* (Transaction Publishers, 1999).

[63] Ming X, MacLeod M, van der Veen J (2023). Construction and enactment of interdisciplinarity: a grounded theory case study in Liberal Arts and Sciences education. Learn. Cult. Soc. Interact..

[64] Sbaraini A, Carter SM, Evans RW, Blinkhorn A (2011). How to do a grounded theory study: a worked example of a study of dental practices. BMC Med. Res. Methodol..

[65] Vinten G (1995). Open versus closed questions—an open issue. Manag. Decis..

[66] Fitch, K. et al. *The RAND/UCLA Appropriateness Method User’s Manual* (RAND Corporation, 2001).

[67] Lam K (2022). A Delphi consensus statement for digital surgery. NPJ Digit. Med..

[68] Krasuska M (2020). Technological capabilities to assess digital excellence in hospitals in high performing health care systems: International eDelphi exercise. J. Med. Internet Res..

[69] Regan M (2022). Policies and interventions to reduce harmful gambling: an international Delphi consensus and implementation rating study. Lancet Public Health.

[70] Fink A, Kosecoff J, Chassin M, Brook RH (1984). Consensus methods: characteristics and guidelines for use. Am. J. Public Health.

[71] Goluchowicz K, Blind K (2011). Identification of future fields of standardisation: an explorative application of the Delphi methodology. Technol. Forecast. Soc. Change.

[72] Nasa P, Jain R, Juneja D (2021). Delphi methodology in healthcare research: How to decide its appropriateness. World J. Methodol..

[73] Penna A (1997). Do different physicians’ panels reach similar conclusions? A case study on practice guidelines for limited surgery in breast cancer. Eur. J. Public Health.

[74] Keeney S, Hasson F, McKenna HP (2001). A critical review of the Delphi technique as a research methodology for nursing. Int. J. Nurs. Stud..

[75] Glöggler M, Ammenwerth E (2021). Improvement and evaluation of the TOPCOP taxonomy of patient portals: Taxonomy-Evaluation-Delphi (TED) approach. J. Med. Internet Res..

[76] Braun V, Clarke V (2022). Conceptual and design thinking for thematic analysis. Qualit. Psychol..

[77] Odone A, Buttigieg S, Ricciardi W, Azzopardi-Muscat N, Staines A (2019). Public health digitalization in Europe: EUPHA vision, action and role in digital public health. Eur. J. Public Health.

