# Supplementary material for: International perspectives on measuring national digital public health system maturity through a multidisciplinary Delphi study
Source: NPJ Digit Med. 2024 Apr 12;7:92. doi: 10.1038/s41746-024-01078-9 (PMC11014962; doi:10.1038/s41746-024-01078-9)
Supplement: Supplementary file 1 — Supplementary Information [file 41746_2024_1078_MOESM1_ESM.pdf]

Supplementary File 1: Indicators given per round including rating for round 2 and 3

Supplementary Table 1. Information-Communication-Technology (ICT) requirements

	Round 2				Round 3			
**Indicators on expenses, investment and workforce**	**n**	**Could not rate**	**Rank 1-2***	**Rank 3-4***	**n**	**Could not rate**	**Rank 1-2***	**Rank 3-4***
I01: The ratio between private health expenditure and household disposable income health expenditure	20	2	44%	56%[$]	-	-	-	-
I02: The average prices for fixed broadband Internet connections per month	20	0	25%	75%	15	0	27%	73%
I03: The average prices for pre-paid contracts for mobile phones per month	20	0	30%	70%	15	0	40%	60%[$]
I04: The annual telecommunications revenue (% of gross domestic product)	20	1	68%	32%[$]	-	-	-	-
I05: The annual telecommunications investment (% of revenue)	20	1	42%	58%[$]	-	-	-	-
I06: The percentage of health expenditure in gross domestic product (public and private)	20	1	26%	74%	15	1	36%	64%[$]
I07: The annual spending by the government on the information-communication-infrastructure	20	0	20%	80%	15	0	13%	87%
I08: The level of feedback and communication with various publics	20	1	53%	47%[$]	-	-	-	-
I09: The level of education	20	2	11%	89%	15	0	33%	67%[$]
I09N: The level of training in the use and billing of digital public health services by health care professionals	1	-	-	-[+]	15	0	13%	87%
I10: The number of feedbacks from patients	20	1	5%	95%	15	1	36%	64%[$]
I11: The number of skilled professionals in data science	20	1	5%	95%	15	1	13%	87%
I12: The proportion of the total business sector workforce involved in the information-telecommunication-technology sector	20	1	42%	58%[$]	-	-	-	-
I13: The number of participants in training programs for using digital public health tools by groups (e.g., healthcare workers, patients, or family caregivers)	20	0	15%	85%	15	1	43%	57%[$]
I13N1: The average number of participants in digital public health tool training programs in a year by different target groups (e.g., health professionals or patients)	1	-	-	-[+]	15	1	7%	93%
I13N2: The average number of digital public health tool training provided to different target groups	1	-	-	-[+]	15	1	21%	79%
**Indicators on the availability of the Internet and Internet-enabled devices such as PCs, phones & tablets**								
I14: The share of households/hospitals/physician offices connected to the Internet (by bandwidth)	20	0	5%	95%	15	15	13%	93%
I15: The personal computers per 100 people	20	1	42%	58%[$]	-	-	-	-
I16: The secure Internet servers (per 1 million people)	20	2	39%	61%[$]	-	-	-	-
I17: The share of households, hospitals & physician offices with a computer	20	0	5%	95%	15	1	29%	71%
I17N: The share of specific locations that are equipped with at least one Internet-connected device by location (e.g., households, hospitals or outpatient physician's offices)	1	-	-	-[+]	15	1	0%	100%
I18: The number of Internet-connected devices per 100 people by the device (e.g., computers, smartphones, tablets, or notebooks)	1	-	-	-[+]	15	0	27%	73%
I19: The percentage of the overall population with access to the Internet by speed	1	-	-	-[+]	15	0	7%	93%
I20: The percentage of the overall population with access to computers	1	-	-	-[+]	15	9	27%	73%

Indicators on the availability of the Internet and Internet-enabled devices such as PCs, phones & tablets	Round 2				Round 3			
	n	Could not rate	Rank 1-2*	Rank 3-4*	n	Could not rate	Rank 1-2*	Rank 3-4*
I21: The percentage of the population covered by at least a 3G mobile network	20	1	21%	79%	15	0	13%	87%
I22: The telephone lines per 100 people	20	1	68%	32%$	-	-	-	-
I23: The percentage of the overall population with access to smartphones	1	-	-	-+	15	0	0%	100%
**Indicators on interoperability and infrastructure**								
I24: The share or paperless hospitals of all hospitals, when delivering and managing patient care	20	1	11%	89%	15	1	21%	79%
I24N: The percentage of all hospitals that use electronic documentation systems for patient care	1	-	-	-+	15	1	0%	100%
I25: The availability of health information exchange platforms	20	0	0%	100%	15	0	7%	93%
I26: The existence of a decentralized infrastructure that leaves the data where they originated & accessible based on consent and ID approval	20	1	11%	89%	15	1	29%	71%
I26N1: The existence of a decentralized data infrastructure	1	-	-	-+	15	2	38%	62%$
I26N2: The existence of the possibility to access personal health data based on consent and ID approval	1	-	-	-+	15	1	0%	100%
I27: The degree of existing telematic infrastructure	20	2	17%	83%	15	1	7%	93%
I28: The degree of interoperability of health systems	20	1	5%	95%	15	1	0%	100%
I29: The degree of compliance of specific interventions with the data exchange and interoperability standards	20	1	5%	95%	15	2	8%	92%
I30: The existence of interoperable data end-to-end encryption	20	3	18%	82%	15	2	15%	85%
I31: The number of integrated digital systems in the healthcare system	1	-	-	-+	15	1	28%	72%
**Indicators on other topics**								
I32: The percentage of operators with a waiting time less than the guaranteed maximum response time	20	3	24%	76%	15	4	36%	64%$
I33: The number of adverse events	20	5	27%	73%	15	2	31%	69%$
I33N: The number of technical malfunctions per digital public health tool reported per year	1	-	-	-+	15	1	14%	86%
I34: The percentage of users with first hospital specialty appointments performed within the reference time	20	5	40%	60%$	-	-	-	-
I35: The degree of internationalization	20	3	47%	53%$	-	-	-	-

*The calculation includes only those experts, who voted on the Likert scale unimportant (1), barely important (2), somewhat important (3), and very important (4). Those that selected the “I can’t rate this indicator due to a lack of expertise” were excluded from the consensus calculation.
+Indicators or digital public health (DiPH) tools that were first given (as alternative formulations) during the second panel round.
$Indicators or DiPH tools with less than 70% votes for “somewhat important” or “very important”, which were, therefore, excluded.

Interventions with a share of less than 70% for “somewhat important” or “very important” votes were excluded for the next panel round.
Regarding alternative formulations of indicators, only the phrasing with the highest share of “somewhat important” or “very important” votes were kept.

Supplementary Table 2. Legal framework and political support

Indicators on health data (access, exchange, security)	Round 2 n	Round 2 Could not rate	Round 2 Rank 1-2*	Round 2 Rank 3-4*	Round 3 n	Round 3 Could not rate	Round 3 Rank 1-2*	Round 3 Rank 3-4*
L01: The percentage of user consent to health data	22	2	15%	85%	17	1	25%	75%
L02: The coverage of international standards in stored data	22	4	22%	78%	17	0	24%	76%
L03: The existence of a legal framework for exchanging health data digitally between different stakeholders	22	1	0%	100%	17	0	0%	100%
L04: The degree of political support in data transfer & exchange	22	1	19%	81%	17	0	24%	76%
L05: The existence of a legal framework for the secondary use of health data	22	1	10%	90%	17	0	6%	94%
L06: The existence of regulations for access to health data through electronic health records	22	1	5%	95%	17	2	0%	100%
L07: The existence of legislation regulating the interaction between the digital data of a patient's health and the data of bioinformatics / genetic information of the biomaterials of this patient	1	-	-	-[+]	17	3	21%	79%
L08: The level of encryption of personal and health data	22	2	10%	90%	17	0	18%	82%
L09: The ratio of encrypted records to the total number of records	22	3	26%	74%	17	1	31%	69%[$]
L10: The existence of regulations that the digital data collected via electronic health applications are only stored on one's own electronic health card	22	5	24%	76%	17	2	33%	67%[$]
L11: The coverage of metadata labels in sensitive data	22	6	13%	88%	17	3	21%	79%
L12: The number of critical failures in security points	22	3	5%	95%	17	1	13%	88%
L13: The number of weaknesses in security perimeters	22	3	11%	89%	17	3	14%	86%
L14: The level of data security	22	3	0%	100%	17	2	13%	87%
**Indicators on digital assets**								
L15: The existence of a policy or legislative reform to allow live-time digital asset	22	6	19%	81%	17	1	38%	62%[$]
L16: The existence of policy/legislative reform to allow access to all digital assets that would fit into the World Health Organizations' definition of health and may be considered non-health assets (education, Social system, work, income, etc.) traditionally	22	4	28%	72%	17	1	50%	50%[$]
L17: The existence of a policy or legislative reform to allow access to digital assets	22	5	18%	82%	17	1	25%	75%
**Indicators on the digital public health strategy**								
L18: The existence of political briefs/policies that mention digital health	22	1	33%	67%[$]	-	-	-	-
L19: The existence of a political strategy to digitalize the healthcare system	22	2	5%	95%	17	0	12%	88%
L20: The existence of a digital public health strategy within the governmental health strategy	22	2	10%	90%	17	0	12%	88%
L21: The existence of guidelines for planning & implementing digital public health tools	22	1	19%	81%	17	0	6%	94%
L22: The level of harmonization of legal regulations in electronic health	22	1	5%	95%	17	0	35%	65%[$]
L23: The existence of legal supervision of the implementation of national digital public health programs	22	1	19%	81%	17	0	12%	88%
L24: The existence of a department for digital health in the health ministry	22	1	19%	81%	17	1	19%	81%
L25: The existence of regulation on the function of electronic health products (e.g., Medical Devices Act)	22	2	20%	80%	17	1	6%	94%

	Round 2				Round 3			
**Indicators on the digital public health strategy**	**n**	**Could not rate**	**Rank 1-2***	**Rank 3-4***	**n**	**Could not rate**	**Rank 1-2***	**Rank 3-4***
L26: The existence of a legal right for citizens to be provided with digital health services	1	-	-	-[+]	17	2	27%	73%
L27: The existence of a digital public health policy engaged with the protection of fundamental rights of vulnerable groups (e.g., children, adolescents, mentally disabled people)	1	-	-	-[+]	17	0	24%	76%
L28: The existence of a policy to promote innovation and the development of digital tools in the public health system	1	-	-	-[+]	17	0	18%	82%
**Indicators on finances and reimbursement**								
L29: The existence of a public funding scheme for digital public health interventions on a regional and national level	22	2	30%	70%	17	0	18%	82%
L30: The existence of investment and reimbursement possibilities from the government	22	3	21%	79%	17	0	12%	88%
L31: The annual spending by the government as support for the implementation of digital technologies in healthcare	22	2	15%	85%	17	1	25%	75%
L32: The number of digital applications remunerated	22	4	56%	44%[$]	-	-	-	-
L33: The existence of financial incentives for health professionals to participate in offering digital public health services	22	2	25%	75%	17	0	18%	82%
**Indicators on other topics**								
L34: The availability of legal tools, such as contracts templates	22	3	42%	58%[$]	-	-	-	-
L35: The rate of Direct Public Offering (DPO) approval and other activities	22	11	82%	18%[$]	-	-	-	-
L36: The existence of a law/regulation on prevention on the regional/national level	22	1	38%	62%[$]	-	-	-	-
L37: The efficiency of the alarm system at all levels of healthcare	22	6	44%	56%[$]	-	-	-	-
L38: The number of civic bodies that have the autonomy to organize the health care services at least at the primary and secondary levels of care (decentralization component)	22	3	68%	32%[$]	-	-	-	-
L39: The existence of an action plan to prevent discrimination based on race, gender, sexual orientation & age in digital health services	1	-	-	-[+]	17	1	38%	63%[$]
L40: The existence of a statutory provision that places restrictions and quarantine periods on public health leadership professionals for future private digital health activities after they have ceased their previous public service activities	1	-	-	-[+]	17	2	60%	40%[$]
L41: The existence of legal liability for public health managers for digital health contracts that harm patients or the public interest	1	-	-	-[+]	17	2	27%	73%
L42: The existence of policy standards for transparency and the protection of fundamental rights in using artificial intelligence in digital public health	1	-	-	-[+]	17	0	6%	94%
L43: The level of transparency and accessibility of digital health contracts to public oversight and law enforcement agencies for anti-corruption purposes	1	-	-	-[+]	17	0	29%	71%
L44: The effectiveness of informed patient consent for using personal health data in adult patients	1	-	-	-[+]	17	0	24%	76%
L45: The existence of unique procedures to protect children, adolescents and mentally ill patients who are unable to give their consent	1	-	-	-[+]	17	0	24%	76%

Supplementary Table 3. Social willingness and capability

	Round 2				Round 3			
**Indicators on potential and actual users**	**n**	**Could not rate**	**Rank 1-2***	**Rank 3-4***	**n**	**Could not rate**	**Rank 1-2***	**Rank 3-4***
S01: The number of people that are willing to use a digital public health tool or participate in a digital public health intervention	19	0	5%	95%	16	0	12%	88%
S02: The number of potential users of the same digital public health tool	19	0	21%	79%	16	1	13%	87%
S03: The number of potential users of a digital public health tool who have adequate access to the web	19	1	11%	89%	16	0	19%	81%
S04: The share of the eligible population who have used at least one digital public health intervention for routine care and health promotion in the previous year	19	0	26%	74%	16	0	13%	88%
S05: The share of the eligible population who have signed up (i.e., have an account) for at least one digital public health intervention focused on routine care and health promotion	19	0	32%	68%[$]	-	-	-	-
S06: The number of patients using apps to interact with local services	19	0	16%	84%	16	0	12%	88%
S07: The number of digital contacts	19	2	35%	65%[$]	-	-	-	-
S08: The adherence of users to the intervention (in percentage)	19	2	29%	71%	16	1	27%	73%
S09: The ratio of electronic health records to the total number of user records	19	3	19%	81%	16	1	27%	73%
S10: The number of patients enrolled after communication campaigns	19	0	37%	63%[$]	-	-	-	-
S11: The number of patients enrolled in app use	19	0	26%	74%	16	0	63%	38%[$]
S11N1: The number of patients enrolled in the use of specific health apps	1	-	-	-[+]	16	0	25%	75%
S11N2: The share of the population that uses any health or medical app by reason (e.g., health promotion, wellness, tracing)	1	-	-	-[+]	16	0	19%	81%
S12: The number of digital public health professionals	1	-	-	-[+]	16	1	24%	76%
S13: The number of patients interacting with apps	19	0	32%	68%[$]	-	-	-	-
S14: The number of digital platforms used and views reach and interaction	19	0	37%	63%[$]	-	-	-	-
S15: The number of posts placed on digital platforms	19	0	68%	32%[$]	-	-	-	-
S16: The utilization rate of specific groups per digital public health intervention (by health insurance claims data)	19	2	12%	88%	16	2	43%	57%[$]
**Indicators on awareness, trust & motivation**								
S17: The perceived usefulness of specific digital public health tools by groups on a Likert scale	19	2	24%	76%	16	1	21%	79%
S18: The share of the population that trusts digital health services	19	0	11%	89%	16	0	13%	88%
S19: The motivation to access electronic health services by groups of people	19	1	22%	78%	16	1	27%	73%
S20: The awareness of health professionals about the value of data and the possibility of their use by information-telecommunication-technology	19	0	11%	89%	16	0	13%	88%
S21: The awareness of groups of people that the intervention exists (by target groups)	19	0	21%	79%	16	0	6%	94%
S22: The level of trust in the government by different groups of the population	1	-	-	-[+]	16	0	38%	63%[$]
S23: The satisfaction rate	19	3	26%	74%	16	1	47%	53%[$]
S23N1: The self-reported satisfaction rate with digital public health intervention by user group	1	-	-	-[+]	16	0	25%	75%
S23N2: The share of the population that is more satisfied with using the digital public health intervention compared to standard care	1	-	-	-[+]	16	0	19%	81%

Indicators on literacy	Round 2 n	Could not rate	Rank 1-2*	Rank 3-4*	Round 3 n	Could not rate	Rank 1-2*	Rank 3-4*
S24: The average level of digital literacy by different target groups on a Likert Scale	19	0	11%	89%	16	1	7%	93%
S25: The average level of digital health literacy by different target groups on a Likert Scale	19	1	6%	94%	16	1	13%	87%
S26: The average level of health literacy by different target groups on a Likert Scale	19	2	24%	76%	16	1	13%	87%
S27: The average level of information-telecommunication-technology skills by groups of people	19	1	11%	89%	16	0	12%	88%
**Indicators on use of smart- and cellular phones**								
S28: The share of the population that uses a smartphone by different target groups	19	0	5%	95%	16	0	19%	81%
S29: The share of the population that owns a smartphone by different target groups	19	0	16%	84%	16	0	25%	75%
S30: The smartphone penetration rate versus the use of mobile devices for routine care and health promotion in the population	19	1	5%	95%	16	0	13%	87%
S31: The mobile cellular subscriptions per 100 people	19	1	56%	44%[$]	-	-	-	-
S32: The proportion of healthcare users using smartphones and other digital devices	19	1	17%	83%	16	0	19%	81%
**Indicators on use of the Internet**								
S33: The active mobile-broadband subscriptions per 100 inhabitants	19	0	25%	75%	16	2	8%	92%
S34: The fixed broadband Internet subscriptions per 100 inhabitants	19	1	26%	74%	16	0	20	80%
S35: The percentage of Internet users in the country	19	0	5%	95%	16	0	13%	87%
S36: The share of the population that uses the Internet for gathering health information by different target groups	19	0	11%	89%	16	0	19%	81%
S37: The number of searches for specific digital public health interventions measured on Google Trends	1	-	-	-[+]	16	0	25%	75%
**Indicators on other topics**								
S38: The number of various health promotion campaigns led by students/youth	19	0	58%	42%[$]	-	-	-	-
S39: The number of professional bodies consulted for patient-provider interactions regarding the service of routine care	19	1	56%	44%[$]	-	-	-	-
S40: The number of patients contacted to obtain feedback on routine care for chronic diseases	19	1	39%	61%[$]	-	-	-	-
S41: The degree of impact of communication campaigns	19	2	53%	47%[$]	-	-	-	-
S42: The number of disease-specific patient groups consulted for discussing care and treatment modalities in chronic diseases	19	1	28%	72%	16	3	38%	62%[$]
S43: The number of prospective parents reached out for counseling on child immunization	19	1	56%	44%[$]	-	-	-	-
S44: The number of institutions (schools/workplaces and others) that develop mechanisms through the consultative process (with children/employees) for promoting/adopting the government-sponsored/facilitated preventive physical activities	19	2	41%	59%[$]	-	-	-	-

*The calculation includes only those experts, who voted on the Likert scale unimportant (1), barely important (2), somewhat important (3), and very important (4). Those that selected the "I can't rate this indicator due to a lack of expertise" were excluded from the consensus calculation.

[+]Indicators or digital public health (DiPH) tools that were first given (as alternative formulations) during the second panel round.

[$]Indicators or DiPH tools with less than 70% votes for "somewhat important" or "very important", which were, therefore, excluded.

Interventions with a share of less than 70% for "somewhat important" or "very important" votes were excluded for the next panel round.
Regarding alternative formulations of indicators, only the phrasing with the highest share of "somewhat important" or "very important" votes were kept.

Supplementary Table 4. Degree of Application

Indicators on access to the service and information	Round 2				Round 3			
	n	Could not rate	Rank 1-2*	Rank 3-4*	n	Could not rate	Rank 1-2*	Rank 3-4*
A01: The number of patients reached through telemedicine	31	1	33%	67%$	-	-	-	-
A02: The availability of digital information on public health interventions as national prevention programs	31	1	15%	85%	26	0	23%	77%
A02N1: The share of available digital information on public health interventions	1	-	-	-+	26	0	31%	69%$
A02N2: The availability of reliable information on specific digital public health services	1	-	-	-+	26	0	8%	92%
A03: The number of individuals reached through digital public health system (via mobile messaging) for health promotion information/communication	31	1	33%	67%$	-	-	-	-
A04: The percentage of the overall population with access	31	1	8%	92%	26	1	32%	68%$
A04N: The percentage of the overall population with access to the digital public health tool	1	-	-	-+	26	0	0%	100%
A05: The availability of reliable health information in a digital format	31	2	15%	85%	26	1	16%	84%
A06: The proportion of persons who cannot access their digital data and the provision of alternative access	1	-	-	-+	26	0	12%	88%
**Indicators on secondary use of health data**								
A07: The share of patient health data used for monitoring or evaluating the healthcare system	31	2	15%	85%	26	1	28%	72%
A07N1: The share of patient health data used for evaluating healthcare services	1	-	-	-+	26	0	27%	73%
A07N2: The share of population health data used for public health monitoring	1	-	-	-+	26	0	27%	73%
A08: The percentage run-throughs completed (re)use of data	31	9	42%	58%$	-	-	-	-
A09: The degree of data interoperability	31	2	4%	96%	26	2	25%	75%
A09N: The degree of technical/syntactic/semantic interoperability	1	-	-	-+	26	1	9%	91%
A10: The degree of data linkage	31	3	12%	88%	26	1	28%	72%
A10N: The availability of a unique identifier to link health data for a person between different digital public health tools/platforms	1	-	-	-+	26	0	4%	96%
A11: The degree of data accessibility	31	2	8%	92%	26	0	4%	96%
**Indicators on service implementation**								
A12: The degree to which an intervention is established (e.g., local pilot, communal, regional, national)	31	5	13%	87%	26	0	23%	77%
A12N: The level to which an intervention is established (e.g., local pilot, communal, regional, national)	1	-	-	-+	26	0	39%	61%$
A13: The degree to which the intervention complements existing workflows or duplicates them	31	3	25%	75%	26	4	50%	50%$
A13N: The extent of redundancy in workflows created by introducing digital public health tools/interventions to complement existing workflows	1	-	-	-+	26	2	25%	75%
A14: The percentage of health promotion programs integrated into national health system digital platforms	31	1	33%	67%$	-	-	-	-
A15: The proportion of (specific) public health services delivered via digital technologies (may be disaggregated by technology type)	31	2	35%	65%$	-	-	-	-
A16: The proportion of digital public health interventions considering health equity in their planning, implementation & evaluation	31	2	15%	85%	26	1	8%	92%
A17: The proportion of public health services implementing at least one digital technology intervention	31	3	52%	48%$	-	-	-	-

Indicators on service implementation	Round 2 n	Round 2 Could not rate	Round 2 Rank 1-2*	Round 2 Rank 3-4*	Round 3 n	Round 3 Could not rate	Round 3 Rank 1-2*	Round 3 Rank 3-4*
A18: The number of regulated digital health services included in routine care	31	1	22%	78%	26	0	27%	73%
A19: The total number of records	31	13	39%	61%$	-	-	-	-
**Indicators on traditional health services and prevention**								
A20: The number of patients recommended by their treating physician to adopt regular physical activities (such as joining fitness club/swimming)	31	1	41%	59%$	-	-	-	-
A21: The number of patients suffering from non-communicable chronic diseases	31	1	19%	81%	26	1	58%	42%$
A22: The number of patients suffering from communicable diseases (separately: prevalence & incidence)	31	0	19%	81%	26	2	58%	42%$
A23: The number of patients suffering from communicable diseases and in need of a continuum of care	31	2	19%	81%	26	2	63%	38%$
A24: The number of patients reached through community-based mental health services	31	1	26%	74%	26	3	52%	48%$
A25: The number of individuals/children excluded from the incentivization scheme of preventive physical activities	31	5	48%	52%$	-	-	-	-
A26: The number of parents of young children had a discussion on immunization with their doctors (in the case of voluntary vaccination policy)	31	3	52%	48%$	-	-	-	-
A27: The number of institutions incentivized by the government to initiate preventive physical activities at the micro level	31	3	44%	56%$	-	-	-	-
A28: The number of patients who avail continuum of care for communicable diseases	31	4	33%	67%$	-	-	-	-
A29: The number of individuals reimbursed through their health insurance for preventative physical activities	31	2	46%	54%$	-	-	-	-
A30: The number of children fully immunized (in the case of mandatory vaccination policy)	31	0	19%	81%	26	2	38%	63%$
A30N: The share of all insured people listed in a digital vaccination register by population group	1	-	-	-+	26	1	48%	52%$
A31: The number of children/individuals who have a vaccination card (needed in the case of an emergency outbreak where authority can verify the vaccination status)	31	1	22%	78%	26	2	42%	58%$
A32: The percentage of health promotion programs with communication plans clearly stated	31	2	35%	65%$	-	-	-	-
A33: The percentage of health promotion programs with measurable outputs and outcomes and a proper registry system	31	1	22%	78%	26	1	56%	44%$
A34: The number of schools/workplaces/municipalities made physical activities mandatory for children/employees/residents of different age groups	31	3	32%	68%$	-	-	-	-
**Indicators on other topics**								
A35: The degree of change of specific health indicators as an outcome measure of the impact of digital tools	31	0	26%	74%	26	2	21%	79%
A36: The number of hard-copy processes	31	7	62%	38%$	-	-	-	-
A36N: The number of hard-copy processes in contrast to digital processes in the healthcare system	1	-	-	-+	26	1	36%	64%$
A37: The percentage of a process run-through accomplished with the tool	31	12	25%	75%	26	6	45%	55%$
A38: The number of skilled professionals in Business Intelligence tools	31	5	36%	64%$	-	-	-	-
A39: The number of active Business Intelligence reports	31	7	48%	52%$	-	-	-	-
A40: The average rating of a digital health service in a relevant rating portal	1	-	-	-+	26	5	21%	79%

Supplementary Table 5. Digital public health tools

	Round 2				Round 3			
**Digital alternatives to traditional health services**	**n**	**Could not rate**	**Rank 1-2***	**Rank 3-4***	**n**	**Could not rate**	**Rank 1-2***	**Rank 3-4***
T01: Electronic referral	31	2	10%	90%	26	1	12%	88%
T02: Electronic prescription	31	0	10%	90%	26	1	8%	92%
T03: Electronic patient folder	31	1	10%	90%	26	2	13%	88%
T04: Electronic registries (e.g., for vaccination)	31	0	3%	97%	26	2	0%	100%
T05: Electronic medical records	31	0	13%	87%	26	1	4%	96%
T06: The use of Business Intelligence for monitoring, audit & data exploration	31	4	22%	78%	26	2	13%	88%
T07: Telelectronic health/telemedicine/telecare	31	0	16%	84%	26	0	12%	88%
T08: Electronic health record	31	0	3%	97%	26	0	4%	96%
T09: Digital Health ID (each person can claim 1 unique ID)	31	0	10%	90%	26	1	12%	88%
T10: Social media chat with health promotion professionals/health care providers	31	0	42%	58%[$]	-	-	-	-
T11: Video consultation	31	1	23%	77%	26	0	23%	77%
T12: Digital twin' of a patient	31	13	67%	33%[$]	-	-	-	-
T13: Electronic health insurance card	31	2	41%	59%[$]	-	-	-	-
T14: Decision support systems	31	0	19%	81%	26	0	27%	73%
T15: The use of Business Intelligence for communication and collaboration	31	3	39%	61%[$]	-	-	-	-
T16: Surveillance tools	31	1	7%	93%	26	0	15%	85%
T17: Digital chronic disease management tools	1	-	-	-[+]	26	1	16%	84%
T18: Digital screening programs	1	-	-	-[+]	26	1	20%	80%
T19: Smart care homes and residences	1	-	-	-[+]	26	1	32%	68%[$]
**Mobile health tools**								
T20: Wearables	31	0	23%	77%	26	0	35%	65%[$]
T21: Sensors	31	0	23%	77%	26	0	35%	65%[$]
T22: Mobile tools for data collection	31	0	10%	90%	26	0	12%	88%
T23: Smartphone & web health or medical apps	31	1	10%	90%	26	0	15%	85%
T24: Tracking devices (e.g., physical activity, blood pressure)	31	1	17%	83%	26	0	12%	88%
**Information and education services**								
T25: Dashboards	31	4	4%	96%	26	3	9%	91%
T26: Data visualization tools	31	2	3%	97%	26	0	27%	73%
T27: Health information websites by public health authorities/institutions	31	0	6%	94%	26	0	8%	92%
T28: Healthcare alert systems	31	1	10%	90%	26	2	25%	75%

Information and education services	Round 2 n	Round 2 Could not rate	Round 2 Rank 1-2*	Round 2 Rank 3-4*	Round 3 n	Round 3 Could not rate	Round 3 Rank 1-2*	Round 3 Rank 3-4*
T28N: Digital safety alarms	1	-	-	-+	26	2	13%	83%
T29: Serious games for training purposes	31	1	53%	47%$	-	-	-	-
T30: School curricula on digital public health	31	1	33%	67%$	-	-	-	-
T31: Digital health education materials	1	-	-	-+	26	0	23%	77%
**Infrastructure service**								
T32: Digital progress hubs	31	7	38%	63%$	-	-	-	-
T33: National health data repositories	31	0	10%	90%	26	2	21%	79%
T34: Telematikinfrastructure (so that data can be used beyond interfaces on an outpatient and clinical basis)	31	4	33%	67%$	-	-	-	-
T35: Solutions for the transfer of digital measures to regional care that optimize practice	31	4	11%	89%	26	5	33%	67%$
T36: Risk models and algorithms	31	0	16%	84%	26	3	32%	68%$
T37: Real-world evidence generation and follow-up agencies	1	-	-	-+	26	2	29%	71%

*The calculation includes only those experts, who voted on the Likert scale unimportant (1), barely important (2), somewhat important (3), and very important (4). Those that selected the “I can’t rate this indicator due to a lack of expertise” were excluded from the consensus calculation.

+Indicators or DiPH tools that were first given (as alternative formulations) during the second panel round.

$Indicators or DiPH tools with less than 70% votes for “somewhat important” or “very important”, which were, therefore, excluded.

Interventions with a share of less than 70% for “somewhat important” or “very important” votes were excluded for the next panel round.
Regarding alternative formulations of indicators, only the phrasing with the highest share of “somewhat important” or “very important” votes were kept.

Supplementary File 2: Sub-group and sensitivity analysis

We assumed that different understandings of usable and practical indicators could arise based on national regulations or the scientific background of participating experts. Therefore, we conducted a sub-group analysis for each indicator and digital public health (DiPH) tool to see if the share of "somewhat important" and "very important" replies differed between the two over-represented sub-groups (participants from Germany, experts with a public health background, and Experts from Europe) and the rest of the participating experts. A conflict was defined as one sub-group with at least 70% agreement on keeping an indicator (70% of the participants choosing "somewhat important" or "very important") while the other group had a lower overall rating. The results of this analysis are displayed below.

The sub-group analysis for experts in public health versus participants with a different scientific background produced a similar number of conflicts: 9/30 *ICT* indicators, 6/34 *Legal* indicators, 14/41 *Social* indicators, 14/38 *Application* indicators, and 5/32 *DiPH tools* differed in their overall decision whether to include an indicator or not. For the third round, the overall conflicts increased in total: 20/33 *ICT* indicators, 12/38 *Legal* indicators, 14/35 *Social* indicators, 13/33 *Application* indicators, and 6/30 conflicts in *DiPH tools* were identified for the final panel. The differences per indicator are displayed in Supplementary Tables 7 to 11.

For the sub-group analysis, occupation in Germany versus employment in another country, 9/30 *ICT* indicators, 8/34 *Legal* indicators, 11/41 *Social* indicators, 10/38 *Application* indicators, and 4/32 *DiPH tools* caused conflicts during the second round. During the third round, 11/33 *Information-Communication-Technology* (*ICT)* indicators, 12/38 *Legal* indicators, 9/35 *Social* indicators, 11/33 *Application* indicators, and 6/30 *DiPH tools* had differences between overall sub-group agreement share. For more details, see Supplementary Tables 12 to 16.

The analysis for occupation in Europe compared to experts working in other countries, we observed 13/30 *ICT* indicators, 8/34 *Legal* indicators, 15/41 *Social* indicators, 15/38 *Application* indicators, and 8/32 conflicting *DiPH* tools rating in the second Delphi panel. For the third panel, conflicts occurred for 10/33 *ICT* indicators, 8/38 *Legal* indicators, 8/35 *Social* indicators, 9/33 *Application* indicators, and 6/30 *DiPH* tools. For more details, see Supplementary Tables 17 to 21.

Based on these findings, we conducted a sensitivity analysis to assess if the overall share of "somewhat important" or "very important" ratings for each of the five sub-sections was significantly biased by decisions from the sub-groups. After testing for normal distribution through the Kolmogorov-Smirnov test, we conducted a two-sided Gaussian test with alpha 5%

(normal distribution was given for all four dimensions and DiPH tools). The p-values and the sub-group sizes per domain are displayed in Supplementary Table 6.

Supplementary Table 6. Significance in differences for ratings among sub-groups

	Round 2			Round 3		
	Public Health	***Germany***	***Europe***	***Public Health***	***Germany***	***Europe***
ICT	$p = 0,47$ (n = 7 vs 13)	$p = 0,42$ (n = 8 vs 12)	$p < 0,02$* (n = 17 vs 3)	$p < 0,01$* (n = 3 vs 12)	$p = 0,14$ (n = 6 vs 9)	$p = 0,26$ (n = 13 vs 2)
Legal	$p = 0,41$ (n = 6 vs 16)	$p = 0,70$ (n = 7 vs 15)	$p = 0,37$ (n = 15 vs 7)	$p = 0,12$ (n = 5 vs 12)	$p = 0,66$ (n = 5 vs 12)	$p = 0,52$ (n = 12 vs 5)
Social	$p = 0,58$ (n = 8 vs 11)	$p = 0,06$ (n = 5 vs 14)	$p = 0,13$ (n = 16 vs 3)	$p = 0,99$ (n = 6 vs 10)	$p = 0,26$ (n = 4 vs 12)	$p = 0,31$ (n = 11 vs 5)
Application	$p = 0,17$ (n = 14 vs 14)	$p = 0,65$ (n = 7 vs 21)	$p = 0,11$ (n = 22 vs 6)	$p < 0,01$* (n = 11 vs 15)	$p = 0,01$* (n = 8 vs 18)	$p = 0,24$ (n = 20 vs 6)
Tool	$p = 0,23$ (n = 16 vs 15)	$p = 0,55$ (n = 8 vs 23)	$p = 0,03$* (n = 24 vs 7)	$p = 0,02$* (n = 11 vs 15)	$p = 0,38$ (n = 8 vs 18)	$p = 0,16$ (n = 20 vs 6)

* Statistically significant difference between groups with 95% CI

Supplementary Table 7. Differences by scientific background: Public Health versus other. % of "somewhat important" and "very important" ratings: ICT

	Round 2			Round 3		
**Indicators on expenses, investment and workforce**	**% Public Health (n=7)**	**% Other (n=13)**	**% Total (n=20)**	**% Public Health (n=3)**	**% Other (n=12)**	**% Total (n=15)**
I01: The ratio between private health expenditure and household disposable income health expenditure	67%	50%	56%[$]	-	-	-
I02: The average prices for fixed broadband Internet connections per month	100%	62%	75%	33%	83%	73%
I03: The average prices for pre-paid contracts for mobile phones per month	100%	54%	70%	67%	17%	60%[$]
I04: The annual telecommunications revenue (% of gross domestic product)	29%	33%	32%[$]	-	-	-
I05: The annual telecommunications investment (% of revenue)	57%	58%	58%[$]	-	-	-
I06: The percentage of health expenditure in gross domestic product (public and private)	100%	58%	74%	100%	55%	64%[$]
I07: The annual spending by the government on the information-communication-infrastructure	86%	77%	80%	67%	92%	87%
I08: The level of feedback and communication with various publics	14%	67%	47%[$]	-	-	-
I09: The level of education	83%	92%	89%	0%	83%	67%[$]
I09N: The level of training in the use and billing of digital public health services by health care professionals	-[+]	-[+]	-[+]	100%	83%	87%
I10: The number of feedbacks from patients	83%	69%	95%	0%	75%	64%[$]
I11: The number of skilled professionals in data science	100%	92%	95%	67%	92%	87%
I12: The proportion of the total business sector workforce involved in the information-telecommunication-technology sector	57%	58%	58%[$]	-	-	-
I13: The number of participants in training programs for using digital public health tools by groups (e.g., healthcare workers, patients, or family caregivers)	86%	85%	85%	33%	64%	57%[$]
I13N1: The average number of participants in digital public health tool training programs in a year by different target groups	-[+]	-[+]	-[+]	100%	91%	93%
I13N2: The average number of digital public health tool training provided to different target groups	-[+]	-[+]	-[+]	33%	91%	79%
**Indicators on the availability of the Internet and Internet-enabled devices such as PCs, phones & tablets**						
I14: The share of households/hospitals/physician offices connected to the Internet (by bandwidth)	100%	92%	95%	100%	92%	93%
I15: The personal computers per 100 people	57%	58%	58%[$]	-	-	-
I16: The secure Internet servers (per 1 million people)	86%	45%	61%[$]	-	-	-
I17: The share of households, hospitals & physician offices with a computer	86%	100%	95%	0%	91%	71%
I17N: The share of specific locations that are equipped with at least one Internet-connected device by location (e.g., households, hospitals or outpatient physician's offices)	-[+]	-[+]	-[+]	100%	100%	100%
I18: The number of Internet-connected devices per 100 people by the device (e.g., computers, smartphones, tablets, or notebooks)	-[+]	-[+]	-[+]	67%	75%	73%
I19: The percentage of the overall population with access to the Internet by speed	-[+]	-[+]	-[+]	100%	92%	93%

Indicators on the availability of the Internet and Internet-enabled devices such as PCs, phones & tablets	Round 2 % Public Health (n=7)	Round 2 % Other (n=13)	Round 2 % Total (n=20)	Round 3 % Public Health (n=3)	Round 3 % Other (n=12)	Round 3 % Total (n=15)
I20: The percentage of the overall population with access to computers	-+	-+	-+	67%	75%	73%
I21: The percentage of the population covered by at least a 3G mobile network	86%	75%	79%	100%	83%	87%
I22: The telephone lines per 100 people	43%	25%	32%$	-	-	-
I23: The percentage of the overall population with access to smartphones	-+	-+	-+	100%	100%	100%
**Indicators on interoperability and infrastructure**						
I24: The share or paperless hospitals of all hospitals, when delivering and managing patient care	86%	92%	89%	0%	100%	79%
I24N: The percentage of all hospitals that use electronic documentation systems for patient care	-	-	-	100%	100%	100%
I25: The availability of health information exchange platforms	100%	100%	100%	67%	100%	93%
I26: The existence of a decentralized infrastructure that leaves the data where they originated & accessible based on consent and ID approval	86%	92%	89%	33%	82%	71%
I26N1: The existence of a decentralized data infrastructure	-+	-+	-+	33%	70%	62%$
I26N2: The existence of the possibility to access personal health data based on consent and ID approval	-+	-+	-+	100%	100%	100%
I27: The degree of existing telematic infrastructure	83%	83%	83%	100%	92%	93%
I28: The degree of interoperability of health systems	86%	100%	95%	100%	100%	100%
I29: The degree of compliance of specific interventions with the data exchange and interoperability standards	86%	100%	95%	67%	100%	92%
I30: The existence of interoperable data end-to-end encryption	67%	91%	82%	33%	100%	85%
I31: The number of integrated digital systems in the healthcare system	-+	-+	-+	64%	79%	72%
**Indicators on other topics**						
I32: The percentage of operators with a waiting time less than the guaranteed maximum response time	80%	75%	76%	0%	78%	64%$
I33: The number of adverse events	83%	67%	73%	0%	82%	69%$
I33N: The number of technical malfunctions per digital public health tool reported per year	-+	-+	-+	50%	92%	86%
I34: The percentage of users with first hospital specialty appointments performed within the reference time	40%	70%	60%$	-	-	-
I35: The degree of internationalization	80%	42%	53%$	-	-	-

*The calculation includes only those experts, who voted on the Likert scale unimportant (1), barely important (2), somewhat important (3), and very important (4). Those that selected the “I can’t rate this indicator due to a lack of expertise” were excluded from the consensus calculation.
+Indicators or DiPH tools that were first given (as alternative formulations) during the second panel round.
$Indicators or DiPH tools with less than 70% votes for “somewhat important” or “very important”, which were, therefore, excluded.

Interventions with a share of less than 70% for “somewhat important” or “very important” votes were excluded for the next panel round.
Regarding alternative formulations of indicators, only the phrasing with the highest share of “somewhat important” or “very important” votes were kept.

Supplementary Table 8. Differences by scientific background: Public Health versus other. % of "somewhat important" and "very important" ratings: Legal

Indicators on health data (access, exchange, security)	Round 2			Round 3		
	% Public Health (n=6)	% Other (n=16)	% Total (n=22)	% Public Health (n=5)	% Other (n=12)	% Total (n=17)
L01: The percentage of user consent to health data	50%	94%	85%	40%	91%	75%
L02: The coverage of international standards in stored data	80%	77%	78%	20%	100%	76%
L03: The existence of a legal framework for exchanging health data digitally between different stakeholders	100%	100%	100%	100%	100%	100%
L04: The degree of political support in data transfer & exchange	100%	75%	81%	100%	67%	76%
L05: The existence of a legal framework for the secondary use of health data	100%	88%	90%	100%	92%	94%
L06: The existence of regulations for access to health data through electronic health records	100%	94%	95%	100%	100%	100%
L07: The existence of legislation regulating the interaction between the digital data of a patient's health and the data of bioinformatics / genetic information of the biomaterials of this patient	-[+]	-[+]	-[+]	60%	89%	79%
L08: The level of encryption of personal and health data	75%	94%	90%	80%	83%	82%
L09: The ratio of encrypted records to the total number of records	75%	73%	74%	40%	82%	69%[$]
L10: The existence of regulations that the digital data collected via electronic health applications are only stored on one's own electronic health card	100%	69%	76%	80%	60%	67%[$]
L11: The coverage of metadata labels in sensitive data	75%	92%	88%	25%	100%	79%
L12: The number of critical failures in security points	75%	100%	95%	80%	91%	88%
L13: The number of weaknesses in security perimeters	75%	93%	89%	80%	89%	86%
L14: The level of data security	100%	100%	100%	80%	90%	87%
**Indicators on digital assets**						
L15: The existence of a policy or legislative reform to allow live-time digital asset	67%	85%	81%	80%	55%	62%[$]
L16: The existence of policy/legislative reform to allow access to all digital assets that would fit into the World Health Organizations' definition of health and may be considered non-health assets (education, Social system, work, income, etc.) traditionally	25%	86%	72%	60%	45%	50%[$]
L17: The existence of a policy or legislative reform to allow access to digital assets	75%	85%	82%	80%	73%	75%
**Indicators on the digital public health strategy**						
L18: The existence of political briefs/policies that mention digital health	60%	69%	67%[$]	-	-	-
L19: The existence of a political strategy to digitalize the healthcare system	100%	93%	95%	80%	92%	88%
L20: The existence of a digital public health strategy within the governmental health strategy	100%	87%	90%	80%	92%	88%
L21: The existence of guidelines for planning & implementing digital public health tools	80%	81%	81%	100%	92%	94%
L22: The level of harmonization of legal regulations in electronic health	100%	94%	95%	60%	67%	65%[$]
L23: The existence of legal supervision of the implementation of national digital public health programs	100%	75%	81%	60%	100%	88%
L24: The existence of a department for digital health in the health ministry	80%	81%	81%	80%	82%	81%
L25: The existence of regulation on the function of electronic health products (e.g., Medical Devices Act)	50%	88%	80%	100%	91%	94%

Indicators on the digital public health strategy	Round 2			Round 3		
	% Public Health (n=6)	% Other (n=16)	% Total (n=22)	% Public Health (n=5)	% Other (n=12)	% Total (n=17)
L26: The existence of a legal right for citizens to be provided with digital health services	-+	-+	-+	80%	70%	73%
L27: The existence of a digital public health policy engaged with the protection of fundamental rights of vulnerable groups (e.g., children, adolescents, mentally disabled people)	-+	-+	-+	80%	75%	76%
L28: The existence of a policy to promote innovation and the development of digital tools in the public health system	-+	-+	-+	80%	83%	82%
**Indicators on finances and reimbursement**						
L29: The existence of a public funding scheme for digital public health interventions on a regional and national level	100%	63%	70%	80%	83%	82%
L30: The existence of investment and reimbursement possibilities from the government	75%	80%	79%	100%	83%	88%
L31: The annual spending by the government as support for the implementation of digital technologies in healthcare	100%	81%	85%	80%	73%	75%
L32: The number of digital applications remunerated	25%	50%	44%$	-	-	-
L33: The existence of financial incentives for health professionals to participate in offering digital public health services	75%	75%	75%	80%	83%	82%
**Indicators on other topics**						
L34: The availability of legal tools, such as contracts templates	25%	67%	58%$	-	-	-
L35: The rate of Direct Public Offering (DPO) approval and other activities	0%	22%	18%$	-	-	-
L36: The existence of a law/regulation on prevention on the regional/national level	60%	63%	62%$	-	-	-
L37: The efficiency of the alarm system at all levels of healthcare	67%	54%	56%$	-	-	-
L38: The number of civic bodies that have the autonomy to organize the health care services at least at the primary and secondary levels of care (decentralization component)	33%	31%	32%$	-	-	-
L39: The existence of an action plan to prevent discrimination based on race, gender, sexual orientation & age in digital health services	-+	-+	-+	60%	64%	63%$
L40: The existence of a statutory provision that places restrictions and quarantine periods on public health leadership professionals for future private digital health activities after they have ceased their previous public service activities	-+	-+	-+	60%	30%	40%$
L41: The existence of legal liability for public health managers for digital health contracts that harm patients or the public interest	-+	-+	-+	80%	70%	73%
L42: The existence of policy standards for transparency and the protection of fundamental rights in using artificial intelligence in digital public health	-+	-+	-+	100%	92%	94%
L43: The level of transparency and accessibility of digital health contracts to public oversight and law enforcement agencies for anti-corruption purposes	-+	-+	-+	60%	75%	71%
L44: The effectiveness of informed patient consent for using personal health data in adult patients	-+	-+	-+	60%	83%	76%
L45: The existence of unique procedures to protect children, adolescents and mentally ill patients who are unable to give their consent	-+	-+	-+	60%	83%	76%

Supplementary Table 9. Differences by scientific background: Public Health versus other. % of "somewhat important" and "very important" ratings: Social

Indicators on potential and actual users	Round 2			Round 3		
	% Public Health (n=8)	% Other (n=11)	% Total (n=19)	% Public Health (n=6)	% Other (n=10)	% Total (n=16)
S01: The number of people that are willing to use a digital public health tool or participate in a digital public health intervention	100%	91%	95%	100%	80%	88%
S02: The number of potential users of the same digital public health tool	100%	64%	79%	100%	80%	87%
S03: The number of potential users of a digital public health tool who have adequate access to the web	100%	80%	89%	83%	80%	81%
S04: The share of the eligible population who have used at least one digital public health intervention for routine care and health promotion in the previous year	63%	82%	74%	67%	100%	88%
S05: The share of the eligible population who have signed up (i.e., have an account) for at least one digital public health intervention focused on routine care and health promotion	50%	82%	68%$	-	-	-
S06: The number of patients using apps to interact with local services	88%	82%	84%	83%	90%	88%
S07: The number of digital contacts	75%	56%	65%$	-	-	-
S08: The adherence of users to the intervention (in percentage)	75%	67%	71%	67%	78%	73%
S09: The ratio of electronic health records to the total number of user records	71%	89%	81%	83%	67%	73%
S10: The number of patients enrolled after communication campaigns	38%	82%	63%$	-	-	-
S11: The number of patients enrolled in app use	88%	64%	74%	33%	40%	38%$
S11N1: The number of patients enrolled in the use of specific health apps	-+	-+	-+	50%	90%	75%
S11N2: The share of the population that uses any health or medical app by reason (e.g., health promotion, wellness, tracing)	-+	-+	-+	67%	90%	81%
S12: The number of digital public health professionals	-+	-+	-+	55%	93%	76%
S13: The number of patients interacting with apps	88%	73%	68%$	-	-	-
S14: The number of digital platforms used and views reach and interaction	50%	73%	63%$	-	-	-
S15: The number of posts placed on digital platforms	25%	36%	32%$	-	-	-
S16: The utilization rate of specific groups per digital public health intervention (by health insurance claims data)	88%	89%	88%	80%	44%	57%$
**Indicators on awareness, trust & motivation**						
S17: The perceived usefulness of specific digital public health tools by groups on a Likert scale	100%	56%	76%	100%	63%	79%
S18: The share of the population that trusts digital health services	75%	100%	89%	100%	80%	88%
S19: The motivation to access electronic health services by groups of people	71%	82%	78%	67%	78%	73%
S20: The awareness of health professionals about the value of data and the possibility of their use by information-telecommunication-technology	100%	82%	89%	100%	80%	88%
S21: The awareness of groups of people that the intervention exists (by target groups)	100%	64%	79%	100%	90%	94%
S22: The level of trust in the government by different groups of the population	-+	-+	-+	83%	50%	63%$
S23: The satisfaction rate	80%	69%	74%	50%	56%	53%$
S23N1: The self-reported satisfaction rate with digital public health intervention by user group	-+	-+	-+	83%	70%	75%
S23N2: The share of the population that is more satisfied with using the digital public health intervention compared to standard care	-+	-+	-+	67%	90%	81%

Indicators on literacy	Round 2 % Public Health (n=8)	Round 2 % Other (n=11)	Round 2 % Total (n=19)	Round 3 % Public Health (n=6)	Round 3 % Other (n=10)	Round 3 % Total (n=16)
S24: The average level of digital literacy by different target groups on a Likert Scale	100%	82%	89%	100%	89%	93%
S25: The average level of digital health literacy by different target groups on a Likert Scale	100%	90%	94%	100%	78%	87%
S26: The average level of health literacy by different target groups on a Likert Scale	88%	67%	76%	83%	89%	87%
S27: The average level of information-telecommunication-technology skills by groups of people	100%	82%	89%	83%	90%	88%
**Indicators on use of smart- and cellular phones**						
S28: The share of the population that uses a smartphone by different target groups	100%	91%	95%	83%	80%	81%
S29: The share of the population that owns a smartphone by different target groups	100%	73%	84%	83%	70%	75%
S30: The smartphone penetration rate versus the use of mobile devices for routine care and health promotion in the population	100%	92%	95%	67%	92%	87%
S31: The mobile cellular subscriptions per 100 people	63%	30%	44%[$]	-	-	-
S32: The proportion of healthcare users using smartphones and other digital devices	88%	80%	83%	83%	80%	81%
**Indicators on the use of the Internet**						
S33: The active mobile-broadband subscriptions per 100 inhabitants	71%	77%	75%	100%	90%	92%
S34: The fixed broadband Internet subscriptions per 100 inhabitants	75%	64%	74%	33%	92%	80%
S35: The percentage of Internet users in the country	100%	73%	95%	100%	83%	87%
S36: The share of the population that uses the Internet for gathering health information by different target groups	75%	100%	89%	83%	80%	81%
S37: The number of searches for specific digital public health interventions measured on Google Trends	-[+]	-[+]	-[+]	67%	80%	75%
**Indicators on other topics**						
S38: The number of various health promotion campaigns led by students/youth	38%	45%	42%[$]	-	-	-
S39: The number of professional bodies consulted for patient-provider interactions regarding the service of routine care	25%	60%	44%[$]	-	-	-
S40: The number of patients contacted to obtain feedback on routine care for chronic diseases	50%	70%	61%[$]	-	-	-
S41: The degree of impact of communication campaigns	43%	50%	47%[$]	-	-	-
S42: The number of disease-specific patient groups consulted for discussing care and treatment modalities in chronic diseases	71%	73%	72%	60%	63%	62%[$]
S43: The number of prospective parents reached out for counseling on child immunization	43%	45%	44%[$]	-	-	-
S44: The number of institutions (schools/workplaces and others) that develop mechanisms through the consultative process (with children/employees) for promoting/adopting the government-sponsored/facilitated preventive physical activities	57%	60%	59%[$]	-	-	-

Supplementary Table 10. Differences by scientific background: Public Health versus other. % of "somewhat important" and "very important" ratings: Application

Indicators on access to the service and information	Round 2			Round 3		
	% Public Health (n=14)	% Other (n=14)	% Total (n=28)	% Public Health (n=11)	% Other (n=15)	% Total (n=26)
A01: The number of patients reached through telemedicine	46%	86%	67%$	-	-	-
A02: The availability of digital information on public health interventions as national prevention programs	92%	79%	85%	55%	93%	77%
A02N1: The share of available digital information on public health interventions	-+	-+	-+	45%	87%	69%$
A02N2: The availability of reliable information on specific digital public health services	-+	-+	-+	91%	93%	92%
A03: The number of individuals reached through digital public health system (via mobile messaging) for health promotion information/communication	69%	64%	67%$	-	-	-
A04: The percentage of the overall population with access	91%	91%	92%	30%	93%	68%$
A04N: The percentage of the overall population with access to the digital public health tool	-+	-+	-+	100%	100%	100%
A05: The availability of reliable health information in a digital format	75%	88%	85%	73%	93%	84%
A06: The proportion of persons who cannot access their digital data and the provision of alternative access	-+	-+	-+	91%	87%	88%
**Indicators on secondary use of health data**						
A07: The share of patient health data used for monitoring or evaluating the healthcare system	77%	92%	85%	73%	71%	72%
A07N1: The share of patient health data used for evaluating healthcare services	-+	-+	-+	64%	80%	73%
A07N2: The share of population health data used for public health monitoring	-+	-+	-+	55%	87%	73%
A08: The percentage run-throughs completed (re)use of data	63%	55%	58%$	-	-	-
A09: The degree of data interoperability	100%	92%	96%	60%	86%	75%
A09N: The degree of technical/syntactic/semantic interoperability	-+	-+	-+	100%	86%	91%
A10: The degree of data linkage	92%	85%	88%	55%	86%	72%
A10N: The availability of a unique identifier to link health data for a person between different digital public health tools/platforms	-+	-+	-+	100%	93%	96%
A11: The degree of data accessibility	100%	86%	92%	91%	100%	96%
**Indicators on service implementation**						
A12: The degree to which an intervention is established (e.g., local pilot, communal, regional, national)	100%	73%	87%	82%	73%	77%
A12N: The level to which an intervention is established (e.g., local pilot, communal, regional, national)	-+	-+	-+	60%	62%	61%$
A13: The degree to which the intervention complements existing workflows or duplicates them	77%	73%	75%	22%	69%	50%$
A13N: The extent of redundancy in workflows created by introducing digital public health tools/interventions to complement existing workflows	-+	-+	-+	82%	69%	75%
A14: The percentage of health promotion programs integrated into national health system digital platforms	62%	71%	67%$	-	-	-
A15: The proportion of (specific) public health services delivered via digital technologies (may be disaggregated by technology type)	69%	62%	65%$	-	-	-

Indicators on service implementation	Round 2			Round 3		
	% Public Health (n=14)	% Other (n=14)	% Total (n=28)	% Public Health (n=11)	% Other (n=15)	% Total (n=26)
A16: The proportion of digital public health interventions considering health equity in their planning, implementation & evaluation	83%	86%	85%	91%	93%	92%
**Indicators on access to the service and information**						
A17: The proportion of public health services implementing at least one digital technology intervention	46%	50%	48%$	-	-	-
A18: The number of regulated digital health services included in routine care	69%	86%	78%	60%	64%	63%$
A19: The total number of records	25%	71%	61%$	-	-	-
**Indicators on traditional health services and prevention**						
A20: The number of patients recommended by their treating physician to adopt regular physical activities (such as joining fitness club/swimming)	46%	71%	59%$	-	-	-
A21: The number of patients suffering from non-communicable chronic diseases	69%	93%	81%	36%	46%	42%$
A22: The number of patients suffering from communicable diseases (separately: prevalence & incidence)	77%	86%	81%	36%	46%	42%$
A23: The number of patients suffering from communicable diseases and in need of a continuum of care	75%	86%	81%	30%	43%	38%$
A24: The number of patients reached through community-based mental health services	62%	86%	74%	50%	46%	48%$
A25: The number of individuals/children excluded from the incentivization scheme of preventive physical activities	50%	54%	52%$	-	-	-
A26: The number of parents of young children had a discussion on immunization with their doctors (in the case of voluntary vaccination policy)	50%	46%	48%$	-	-	-
A27: The number of institutions incentivized by the government to initiate preventive physical activities at the micro level	42%	69%	56%$	-	-	-
A28: The number of patients who avail continuum of care for communicable diseases	58%	75%	67%$	-	-	-
A29: The number of individuals reimbursed through their health insurance for preventative physical activities	50%	57%	54%$	-	-	-
A30: The number of children fully immunized (in the case of mandatory vaccination policy)	69%	93%	81%	36%	85%	63%$
A30N: The share of all insured people listed in a digital vaccination register by population group	-+	-+	-+	55%	50%	52%$
A31: The number of children/individuals who have a vaccination card (needed in the case of an emergency outbreak where authority can verify the vaccination status)	77%	79%	78%	40%	71%	58%$
A32: The percentage of health promotion programs with communication plans clearly stated	50%	79%	65%$	-	-	-
A33: The percentage of health promotion programs with measurable outputs and outcomes and a proper registry system	77%	79%	78%	27%	57%	44%$
A34: The number of schools/workplaces/municipalities made physical activities mandatory for children/employees/residents of different age groups	58%	77%	68%$	-	-	-
A35: The degree of change of specific health indicators as an outcome measure of the impact of digital tools	88%	64%	74%	100%	67%	79%
A36: The number of hard-copy processes	40%	36%	38%$	-	-	-
A36N: The number of hard-copy processes in contrast to digital processes in the healthcare system	-+	-+	-+	64%	64%	64%$
A37: The percentage of a process run-through accomplished with the tool	63%	88%	75%	25%	75%	55%$
A38: The number of skilled professionals in Business Intelligence tools	80%	50%	64%$	-	-	-

Indicators on traditional health services and prevention	Round 2 % Public Health (n=14)	Round 2 % Other (n=14)	Round 2 % Total (n=28)	Round 3 % Public Health (n=11)	Round 3 % Other (n=15)	Round 3 % Total (n=26)
A39: The number of active Business Intelligence reports	70%	36%	52%$	-	-	-
A40: The average rating of a digital health service in a relevant rating portal	-+	-+	-+	50%	83%	79%

*The calculation includes only those experts, who voted on the Likert scale unimportant (1), barely important (2), somewhat important (3), and very important (4). Those that selected the "I can't rate this indicator due to a lack of expertise" were excluded from the consensus calculation.
+Indicators or DiPH tools that were first given (as alternative formulations) during the second panel round.
$Indicators or DiPH tools with less than 70% votes for "somewhat important" or "very important", which were, therefore, excluded.

Interventions with a share of less than 70% for "somewhat important" or "very important" votes were excluded for the next panel round.
Regarding alternative formulations of indicators, only the phrasing with the highest share of "somewhat important" or "very important" votes were kept.

Supplementary Table 11. Differences by scientific background: Public Health versus other. % of "somewhat important" and "very important" ratings: DiPH Tools

Indicators on access to the service and information	Round 2			Round 3		
	% Public Health (n=16)	% Other (n=15)	% Total (n=31)	% Public Health (n=11)	% Other (n=15)	% Total (n=26)
T01: Electronic referral	87%	93%	90%	73%	100%	88%
T02: Electronic prescription	88%	93%	90%	82%	100%	92%
T03: Electronic patient folder	93%	87%	90%	82%	92%	88%
T04: Electronic registries (e.g., for vaccination)	100%	93%	97%	100%	100%	100%
T05: Electronic medical records	88%	87%	87%	91%	100%	96%
T06: The use of Business Intelligence for monitoring, audit & data exploration	77%	79%	78%	90%	86%	88%
T07: Telelectronic health/telemedicine/telecare	75%	93%	84%	82%	93%	88%
T08: Electronic health record	94%	100%	97%	91%	100%	96%
T09: Digital Health ID (each person can claim 1 unique ID)	94%	87%	90%	80%	93%	88%
T10: Social media chat with health promotion professionals/health care providers	56%	60%	58%[$]	-	-	-
T11: Video consultation	69%	86%	77%	64%	87%	77%
T12: Digital twin' of a patient	14%	45%	33%[$]	-	-	-
T13: Electronic health insurance card	60%	57%	59%[$]	-	-	-
T14: Decision support systems	69%	93%	81%	64%	80%	73%
T15: The use of Business Intelligence for communication and collaboration	50%	71%	61%[$]	-	-	-
T16: Surveillance tools	88%	100%	93%	91%	80%	85%
T17: Digital chronic disease management tools	-[+]	-[+]	-[+]	82%	86%	84%
T18: Digital screening programs	-[+]	-[+]	-[+]	73%	86%	80%
T19: Smart care homes and residences	-[+]	-[+]	-[+]	50%	80%	68%[$]
**Mobile health tools**						
T20: Wearables	75%	80%	77%	73%	60%	65%[$]
T21: Sensors	69%	87%	77%	64%	67%	65%[$]
T22: Mobile tools for data collection	94%	87%	90%	91%	87%	88%
T23: Smartphone & web health or medical apps	88%	93%	90%	73%	93%	85%
T24: Tracking devices (e.g., physical activity, blood pressure)	80%	87%	83%	82%	93%	88%
**Information and education services**						
T25: Dashboards	100%	93%	96%	100%	83%	91%
T26: Data visualization tools	93%	100%	97%	73%	73%	73%
T27: Health information websites by public health authorities/institutions	88%	100%	94%	91%	93%	92%
T28: Healthcare alert systems	93%	87%	90%	73%	77%	75%

Indicators on access to the service and information	Round 2 % Public Health (n=16)	Round 2 % Other (n=15)	Round 2 % Total (n=31)	Round 3 % Public Health (n=11)	Round 3 % Other (n=15)	Round 3 % Total (n=26)
T28N: Digital safety alarms	-+	-+	-+	82%	85%	83%
T29: Serious games for training purposes	40%	53%	47%$	-	-	-
T30: School curricula on digital public health	60%	73%	67%$	-	-	-
T31: Digital health education materials	-+	-+	-+	73%	80%	77%
**Infrastructure service**						
T32: Digital progress hubs	58%	67%	63%$	-	-	-
T33: National health data repositories	100%	80%	90%	70%	86%	79%
T34: Telematikinfrastructure (so that data can be used beyond interfaces on an outpatient and clinical basis)	67%	67%	67%$	-	-	-
T35: Solutions for the transfer of digital measures to regional care that optimize practice	83%	93%	89%	67%	67%	67%$
T36: Risk models and algorithms	83%	93%	84%	70%	67%	68%$
T37: Real-world evidence generation and follow-up agencies	-+	-+	-+	83%	69%	71%

*The calculation includes only those experts, who voted on the Likert scale unimportant (1), barely important (2), somewhat important (3), and very important (4). Those that selected the “I can’t rate this indicator due to a lack of expertise” were excluded from the consensus calculation.
+Indicators or DiPH tools that were first given (as alternative formulations) during the second panel round.
$Indicators or DiPH tools with less than 70% votes for “somewhat important” or “very important”, which were, therefore, excluded.

Interventions with a share of less than 70% for “somewhat important” or “very important” votes were excluded for the next panel round.
Regarding alternative formulations of indicators, only the phrasing with the highest share of “somewhat important” or “very important” votes were kept.

Supplementary Table 12. Differences by countries: Germany versus other. % of "somewhat important" and "very important" ratings: ICT

Indicators on access to the service and information	Round 2			Round 3		
	% Germany (n=8)	% Other (n=12)	% Total (n=20)	% Germany (n=6)	% Other (n=9)	% Total (n=15)
I01: The ratio between private health expenditure and household disposable income health expenditure	57%	55%	56%$	-	-	-
I02: The average prices for fixed broadband Internet connections per month	75%	75%	75%	100%	56%	73%
I03: The average prices for pre-paid contracts for mobile phones per month	88%	58%	70%	83%	44%	60%$
I04: The annual telecommunications revenue (% of gross domestic product)	14%	42%	32%$	-	-	-
I05: The annual telecommunications investment (% of revenue)	71%	50%	58%$	-	-	-
I06: The percentage of health expenditure in gross domestic product (public and private)	43%	92%	74%	50%	75%	64%$
I07: The annual spending by the government on the information-communication-infrastructure	88%	75%	80%	100%	78%	87%
I08: The level of feedback and communication with various publics	43%	50%	47%$	-	-	-
I09: The level of education	86%	91%	89%	67%	67%	67%$
I09N: The level of training in the use and billing of digital public health services by health care professionals	-+	-+	-+	83%	89%	87%
I10: The number of feedbacks from patients	63%	82%	95%	67%	63%	64%$
I11: The number of skilled professionals in data science	100%	91%	95%	100%	78%	87%
I12: The proportion of the total business sector workforce involved in the information-telecommunication-technology sector	43%	67%	58%$	-	-	-
I13: The number of participants in training programs for using digital public health tools by groups (e.g., healthcare workers, patients, or family caregivers)	100%	75%	85%	17%	88%	57%$
I13N1: The average number of participants in digital public health tool training programs in a year by different target groups	-+	-+	-+	83%	100%	93%
I13N2: The average number of digital public health tool training provided to different target groups	-+	-+	-+	83%	75%	79%
**Indicators on the availability of the Internet and Internet-enabled devices such as PCs, phones & tablets**						
I14: The share of households/hospitals/physician offices connected to the Internet (by bandwidth)	100%	92%	95%	83%	100%	93%
I15: The personal computers per 100 people	71%	50%	58%$	-	-	-
I16: The secure Internet servers (per 1 million people)	71%	55%	61%$	-	-	-
I17: The share of households, hospitals & physician offices with a computer	100%	92%	95%	100%	75%	71%
I17N: The share of specific locations that are equipped with at least one Internet-connected device by location (e.g., households, hospitals or outpatient physician's offices)	-+	-+	-+	100%	100%	100%
I18: The number of Internet-connected devices per 100 people by the device (e.g., computers, smartphones, tablets, or notebooks)	-+	-+	-+	67%	78%	73%
I19: The percentage of the overall population with access to the Internet by speed	-+	-+	-+	83%	100%	93%
I20: The percentage of the overall population with access to computers	-+	-+	-+	67%	78%	73%
I21: The percentage of the population covered by at least a 3G mobile network	86%	75%	79%	83%	89%	87%
I22: The telephone lines per 100 people	29%	33%	32%$	-	-	-
I23: The percentage of the overall population with access to smartphones	-+	-+	-+	100%	100%	100%

Indicators on interoperability and infrastructure	Round 2 % Germany (n=8)	Round 2 % Other (n=12)	Round 2 % Total (n=20)	Round 3 % Germany (n=6)	Round 3 % Other (n=9)	Round 3 % Total (n=15)
I24: The share or paperless hospitals of all hospitals, when delivering and managing patient care	88%	91%	89%	83%	75%	79%
I24N: The percentage of all hospitals that use electronic documentation systems for patient care	-	-	-	100%	100%	100%
I25: The availability of health information exchange platforms	100%	100%	100%	100%	89%	93%
I26: The existence of a decentralized infrastructure that leaves the data where they originated & accessible based on consent and ID approval	88%	91%	89%	67%	75%	71%
I26N1: The existence of a decentralized data infrastructure	-+	-+	-+	50%	71%	62%$
I26N2: The existence of the possibility to access personal health data based on consent and ID approval	-+	-+	-+	100%	100%	100%
I27: The degree of existing telematic infrastructure	100%	70%	83%	83%	100%	93%
I28: The degree of interoperability of health systems	100%	91%	95%	100%	100%	100%
I29: The degree of compliance of specific interventions with the data exchange and interoperability standards	88%	100%	95%	80%	100%	92%
I30: The existence of interoperable data end-to-end encryption	86%	80%	82%	80%	88%	85%
I31: The number of integrated digital systems in the healthcare system	-+	-+	-+	75%	71%	72%
**Indicators on other topics**						
I32: The percentage of operators with a waiting time less than the guaranteed maximum response time	43%	100%	76%	40%	83%	64%$
I33: The number of adverse events	50%	89%	73%	50%	86%	69%$
I33N: The number of technical malfunctions per digital public health tool reported per year	-+	-+	-+	83%	88%	86%
I34: The percentage of users with first hospital specialty appointments performed within the reference time	17%	89%	60%$	-	-	-
I35: The degree of internationalization	38%	67%	53%$	-	-	-

*The calculation includes only those experts, who voted on the Likert scale unimportant (1), barely important (2), somewhat important (3), and very important (4). Those that selected the "I can't rate this indicator due to a lack of expertise" were excluded from the consensus calculation.
+Indicators or DiPH tools that were first given (as alternative formulations) during the second panel round.
$Indicators or DiPH tools with less than 70% votes for "somewhat important" or "very important", which were, therefore, excluded.

Interventions with a share of less than 70% for "somewhat important" or "very important" votes were excluded for the next panel round.
Regarding alternative formulations of indicators, only the phrasing with the highest share of "somewhat important" or "very important" votes were kept.

Supplementary Table 13. Differences by countries: Germany versus other. % of "somewhat important" and "very important" ratings: Legal

Indicators on health data (access, exchange, security)	Round 2			Round 3		
	% Germany (n=7)	% Other (n=15)	% Total (n=22)	% Germany (n=5)	% Other (n=12)	% Total (n=17)
L01: The percentage of user consent to health data	86%	85%	85%	60%	82%	75%
L02: The coverage of international standards in stored data	75%	79%	78%	80%	75%	76%
L03: The existence of a legal framework for exchanging health data digitally between different stakeholders	100%	100%	100%	100%	100%	100%
L04: The degree of political support in data transfer & exchange	43%	100%	81%	80%	75%	76%
L05: The existence of a legal framework for the secondary use of health data	100%	86%	90%	100%	92%	94%
L06: The existence of regulations for access to health data through electronic health records	100%	93%	95%	100%	100%	100%
L07: The existence of legislation regulating the interaction between the digital data of a patient's health and the data of bioinformatics / genetic information of the biomaterials of this patient	-[+]	-[+]	-[+]	50%	90%	79%
L08: The level of encryption of personal and health data	86%	92%	90%	80%	83%	82%
L09: The ratio of encrypted records to the total number of records	83%	69%	74%	60%	73%	69%[$]
L10: The existence of regulations that the digital data collected via electronic health applications are only stored on one's own electronic health card	83%	73%	76%	100%	55%	67%[$]
L11: The coverage of metadata labels in sensitive data	80%	91%	88%	75%	80%	79%
L12: The number of critical failures in security points	83%	100%	95%	75%	92%	88%
L13: The number of weaknesses in security perimeters	83%	92%	89%	67%	91%	86%
L14: The level of data security	100%	100%	100%	75%	91%	87%
**Indicators on digital assets**						
L15: The existence of a policy or legislative reform to allow live-time digital asset	80%	82%	81%	25%	75%	62%[$]
L16: The existence of policy/legislative reform to allow access to all digital assets that would fit into the World Health Organizations' definition of health and may be considered non-health assets traditionally	67%	75%	72%	25%	58%	50%[$]
L17: The existence of a policy or legislative reform to allow access to digital assets	75%	85%	82%	50%	83%	75%
**Indicators on the digital public health strategy**						
L18: The existence of political briefs/policies that mention digital health	71%	64%	67%[$]	-	-	-
L19: The existence of a political strategy to digitalize the healthcare system	83%	100%	95%	80%	92%	88%
L20: The existence of a digital public health strategy within the governmental health strategy	83%	93%	90%	80%	92%	88%
L21: The existence of guidelines for planning & implementing digital public health tools	86%	79%	81%	100%	92%	94%
L22: The level of harmonization of legal regulations in electronic health	100%	93%	95%	80%	58%	65%[$]
L23: The existence of legal supervision of the implementation of national digital public health programs	86%	79%	81%	100%	83%	88%
L24: The existence of a department for digital health in the health ministry	86%	79%	81%	100%	73%	81%
L25: The existence of regulation on the function of electronic health products (e.g., Medical Devices Act)	100%	69%	80%	100%	91%	94%

Indicators on the digital public health strategy	Round 2 % Germany (n=7)	Round 2 % Other (n=15)	Round 2 % Total (n=22)	Round 3 % Germany (n=5)	Round 3 % Other (n=12)	Round 3 % Total (n=17)
L26: The existence of a legal right for citizens to be provided with digital health services	-[+]	-[+]	-[+]	100%	64%	73%
L27: The existence of a digital public health policy engaged with the protection of fundamental rights of vulnerable groups (e.g., children, adolescents, mentally disabled people)	-[+]	-[+]	-[+]	80%	75%	76%
L28: The existence of a policy to promote innovation and the development of digital tools in the public health system	-[+]	-[+]	-[+]	100%	75%	82%
**Indicators on finances and reimbursement**						
L29: The existence of a public funding scheme for digital public health interventions on a regional/national level	57%	77%	70%	100%	75%	82%
L30: The existence of investment and reimbursement possibilities from the government	86%	75%	79%	80%	92%	88%
L31: The annual spending by the government as support for the implementation of digital technologies in healthcare	100%	77%	85%	80%	73%	75%
L32: The number of digital applications remunerated	50%	42%	44%[$]	-	-	-
L33: The existence of financial incentives for health professionals to participate in offering digital public health services	71%	77%	75%	80%	83%	82%
**Indicators on other topics**						
L34: The availability of legal tools, such as contracts templates	71%	50%	58%[$]	-	-	-
L35: The rate of Direct Public Offering (DPO) approval and other activities	0%	25%	18%[$]	-	-	-
L36: The existence of a law/regulation on prevention on the regional/national level	43%	71%	62%[$]	-	-	-
L37: The efficiency of the alarm system at all levels of healthcare	50%	60%	56%[$]	-	-	-
L38: The number of civic bodies that have the autonomy to organize the health care services at least at the primary and secondary levels of care (decentralization component)	29%	33%	32%[$]	-	-	-
L39: The existence of an action plan to prevent discrimination based on race, gender, sexual orientation & age in digital health services	-[+]	-[+]	-[+]	60%	64%	63%[$]
L40: The existence of a statutory provision that places restrictions and quarantine periods on public health leadership professionals for future private digital health activities after they have ceased their previous public service activities	-[+]	-[+]	-[+]	25%	45%	40%[$]
L41: The existence of legal liability for public health managers for digital health contracts that harm patients or the public interest	-[+]	-[+]	-[+]	67%	75%	73%
L42: The existence of policy standards for transparency and the protection of fundamental rights in using artificial intelligence in digital public health	-[+]	-[+]	-[+]	100%	92%	94%
L43: The level of transparency and accessibility of digital health contracts to public oversight and law enforcement agencies for anti-corruption purposes	-[+]	-[+]	-[+]	60%	75%	71%
L44: The effectiveness of informed patient consent for using personal health data in adult patients	-[+]	-[+]	-[+]	80%	75%	76%
L45: The existence of unique procedures to protect children, adolescents and mentally ill patients who are unable to give their consent	-[+]	-[+]	-[+]	82%	67%	76%

Supplementary Table 14. Differences by countries: Germany versus other. % of “somewhat important” and “very important” ratings: Social

Indicators on the digital public health strategy	Round 2 % Germany (n=5)	Round 2 % Other (n=14)	Round 2 % Total (n=19)	Round 3 % Germany (n=4)	Round 3 % Other (n=12)	Round 3 % Total (n=16)
S01: The number of people that are willing to use a digital public health tool or participate in a digital public health intervention	100%	93%	95%	100%	83%	88%
S02: The number of potential users of the same digital public health tool	40%	93%	79%	75%	91%	87%
S03: The number of potential users of a digital public health tool who have adequate access to the web	75%	93%	89%	75%	83%	81%
S04: The share of the eligible population who have used at least one digital public health intervention for routine care and health promotion in the previous year	80%	71%	74%	100%	83%	88%
S05: The share of the eligible population who have signed up (i.e., have an account) for at least one digital public health intervention focused on routine care and health promotion	80%	64%	68%$	-	-	-
S06: The number of patients using apps to interact with local services	80%	86%	84%	75%	92%	88%
S07: The number of digital contacts	33%	71%	65%$	-	-	-
S08: The adherence of users to the intervention (in percentage)	50%	77%	71%	50%	82%	73%
S09: The ratio of electronic health records to the total number of user records	67%	85%	81%	33%	83%	73%
S10: The number of patients enrolled after communication campaigns	60%	64%	63%$	-	-	-
S11: The number of patients enrolled in app use	80%	71%	74%	0%	50%	38%$
S11N1: The number of patients enrolled in the use of specific health apps	-+	-+	-+	75%	75%	75%
S11N2: The share of the population that uses any health or medical app by reason (e.g., health promotion, wellness, tracing)	-+	-+	-+	75%	83%	81%
S12: The number of digital public health professionals	-+	-+	-+	88%	71%	76%
S13: The number of patients interacting with apps	80%	79%	68%$	-	-	-
S14: The number of digital platforms used and views reach and interaction	40%	71%	63%$	-	-	-
S15: The number of posts placed on digital platforms	0%	43%	32%$	-	-	-
S16: The utilization rate of specific groups per digital public health intervention (by health insurance claims data)	75%	92%	88%	75%	50%	57%$
**Indicators on awareness, trust & motivation**						
S17: The perceived usefulness of specific digital public health tools by groups on a Likert scale	75%	77%	76%	50%	90%	79%
S18: The share of the population that trusts digital health services	100%	86%	89%	75%	92%	88%
S19: The motivation to access electronic health services by groups of people	80%	77%	78%	67%	75%	73%
S20: The awareness of health professionals about the value of data and the possibility of their use by information-telecommunication-technology	80%	93%	89%	75%	92%	88%
S21: The awareness of groups of people that the intervention exists (by target groups)	80%	79%	79%	100%	92%	94%
S22: The level of trust in the government by different groups of the population	-+	-+	-+	50%	67%	63%$
S23: The satisfaction rate	67%	76%	74%	25%	64%	53%$

Indicators on awareness, trust & motivation	Round 2			Round 3		
	% Germany (n=5)	% Other (n=14)	% Total (n=19)	% Germany (n=4)	% Other (n=12)	% Total (n=16)
S23N1: The self-reported satisfaction rate with digital public health intervention by user group	-[+]	-[+]	-[+]	50%	83%	75%
S23N2: The share of the population that is more satisfied with using the digital public health intervention compared to standard care	-[+]	-[+]	-[+]	75%	83%	81%
**Indicators on literacy**						
S24: The average level of digital literacy by different target groups on a Likert Scale	100%	86%	89%	100%	91%	93%
S25: The average level of digital health literacy by different target groups on a Likert Scale	100%	92%	94%	75%	91%	87%
S26: The average level of health literacy by different target groups on a Likert Scale	75%	77%	76%	100%	82%	87%
S27: The average level of information-telecommunication-technology skills by groups of people	100%	85%	89%	83%	90%	88%
**Indicators on use of smart- and cellular phones**						
S28: The share of the population that uses a smartphone by different target groups	100%	93%	95%	100%	75%	81%
S29: The share of the population that owns a smartphone by different target groups	60%	93%	84%	75%	75%	75%
S30: The smartphone penetration rate versus the use of mobile devices for routine care and health promotion in the population	88%	100%	95%	83%	89%	87%
S31: The mobile cellular subscriptions per 100 people	50%	43%	44%[$]	-	-	-
S32: The proportion of healthcare users using smartphones and other digital devices	80%	85%	83%	75%	83%	81%
**Indicators on use of the Internet**						
S33: The active mobile-broadband subscriptions per 100 inhabitants	75%	75%	75%	83%	100%	92%
S34: The fixed broadband Internet subscriptions per 100 inhabitants	86%	67%	74%	100%	67%	80%
S35: The percentage of Internet users in the country	100%	92%	95%	83%	89%	87%
S36: The share of the population that uses the Internet for gathering health information by different target groups	100%	86%	89%	100%	75%	81%
S37: The number of searches for specific digital public health interventions measured on Google Trends	-[+]	-[+]	-[+]	100%	67%	75%
**Indicators on other topics**						
S38: The number of various health promotion campaigns led by students/youth	20%	50%	42%[$]	-	-	-
S39: The number of professional bodies consulted for patient-provider interactions regarding the service of routine care	25%	50%	44%[$]	-	-	-
S40: The number of patients contacted to obtain feedback on routine care for chronic diseases	50%	64%	61%[$]	-	-	-
S41: The degree of impact of communication campaigns	60%	42%	47%[$]	-	-	-
S42: The number of disease-specific patient groups consulted for discussing care and treatment modalities in chronic diseases	60%	77%	72%	33%	70%	62%[$]
S43: The number of prospective parents reached out for counseling on child immunization	20%	54%	44%[$]	-	-	-
S44: The number of institutions (schools/workplaces and others) that develop mechanisms through the consultative process (with children/employees) for promoting/adopting the government-sponsored/facilitated preventive physical activities	25%	69%	59%[$]	-	-	-

Supplementary Table 15. Differences by countries: Germany versus other. % of "somewhat important" and "very important" ratings: Application

Indicators on access to the service and information	Round 2			Round 3		
	% Germany (n=7)	% Other (n=21)	% Total (n=28)	% Germany (n=8)	% Other (n=18)	% Total (n=26)
A01: The number of patients reached through telemedicine	50%	71%	67%[$]	-	-	-
A02: The availability of digital information on public health interventions as national prevention programs	100%	81%	85%	75%	78%	77%
A02N1: The share of available digital information on public health interventions	-[+]	-[+]	-[+]	63%	72%	69%[$]
A02N2: The availability of reliable information on specific digital public health services	-[+]	-[+]	-[+]	88%	94%	92%
A03: The number of individuals reached through digital public health system (via mobile messaging) for health promotion information/communication	83%	62%	67%[$]	-	-	-
A04: The percentage of the overall population with access	83%	94%	92%	57%	72%	68%[$]
A04N: The percentage of the overall population with access to the digital public health tool	-[+]	-[+]	-[+]	100%	100%	100%
A05: The availability of reliable health information in a digital format	71%	92%	85%	75%	88%	84%
A06: The proportion of persons who cannot access their digital data and the provision of alternative access	-[+]	-[+]	-[+]	88%	89%	88%
**Indicators on secondary use of health data**						
A07: The share of patient health data used for monitoring or evaluating the healthcare system	100%	80%	85%	88%	65%	72%
A07N1: The share of patient health data used for evaluating healthcare services	-[+]	-[+]	-[+]	63%	78%	73%
A07N2: The share of population health data used for public health monitoring	-[+]	-[+]	-[+]	75%	72%	73%
A08: The percentage run-throughs completed (re)use of data	25%	67%	58%[$]	-	-	-
A09: The degree of data interoperability	100%	95%	96%	57%	82%	75%
A09N: The degree of technical/syntactic/semantic interoperability	-[+]	-[+]	-[+]	100%	88%	91%
A10: The degree of data linkage	83%	89%	88%	43%	83%	72%
A10N: The availability of a unique identifier to link health data for a person between different digital public health tools/platforms	-[+]	-[+]	-[+]	100%	94%	96%
A11: The degree of data accessibility	83%	95%	92%	88%	100%	96%
**Indicators on service implementation**						
A12: The degree to which an intervention is established (e.g., local pilot, communal, regional, national)	100%	82%	87%	75%	78%	77%
A12N: The level to which an intervention is established (e.g., local pilot, communal, regional, national)	-[+]	-[+]	-[+]	43%	69%	61%[$]
A13: The degree to which the intervention complements existing workflows or duplicates them	83%	72%	75%	50%	50%	50%[$]
A13N: The extent of redundancy in workflows created by introducing digital public health tools/interventions to complement existing workflows	-[+]	-[+]	-[+]	71%	76%	75%
A14: The percentage of health promotion programs integrated into national health system digital platforms	83%	62%	67%[$]	-	-	-
A15: The proportion of (specific) public health services delivered via digital technologies (may be disaggregated by technology type)	83%	60%	65%[$]	-	-	-

Indicators on service implementation	Round 2 % Germany (n=7)	Round 2 % Other (n=21)	Round 2 % Total (n=28)	Round 3 % Germany (n=8)	Round 3 % Other (n=18)	Round 3 % Total (n=26)
A16: The proportion of digital public health interventions considering health equity in their planning, implementation & evaluation	83%	85%	85%	75%	100%	92%
A17: The proportion of public health services implementing at least one digital technology intervention	67%	42%	48%$	-	-	-
A18: The number of regulated digital health services included in routine care	100%	71%	78%	80%	55%	63%
A19: The total number of records	83%	50%	61%$	-	-	-
**Indicators on traditional health services and prevention**						
A20: The number of patients recommended by their treating physician to adopt regular physical activities (such as joining fitness club/swimming)	67%	57%	59%$	-	-	-
A21: The number of patients suffering from non-communicable chronic diseases	83%	81%	81%	25%	50%	42%$
A22: The number of patients suffering from communicable diseases (separately: prevalence & incidence)	83%	81%	81%	25%	50%	42%$
A23: The number of patients suffering from communicable diseases and in need of a continuum of care	83%	80%	81%	25%	44%	38%$
A24: The number of patients reached through community-based mental health services	83%	71%	74%	14%	63%	48%$
A25: The number of individuals/children excluded from the incentivization scheme of preventive physical activities	33%	59%	52%$	-	-	-
A26: The number of parents of young children had a discussion on immunization with their doctors (in the case of voluntary vaccination policy)	33%	53%	48%$	-	-	-
A27: The number of institutions incentivized by the government to initiate preventive physical activities at the micro level	50%	58%	56%$	-	-	-
A28: The number of patients who avail continuum of care for communicable diseases	50%	72%	67%$	-	-	-
A29: The number of individuals reimbursed through their health insurance for preventative physical activities	33%	60%	54%$	-	-	-
A30: The number of children fully immunized (in the case of mandatory vaccination policy)	83%	81%	81%	29%	76%	63%$
A30N: The share of all insured people listed in a digital vaccination register by population group	-+	-+	-+	75%	41%	52%$
A31: The number of children/individuals who have a vaccination card (needed in the case of an emergency outbreak where authority can verify the vaccination status)	83%	76%	78%	43%	65%	58%$
A32: The percentage of health promotion programs with communication plans clearly stated	67%	65%	65%$	-	-	-
A33: The percentage of health promotion programs with measurable outputs and outcomes and a proper registry system	83%	76%	78%	38%	47%	44%$
A34: The number of schools/workplaces/municipalities made physical activities mandatory for children/employees/residents of different age groups	83%	63%	68%$	-	-	-
**Indicators on other topics**						
A35: The degree of change of specific health indicators as an outcome measure of the impact of digital tools	60%	79%	74%	75%	80%	79%
A36: The number of hard-copy processes	17%	47%	38%$	-	-	-

Indicators on other topics	Round 2			Round 3		
	% Germany (n=7)	% Other (n=21)	% Total (n=28)	% Germany (n=8)	% Other (n=18)	% Total (n=26)
A36N: The number of hard-copy processes in contrast to digital processes in the healthcare system	-+	-+	-+	38%	76%	64%$
A37: The percentage of a process run-through accomplished with the tool	50%	83%	75%	50%	57%	55%$
A38: The number of skilled professionals in Business Intelligence tools	20%	76%	64%$	-	-	-
A39: The number of active Business Intelligence reports	40%	56%	52%$	-	-	-
A40: The average rating of a digital health service in a relevant rating portal	-+	-+	-+	50%	100%	79%

*The calculation includes only those experts, who voted on the Likert scale unimportant (1), barely important (2), somewhat important (3), and very important (4). Those that selected the “I can’t rate this indicator due to a lack of expertise” were excluded from the consensus calculation.

+Indicators or DiPH tools that were first given (as alternative formulations) during the second panel round.

$Indicators or DiPH tools with less than 70% votes for “somewhat important” or “very important”, which were, therefore, excluded.

Interventions with a share of less than 70% for “somewhat important” or “very important” votes were excluded for the next panel round.
Regarding alternative formulations of indicators, only the phrasing with the highest share of “somewhat important” or “very important” votes were kept.

Supplementary Table 16. Differences by countries: Germany versus other. % of "somewhat important" and "very important" ratings: DiPH Tools

	Round 2			Round 3		
**Digital alternatives to traditional health services**	**% Germany (n=8)**	**% Other (n=23)**	**% Total (n=31)**	**% Germany (n=8)**	**% Other (n=18)**	**% Total (n=26)**
T01: Electronic referral	86%	91%	90%	86%	89%	88%
T02: Electronic prescription	88%	91%	90%	86%	94%	92%
T03: Electronic patient folder	100%	86%	90%	71%	94%	88%
T04: Electronic registries (e.g., for vaccination)	100%	96%	97%	100%	100%	100%
T05: Electronic medical records	100%	83%	87%	100%	94%	96%
T06: The use of Business Intelligence for monitoring, audit & data exploration	67%	81%	78%	67%	94%	88%
T07: Telelectronic health/telemedicine/telecare	88%	83%	84%	88%	89%	88%
T08: Electronic health record	100%	96%	97%	100%	94%	96%
T09: Digital Health ID (each person can claim 1 unique ID)	75%	96%	90%	88%	88%	88%
T10: Social media chat with health promotion professionals/health care providers	63%	57%	58%[$]	-	-	-
T11: Video consultation	88%	73%	77%	88%	72%	77%
T12: Digital twin' of a patient	40%	31%	33%[$]	-	-	-
T13: Electronic health insurance card	50%	62%	59%[$]	-	-	-
T14: Decision support systems	63%	87%	81%	75%	72%	73%
T15: The use of Business Intelligence for communication and collaboration	57%	62%	61%[$]	-	-	-
T16: Surveillance tools	100%	91%	93%	88%	83%	85%
T17: Digital chronic disease management tools	-[+]	-[+]	-[+]	100%	76%	84%
T18: Digital screening programs	-[+]	-[+]	-[+]	100%	71%	80%
T19: Smart care homes and residences	-[+]	-[+]	-[+]	63%	71%	68%[$]
**Mobile health tools**						
T20: Wearables	88%	74%	77%	75%	61%	65%[$]
T21: Sensors	88%	74%	77%	63%	67%	65%[$]
T22: Mobile tools for data collection	88%	91%	90%	88%	89%	88%
T23: Smartphone & web health or medical apps	75%	95%	90%	75%	89%	85%
T24: Tracking devices (e.g., physical activity, blood pressure)	75%	86%	83%	88%	89%	88%
**Information and education services**						
T25: Dashboards	100%	95%	96%	100%	88%	91%
T26: Data visualization tools	86%	100%	97%	88%	67%	73%
T27: Health information websites by public health authorities/institutions	100%	91%	94%	88%	94%	92%
T28: Healthcare alert systems	88%	91%	90%	88%	72%	75%

Infrastructure services	Round 2 % Germany (n=8)	Round 2 % Other (n=23)	Round 2 % Total (n=31)	Round 3 % Germany (n=8)	Round 3 % Other (n=18)	Round 3 % Total (n=26)
T28N: Digital safety alarms	-+	-+	-+	100%	76%	83%
T29: Serious games for training purposes	43%	48%	47%$	-	-	-
T30: School curricula on digital public health	63%	68%	67%$	-	-	-
T31: Digital health education materials	-+	-+	-+	88%	72%	77%
T32: Digital progress hubs	50%	67%	63%$	-	-	-
T33: National health data repositories	75%	96%	90%	71%	82%	79%
T34: Telematikinfrastructure (so that data can be used beyond interfaces on an outpatient and clinical basis)	75%	63%	67%$	-	-	-
T35: Solutions for the transfer of digital measures to regional care that optimize practice	88%	89%	89%	80%	63%	67%$
T36: Risk models and algorithms	63%	91%	84%	71%	67%	68%$
T37: Real-world evidence generation and follow-up agencies	-+	-+	-+	71%	71%	71%

*The calculation includes only those experts, who voted on the Likert scale unimportant (1), barely important (2), somewhat important (3), and very important (4). Those that selected the "I can't rate this indicator due to a lack of expertise" were excluded from the consensus calculation.
+Indicators or DiPH tools that were first given (as alternative formulations) during the second panel round.
$Indicators or DiPH tools with less than 70% votes for "somewhat important" or "very important", which were, therefore, excluded.

Interventions with a share of less than 70% for "somewhat important" or "very important" votes were excluded for the next panel round.
Regarding alternative formulations of indicators, only the phrasing with the highest share of "somewhat important" or "very important" votes were kept.

Supplementary Table 17. Differences by continents: Europe versus other. % of "somewhat important" and "very important ratings": ICT

	Round 2			Round 3		
**Indicators on expenses, investment and workforce**	**% Europe (n=17)**	**% Other (n=3)**	**% Total (n=20)**	**% Europe (n=13)**	**% Other (n=2)**	**% Total (n=15)**
I01: The ratio between private health expenditure and household disposable income health expenditure	56%	50%	56%$	-	-	-
I02: The average prices for fixed broadband Internet connections per month	76%	67%	75%	77%	50%	73%
I03: The average prices for pre-paid contracts for mobile phones per month	71%	67%	70%	69%	0%	60%$
I04: The annual telecommunications revenue (% of gross domestic product)	25%	67%	32%$	-	-	-
I05: The annual telecommunications investment (% of revenue)	50%	100%	58%$	-	-	-
I06: The percentage of health expenditure in gross domestic product (public and private)	69%	100%	74%	67%	50%	64%$
I07: The annual spending by the government on the information-communication-infrastructure	76%	100%	80%	92%	50%	87%
I08: The level of feedback and communication with various publics	44%	67%	47%$	-	-	-
I09: The level of education	87%	100%	89%	69%	50%	67%$
I09N: The level of training in the use and billing of digital public health services by health care professionals	-+	-+	-+	92%	50%	87%
I10: The number of feedbacks from patients	69%	100%	95%	67%	50%	64%$
I11: The number of skilled professionals in data science	94%	100%	95%	85%	100%	87%
I12: The proportion of the total business sector workforce involved in the information-telecommunication-technology sector	56%	67%	58%$	-	-	-
I13: The number of participants in training programs for using digital public health tools by groups (e.g., healthcare workers, patients, or family caregivers)	88%	67%	85%	54%	100%	57%$
I13N1: The average number of participants in digital public health tool training programs in a year by different target groups	-+	-+	-+	92%	100%	93%
I13N2: The average number of digital public health tool training provided to different target groups	-+	-+	-+	77%	100%	79%
**Indicators on the availability of the Internet and Internet-enabled devices such as PCs, phones & tablets**						
I14: The share of households/hospitals/physician offices connected to the Internet (by bandwidth)	94%	100%	95%	92%	100%	92%
I15: The personal computers per 100 people	56%	67%	58%$	-	-	-
I16: The secure Internet servers (per 1 million people)	63%	50%	61%$	-	-	-
I17: The share of households, hospitals & physician offices with a computer	100%	67%	95%	69%	100%	71%
I17N: The share of specific locations that are equipped with at least one Internet-connected device by location (e.g., households, hospitals or outpatient physician's offices)	-+	-+	-+	100%	100%	100%
I18: The number of Internet-connected devices per 100 people by the device (e.g., computers, smartphones, tablets, or notebooks)	-+	-+	-+	69%	100%	73%
I19: The percentage of the overall population with access to the Internet by speed	-+	-+	-+	92%	100%	93%
I20: The percentage of the overall population with access to computers	-+	-+	-+	77%	50%	73%

Indicators on the availability of the Internet and Internet-enabled devices such as PCs, phones & tablets	Round 2 % Europe (n=17)	Round 2 % Other (n=3)	Round 2 % Total (n=20)	Round 3 % Europe (n=13)	Round 3 % Other (n=2)	Round 3 % Total (n=15)
I21: The percentage of the population covered by at least a 3G mobile network	81%	67%	79%	85%	100%	87%
I22: The telephone lines per 100 people	31%	33%	32%$	-	-	-
I23: The percentage of the overall population with access to smartphones	-+	-+	-+	100%	100%	100%
**Indicators on interoperability and infrastructure**						
I24: The share or paperless hospitals of all hospitals, when delivering and managing patient care	88%	100%	89%	77%	100%	79%
I24N: The percentage of all hospitals that use electronic documentation systems for patient care	-	-	-	100%	100%	100%
I25: The availability of health information exchange platforms	100%	100%	100%	92%	100%	93%
I26: The existence of a decentralized infrastructure that leaves the data where they originated & accessible based on consent and ID approval	88%	100%	89%	69%	100%	71%
I26N1: The existence of a decentralized data infrastructure	-+	-+	-+	58%	100%	62%$
I26N2: The existence of the possibility to access personal health data based on consent and ID approval	-+	-+	-+	100%	100%	100%
I27: The degree of existing telematic infrastructure	82%	100%	83%	92%	100%	93%
I28: The degree of interoperability of health systems	94%	100%	95%	100%	100%	100%
I29: The degree of compliance of specific interventions with the data exchange and interoperability standards	94%	100%	95%	91%	100%	92%
I30: The existence of interoperable data end-to-end encryption	80%	100%	82%	82%	100%	85%
I31: The number of integrated digital systems in the healthcare system	-+	-+	-+	75%	60%	72%
**Indicators on other topics**						
I32: The percentage of operators with a waiting time less than the guaranteed maximum response time	71%	100%	76%	60%	100%	64%$
I33: The number of adverse events	69%	100%	73%	64%	100%	69%$
I33N: The number of technical malfunctions per digital public health tool reported per year	-+	-+	-+	83%	100%	86%
I34: The percentage of users with first hospital specialty appointments performed within the reference time	58%	67%	60%$	-	-	-
I35: The degree of internationalization	43%	100%	53%$	-	-	-

*The calculation includes only those experts, who voted on the Likert scale unimportant (1), barely important (2), somewhat important (3), and very important (4). Those that selected the “I can’t rate this indicator due to a lack of expertise” were excluded from the consensus calculation.

+Indicators or DiPH tools that were first given (as alternative formulations) during the second panel round.
$Indicators or DiPH tools with less than 70% votes for “somewhat important” or “very important”, which were, therefore, excluded.
Interventions with a share of less than 70% for “somewhat important” or “very important” votes were excluded for the next panel round.
Regarding alternative formulations of indicators, only the phrasing with the highest share of “somewhat important” or “very important” votes were kept.

Supplementary Table 18. Differences by continents: Europe versus other. % of "somewhat important" and "very important ratings": Legal

Indicators on health data (access, exchange, security)	Round 2			Round 3		
	% Europe (n=15)	% Other (n=7)	% Total (n=22)	% Europe (n=12)	% Other (n=5)	% Total (n=17)
L01: The percentage of user consent to health data	86%	83%	85%	75%	75%	75%
L02: The coverage of international standards in stored data	75%	83%	78%	85%	50%	76%
L03: The existence of a legal framework for exchanging health data digitally between different stakeholders	100%	100%	100%	100%	100%	100%
L04: The degree of political support in data transfer & exchange	73%	100%	81%	77%	75%	76%
L05: The existence of a legal framework for the secondary use of health data	93%	83%	90%	92%	100%	94%
L06: The existence of regulations for access to health data through electronic health records	100%	83%	95%	100%	100%	100%
L07: The existence of legislation regulating the interaction between the digital data of a patient's health and the data of bioinformatics / genetic information of the biomaterials of this patient	-[+]	-[+]	-[+]	70%	100%	79%
L08: The level of encryption of personal and health data	86%	100%	90%	77%	100%	82%
L09: The ratio of encrypted records to the total number of records	77%	67%	74%	75%	50%	69%[$]
L10: The existence of regulations that the digital data collected via electronic health applications are only stored on one's own electronic health card	82%	67%	76%	64%	75%	67%[$]
L11: The coverage of metadata labels in sensitive data	90%	83%	88%	82%	67%	79%
L12: The number of critical failures in security points	92%	100%	95%	83%	100%	88%
L13: The number of weaknesses in security perimeters	92%	83%	89%	82%	100%	86%
L14: The level of data security	100%	100%	100%	82%	100%	87%
**Indicators on digital assets**						
L15: The existence of a policy or legislative reform to allow live-time digital asset	82%	80%	81%	58%	75%	62%[$]
L16: The existence of policy/legislative reform to allow access to all digital assets that would fit into the World Health Organizations' definition of health and may be considered non-health assets (education, Social system, work, income, etc.) traditionally	67%	83%	72%	50%	50%	50%[$]
L17: The existence of a policy or legislative reform to allow access to digital assets	73%	100%	82%	75%	75%	75%
**Indicators on the digital public health strategy**						
L18: The existence of political briefs/policies that mention digital health	73%	50%	67%[$]	-	-	-
L19: The existence of a political strategy to digitalize the healthcare system	93%	100%	95%	92%	75%	88%
L20: The existence of a digital public health strategy within the governmental health strategy	86%	100%	90%	92%	75%	88%
L21: The existence of guidelines for planning & implementing digital public health tools	80%	83%	81%	92%	100%	94%
L22: The level of harmonization of legal regulations in electronic health	93%	100%	95%	62%	75%	65%[$]
L23: The existence of legal supervision of the implementation of national digital public health programs	80%	83%	81%	92%	75%	88%
L24: The existence of a department for digital health in the health ministry	73%	100%	81%	83%	75%	81%
L25: The existence of regulation on the function of electronic health products (e.g., Medical Devices Act)	79%	83%	80%	92%	100%	94%

Indicators on health data (access, exchange, security)	Round 2			Round 3		
	% Europe (n=15)	% Other (n=7)	% Total (n=22)	% Europe (n=12)	% Other (n=5)	% Total (n=17)
L26: The existence of a legal right for citizens to be provided with digital health services	-[+]	-[+]	-[+]	73%	75%	73%
L27: The existence of a digital public health policy engaged with the protection of fundamental rights of vulnerable groups (e.g., children, adolescents, mentally disabled people)	-[+]	-[+]	-[+]	77%	75%	76%
L28: The existence of a policy to promote innovation and the development of digital tools in the public health system	-[+]	-[+]	-[+]	85%	75%	82%
**Indicators on finances and reimbursement**						
L29: The existence of a public funding scheme for digital public health interventions on a regional and national level	64%	83%	70%	85%	75%	82%
L30: The existence of investment and reimbursement possibilities from the government	79%	80%	79%	92%	75%	88%
L31: The annual spending by the government as support for the implementation of digital technologies in healthcare	79%	100%	85%	75%	75%	75%
L32: The number of digital applications remunerated	42%	50%	44%[$]	-	-	-
L33: The existence of financial incentives for health professionals to participate in offering digital public health services	79%	67%	75%	85%	75%	82%
**Indicators on other topics**						
L34: The availability of legal tools, such as contracts templates	62%	50%	58%[$]	-	-	-
L35: The rate of Direct Public Offering (DPO) approval and other activities	13%	33%	18%[$]	-	-	-
L36: The existence of a law/regulation on prevention on the regional/national level	57%	71%	62%[$]	-	-	-
L37: The efficiency of the alarm system at all levels of healthcare	50%	75%	56%[$]	-	-	-
L38: The number of civic bodies that have the autonomy to organize the health care services at least at the primary and secondary levels of care (decentralization component)	36%	20%	32%[$]	-	-	-
L39: The existence of an action plan to prevent discrimination based on race, gender, sexual orientation & age in digital health services	-[+]	-[+]	-[+]	67%	50%	63%[$]
L40: The existence of a statutory provision that places restrictions and quarantine periods on public health leadership professionals for future private digital health activities after they have ceased their previous public service activities	-[+]	-[+]	-[+]	36%	50%	40%[$]
L41: The existence of legal liability for public health managers for digital health contracts that harm patients or the public interest	-[+]	-[+]	-[+]	73%	75%	73%
L42: The existence of policy standards for transparency and the protection of fundamental rights in using artificial intelligence in digital public health	-[+]	-[+]	-[+]	92%	100%	94%
L43: The level of transparency and accessibility of digital health contracts to public oversight and law enforcement agencies for anti-corruption purposes	-[+]	-[+]	-[+]	77%	50%	71%
L44: The effectiveness of informed patient consent for using personal health data in adult patients	-[+]	-[+]	-[+]	85%	50%	76%
L45: The existence of unique procedures to protect children, adolescents and mentally ill patients who are unable to give their consent	-[+]	-[+]	-[+]	85%	50%	76%

Supplementary Table 19. Differences by continents: Europe versus other. % of "somewhat important" and "very important ratings": Social

Indicators on potential and actual users	Round 2			Round 3		
	% Europe (n=16)	% Other (n=3)	% Total (n=19)	% Europe (n=11)	% Other (n=5)	% Total (n=16)
S01: The number of people that are willing to use a digital public health tool or participate in a digital public health intervention	94%	100%	95%	91%	80%	88%
S02: The number of potential users of the same digital public health tool	75%	100%	79%	80%	100%	87%
S03: The number of potential users of a digital public health tool who have adequate access to the web	87%	100%	89%	82%	80%	81%
S04: The share of the eligible population who have used at least one digital public health intervention for routine care and health promotion in the previous year	75%	67%	74%	91%	80%	88%
S05: The share of the eligible population who have signed up (i.e., have an account) for at least one digital public health intervention focused on routine care and health promotion	63%	100%	68%$	-	-	-
S06: The number of patients using apps to interact with local services	81%	100%	84%	91%	80%	88%
S07: The number of digital contacts	64%	67%	65%$	-	-	-
S08: The adherence of users to the intervention (in percentage)	71%	67%	71%	70%	80%	73%
S09: The ratio of electronic health records to the total number of user records	86%	50%	81%	70%	80%	73%
S10: The number of patients enrolled after communication campaigns	63%	67%	63%$	-	-	-
S11: The number of patients enrolled in app use	69%	100%	74%	45%	20%	38%$
S11N1: The number of patients enrolled in the use of specific health apps	-+	-+	-+	91%	40%	75%
S11N2: The share of the population that uses any health or medical app by reason (e.g., health promotion, wellness, tracing)	-+	-+	-+	91%	60%	81%
S12: The number of digital public health professionals	-+	-+	-+	90%	20%	76%
S13: The number of patients interacting with apps	69%	100%	68%$	-	-	-
S14: The number of digital platforms used and views reach and interaction	63%	67%	63%$	-	-	-
S15: The number of posts placed on digital platforms	25%	67%	32%$	-	-	-
S16: The utilization rate of specific groups per digital public health intervention (by health insurance claims data)	86%	100%	88%	60%	50%	57%$
**Indicators on awareness, trust & motivation**						
S17: The perceived usefulness of specific digital public health tools by groups on a Likert scale	73%	100%	76%	70%	100%	79%
S18: The share of the population that trusts digital health services	94%	67%	89%	82%	100%	88%
S19: The motivation to access electronic health services by groups of people	87%	33%	78%	70%	80%	73%
S20: The awareness of health professionals about the value of data and the possibility of their use by information-telecommunication-technology	88%	100%	89%	82%	100%	88%
S21: The awareness of groups of people that the intervention exists (by target groups)	75%	100%	79%	91%	100%	94%
S22: The level of trust in the government by different groups of the population	-+	-+	-+	55%	80%	63%$
S23: The satisfaction rate	68%	100%	74%	60%	40%	53%$
S23N1: The self-reported satisfaction rate with digital public health intervention by user group	-+	-+	-+	73%	80%	75%

Indicators on awareness, trust & motivation	Round 2			Round 3		
	% Europe (n=16)	% Other (n=3)	% Total (n=19)	% Europe (n=11)	% Other (n=5)	% Total (n=16)
S23N2: The share of the population that is more satisfied with using the digital public health intervention compared to standard care	-+	-+	-+	82%	80%	81%
**Indicators on literacy**						
S24: The average level of digital literacy by different target groups on a Likert Scale	88%	100%	89%	91%	100%	93%
S25: The average level of digital health literacy by different target groups on a Likert Scale	93%	100%	94%	82%	100%	87%
S26: The average level of health literacy by different target groups on a Likert Scale	73%	100%	76%	91%	75%	87%
S27: The average level of information-telecommunication-technology skills by groups of people	94%	50%	89%	91%	80%	88%
**Indicators on use of smart- and cellular phones**						
S28: The share of the population that uses a smartphone by different target groups	94%	100%	95%	82%	80%	81%
S29: The share of the population that owns a smartphone by different target groups	81%	100%	84%	73%	80%	75%
S30: The smartphone penetration rate versus the use of mobile devices for routine care and health promotion in the population	94%	100%	95%	85%	100%	87%
S31: The mobile cellular subscriptions per 100 people	40%	67%	44%$	-	-	-
S32: The proportion of healthcare users using smartphones and other digital devices	87%	67%	83%	73%	100%	81%
**Indicators on use of the Internet**						
S33: The active mobile-broadband subscriptions per 100 inhabitants	71%	100%	75%	92%	100%	92%
S34: The fixed broadband Internet subscriptions per 100 inhabitants	63%	100%	74%	85%	50%	80%
S35: The percentage of Internet users in the country	94%	100%	95%	85%	100%	87%
S36: The share of the population that uses the Internet for gathering health information by different target groups	94%	67%	89%	91%	60%	81%
S37: The number of searches for specific digital public health interventions measured on Google Trends	-+	-+	-+	91%	40%	75%
**Indicators on other topics**						
S38: The number of various health promotion campaigns led by students/youth	44%	33%	42%$	-	-	-
S39: The number of professional bodies consulted for patient-provider interactions regarding the service of routine care	47%	33%	44%$	-	-	-
S40: The number of patients contacted to obtain feedback on routine care for chronic diseases	60%	67%	61%$	-	-	-
S41: The degree of impact of communication campaigns	50%	33%	47%$	-	-	-
S42: The number of disease-specific patient groups consulted for discussing care and treatment modalities in chronic diseases	67%	100%	72%	56%	75%	62%$
S43: The number of prospective parents reached out for counseling on child immunization	47%	33%	44%$	-	-	-
S44: The number of institutions (schools/workplaces and others) that develop mechanisms through the consultative process (with children/employees) for promoting/adopting the government-sponsored/facilitated preventive physical activities	50%	100%	59%$	-	-	-

Supplementary Table 20. Differences by continents: Europe versus other. % of "somewhat important" and "very important ratings": Application

	Round 2			Round 3		
**Indicators on access to the service and information**	**% Europe (n=22)**	**% Other (n=6)**	**% Total (n=28)**	**% Europe (n=20)**	**% Other (n=6)**	**% Total (n=26)**
A01: The number of patients reached through telemedicine	67%	67%	67%$	-	-	-
A02: The availability of digital information on public health interventions as national prevention programs	86%	83%	85%	76%	80%	77%
A02N1: The share of available digital information on public health interventions	-+	-+	-+	76%	40%	69%$
A02N2: The availability of reliable information on specific digital public health services	-+	-+	-+	90%	100%	92%
A03: The number of individuals reached through digital public health system (via mobile messaging) for health promotion information/communication	62%	83%	67%$	-	-	-
A04: The percentage of the overall population with access	89%	100%	92%	75%	40%	68%$
A04N: The percentage of the overall population with access to the digital public health tool	-	-	-	100%	100%	100%
A05: The availability of reliable health information in a digital format	79%	100%	85%	90%	60%	84%
A06: The proportion of persons who cannot access their digital data and the provision of alternative access	-+	-+	-+	90%	80%	88%
**Indicators on secondary use of health data**						
A07: The share of patient health data used for monitoring or evaluating the healthcare system	90%	67%	85%	75%	60%	72%
A07N1: The share of patient health data used for evaluating healthcare services	-+	-+	-+	76%	60%	73%
A07N2: The share of population health data used for public health monitoring	-+	-+	-+	81%	40%	73%
A08: The percentage run-throughs completed (re)use of data	59%	50%	58%$	-	-	-
A09: The degree of data interoperability	95%	100%	96%	74%	80%	75%
A09N: The degree of technical/syntactic/semantic interoperability	-	-	-	89%	100%	91%
A10: The degree of data linkage	90%	80%	88%	75%	60%	72%
A10N: The availability of a unique identifier to link health data for a person between different digital public health tools/platforms	-+	-+	-+	95%	100%	96%
A11: The degree of data accessibility	90%	100%	92%	95%	100%	96%
**Indicators on service implementation**						
A12: The degree to which an intervention is established (e.g., local pilot, communal, regional, national)	89%	80%	87%	71%	100%	77%
A12N: The level to which an intervention is established (e.g., local pilot, communal, regional, national)	-+	-+	-+	61%	60%	61%$
A13: The degree to which the intervention complements existing workflows or duplicates them	72%	83%	75%	59%	20%	50%$
A13N: The extent of redundancy in workflows created by introducing digital public health tools/interventions to complement existing workflows	-+	-+	-+	74%	80%	75%
A14: The percentage of health promotion programs integrated into national health system digital platforms	71%	50%	67%$	-	-	-
A15: The proportion of (specific) public health services delivered via digital technologies (may be disaggregated by technology type)	65%	67%	65%$	-	-	-

Indicators on service implementation	Round 2 % Europe (n=22)	Round 2 % Other (n=6)	Round 2 % Total (n=28)	Round 3 % Europe (n=20)	Round 3 % Other (n=6)	Round 3 % Total (n=26)
A16: The proportion of digital public health interventions considering health equity in their planning, implementation & evaluation	85%	83%	85%	90%	100%	92%
A17: The proportion of public health services implementing at least one digital technology intervention	47%	50%	48%[$]	-	-	-
A18: The number of regulated digital health services included in routine care	81%	67%	78%	76%	60%	63%
A19: The total number of records	75%	33%	61%[$]	-	-	-
**Indicators on traditional health services and prevention**						
A20: The number of patients recommended by their treating physician to adopt regular physical activities (such as joining fitness club/swimming)	71%	17%	59%[$]	-	-	-
A21: The number of patients suffering from non-communicable chronic diseases	86%	67%	81%	42%	40%	42%[$]
A22: The number of patients suffering from communicable diseases (separately: prevalence & incidence)	81%	83%	81%	42%	40%	42%[$]
A23: The number of patients suffering from communicable diseases and in need of a continuum of care	81%	80%	81%	42%	20%	38%[$]
A24: The number of patients reached through community-based mental health services	81%	50%	74%	50%	40%	48%[$]
A25: The number of individuals/children excluded from the incentivization scheme of preventive physical activities	58%	25%	52%[$]	-	-	-
A26: The number of parents of young children had a discussion on immunization with their doctors (in the case of voluntary vaccination policy)	53%	33%	48%[$]	-	-	-
A27: The number of institutions incentivized by the government to initiate preventive physical activities at the micro level	58%	50%	56%[$]	-	-	-
A28: The number of patients who avail continuum of care for communicable diseases	67%	67%	67%[$]	-	-	-
A29: The number of individuals reimbursed through their health insurance for preventative physical activities	50%	67%	54%[$]	-	-	-
A30: The number of children fully immunized (in the case of mandatory vaccination policy)	86%	67%	81%	63%	60%	63%[$]
A30N: The share of all insured people listed in a digital vaccination register by population group	-[+]	-[+]	-[+]	60%	20%	52%[$]
A31: The number of children/individuals who have a vaccination card (needed in the case of an emergency outbreak where authority can verify the vaccination status)	86%	50%	78%	68%	20%	58%[$]
A32: The percentage of health promotion programs with communication plans clearly stated	70%	50%	65%[$]	-	-	-
A33: The percentage of health promotion programs with measurable outputs and outcomes and a proper registry system	81%	67%	78%	50%	20%	44%[$]
A34: The number of schools/workplaces/municipalities made physical activities mandatory for children/employees/residents of different age groups	68%	67%	68%[$]	-	-	-
**Indicators on other topics**						
A35: The degree of change of specific health indicators as an outcome measure of the impact of digital tools	69%	100%	74%	67%	100%	79%
A36: The number of hard-copy processes	41%	25%	38%[$]	-	-	-

Indicators on other topics	Round 2			Round 3		
	% Europe (n=22)	% Other (n=6)	% Total (n=28)	% Europe (n=20)	% Other (n=6)	% Total (n=26)
A36N: The number of hard-copy processes in contrast to digital processes in the healthcare system	-[+]	-[+]	-[+]	65%	60%	64%[$]
A37: The percentage of a process run-through accomplished with the tool	86%	0%	75%	65%	0%	55%[$]
A38: The number of skilled professionals in Business Intelligence tools	56%	100%	64%[$]	-	-	-
A39: The number of active Business Intelligence reports	47%	75%	52%[$]	-	-	-
A40: The average rating of a digital health service in a relevant rating portal	-[+]	-[+]	-[+]	75%	100%	79%

*The calculation includes only those experts, who voted on the Likert scale unimportant (1), barely important (2), somewhat important (3), and very important (4). Those that selected the "I can't rate this indicator due to a lack of expertise" were excluded from the consensus calculation.
[+]Indicators or DiPH tools that were first given (as alternative formulations) during the second panel round.
[$]Indicators or DiPH tools with less than 70% votes for "somewhat important" or "very important", which were, therefore, excluded.

Interventions with a share of less than 70% for "somewhat important" or "very important" votes were excluded for the next panel round.
Regarding alternative formulations of indicators, only the phrasing with the highest share of "somewhat important" or "very important" votes were kept.

Supplementary Table 21. Differences by continents: Europe versus other. % of “somewhat important” and “very important ratings”: DiPH Tools

Digital alternatives to traditional health services	Round 2 % Europe (n=24)	Round 2 % Other (n=7)	Round 2 % Total (n=31)	Round 3 % Europe (n=20)	Round 3 % Other (n=6)	Round 3 % Total (n=26)
T01: Electronic referral	86%	78%	90%	84%	100%	88%
T02: Electronic prescription	92%	75%	90%	89%	100%	92%
T03: Electronic patient folder	91%	67%	90%	89%	83%	88%
T04: Electronic registries (e.g., for vaccination)	96%	88%	97%	100%	100%	100%
T05: Electronic medical records	92%	63%	87%	100%	83%	96%
T06: The use of Business Intelligence for monitoring, audit & data exploration	76%	63%	78%	83%	100%	88%
T07: Telelectronic health/telemedicine/telecare	79%	88%	84%	95%	67%	88%
T08: Electronic health record	100%	100%	97%	100%	83%	96%
T09: Digital Health ID (each person can claim 1 unique ID)	88%	88%	90%	85%	100%	88%
T10: Social media chat with health promotion professionals/health care providers	58%	50%	58%[$]	-	-	-
T11: Video consultation	75%	63%	77%	85%	50%	77%
T12: Digital twin' of a patient	33%	13%	33%[$]	-	-	-
T13: Electronic health insurance card	52%	56%	59%[$]	-	-	-
T14: Decision support systems	75%	88%	81%	70%	83%	73%
T15: The use of Business Intelligence for communication and collaboration	61%	33%	61%[$]	-	-	-
T16: Surveillance tools	96%	63%	93%	85%	83%	85%
T17: Digital chronic disease management tools	-[+]	-[+]	-[+]	79%	100%	84%
T18: Digital screening programs	-[+]	-[+]	-[+]	74%	100%	80%
T19: Smart care homes and residences	-[+]	-[+]	-[+]	65%	80%	68%[$]
**Mobile health tools**						
T20: Wearables	75%	75%	77%	65%	67%	65%[$]
T21: Sensors	75%	75%	77%	65%	67%	65%[$]
T22: Mobile tools for data collection	88%	88%	90%	90%	83%	88%
T23: Smartphone & web health or medical apps	91%	67%	90%	85%	83%	85%
T24: Tracking devices (e.g., physical activity, blood pressure)	83%	75%	83%	90%	83%	88%
**Information and education services**						
T25: Dashboards	95%	78%	96%	89%	100%	91%
T26: Data visualization tools	95%	78%	97%	80%	50%	73%
T27: Health information websites by public health authorities/institutions	100%	83%	94%	95%	83%	92%
T28: Healthcare alert systems	87%	88%	90%	74%	80%	75%

Digital alternatives to traditional health services	Round 2 % Europe (n=24)	Round 2 % Other (n=7)	Round 2 % Total (n=31)	Round 3 % Europe (n=20)	Round 3 % Other (n=6)	Round 3 % Total (n=26)
T28N: Digital safety alarms	-+	-+	-+	84%	80%	83%
T29: Serious games for training purposes	39%	56%	47%$	-	-	-
T30: School curricula on digital public health	61%	75%	67%$	-	-	-
T31: Digital health education materials	-+	-+	-+	85%	50%	77%
**Infrastructure service**						
T32: Digital progress hubs	63%	43%	63%$	-	-	-
T33: National health data repositories	88%	88%	90%	78%	83%	79%
T34: Telematikinfrastructure (so that data can be used beyond interfaces on an outpatient and clinical basis)	65%	38%	67%$	-	-	-
T35: Solutions for the transfer of digital measures to regional care that optimize practice	91%	43%	89%	80%	33%	67%$
T36: Risk models and algorithms	79%	88%	84%	76%	40%	68%$
T37: Real-world evidence generation and follow-up agencies	-+	-+	-+	83%	33%	71%

*The calculation includes only those experts, who voted on the Likert scale unimportant (1), barely important (2), somewhat important (3), and very important (4). Those that selected the "I can't rate this indicator due to a lack of expertise" were excluded from the consensus calculation.
+Indicators or DiPH tools that were first given (as alternative formulations) during the second panel round.
$Indicators or DiPH tools with less than 70% votes for "somewhat important" or "very important", which were, therefore, excluded.

Interventions with a share of less than 70% for "somewhat important" or "very important" votes were excluded for the next panel round.
Regarding alternative formulations of indicators, only the phrasing with the highest share of "somewhat important" or "very important" votes were kept.

Supplementary File 3: Balanced panel and sensitivity analysis

For our Delphi study, we encouraged every registered participant who met our inclusion criteria to participate in each round, no matter if they had contributed to the previous round or not. As this approach might have led to an attribution bias, we conducted a balanced panel analysis for assessing for significant differences in voting behavior of somewhat important" and "very important" choices among the 30 experts who participated in all rounds and the total group of experts for each round. A conflict was defined as either the balanced panel or the total participating cohort with at least 70% agreement on keeping an indicator (70% of the participants choosing "somewhat important" or "very important") while the other group had a lower overall rating. The results of this analysis are displayed below.

3/30 Information-communication-technology (*ICT)* indicators, 2/34 *Legal* indicators, 2/41 *Social* indicators, 5/38 *Application* indicators, and 1/32 *Digital Public Health (DiPH) tools* caused conflicts during the second round. During the third round, 4/33 *ICT* indicators, 4/38 *Legal* indicators, 1/35 *Social* indicators, 2/33 *Application* indicators, and 4/30 *DiPH tools* had differences between overall sub-group agreement share. For more details, see Supplementary Tables 23 to 27.

Based on these findings, we conducted a sensitivity analysis to assess if the overall share of "somewhat important" or "very important" ratings for each of the sub-domain was significantly biased by decisions from the balanced panel compared to the total study cohort per Delphi round. After testing for normal distribution through the Kolmogorov-Smirnov test, we conducted a two-sided Gaussian test with alpha 5% (normal distribution was given for all four dimensions and DiPH tools). The p-values and the sub-group sizes per domain are displayed in Supplementary Table 22. We did not identify any significant differences between groups with a 95% confidence interval.

Supplementary Table 22. Significance in differences for ratings among sub-groups

Domain	Round 2	Round 3
ICT	p = 0,97<br>(n = 14 vs 20)	p = 0,81<br>(n = 13 vs 15)
Legal	p = 0,67<br>(n = 15 vs 22)	p = 0,53<br>(n = 13 vs 17)
Social	p = 0,73<br>(n = 15 vs 19)	p = 0,92<br>(n = 11 vs 16)
Application	p = 0,64<br>(n = 21 vs 28)	p = 0,79<br>(n = 17 vs 26)
Tool	p = 0,70<br>(n = 22 vs 31)	p = 0,83<br>(n = 17 vs 26)

* Statistically significant difference between groups with 95% CI

Supplementary Table 23. Balanced panel analysis with % of "somewhat important" and "very important" ratings: ICT

Indicators on expenses, investment and workforce	Round 2		Round 3	
	% Balanced Panel (n=14)	% Total (n=20)	% Balanced Panel (n=13)	% Total (n=15)
I01: The ratio between private health expenditure and household disposable income health expenditure	46%	56%$	-	-
I02: The average prices for fixed broadband Internet connections per month	86%	75%	77%	73%
I03: The average prices for pre-paid contracts for mobile phones per month	71%	70%	62%	60%$
I04: The annual telecommunications revenue (% of gross domestic product)	43%	32%$	-	-
I05: The annual telecommunications investment (% of revenue)	71%	58%$	-	-
I06: The percentage of health expenditure in gross domestic product (public and private)	79%	74%	58%	64%$
I07: The annual spending by the government on the information-communication-infrastructure	79%	80%	92%	87%
I08: The level of feedback and communication with various publics	50%	47%$	-	-
I09: The level of education	85%	89%	69%	67%$
I09N: The level of training in the use and billing of digital public health services by health care professionals	-+	-+	85%	87%
I10: The number of feedbacks from patients	79%	95%	75%	64%$
I11: The number of skilled professionals in data science	92%	95%	92%	87%
I12: The proportion of the total business sector workforce involved in the information-telecommunication-technology sector	64%	58%$	-	-
I13: The number of participants in training programs for using digital public health tools by groups (e.g., healthcare workers, patients, or family caregivers)	79%	85%	58%	57%$
I13N1: The average number of participants in digital public health tool training programs in a year by different target groups	-+	-+	92%	93%
I13N2: The average number of digital public health tool training provided to different target groups	-+	-+	83%	79%
**Indicators on the availability of the Internet and Internet-enabled devices such as PCs, phones & tablets**				
I14: The share of households/hospitals/physician offices connected to the Internet (by bandwidth)	93%	95%	92%	93%
I15: The personal computers per 100 people	64%	58%$	-	-
I16: The secure Internet servers (per 1 million people)	69%	61%$	-	-
I17: The share of households, hospitals & physician offices with a computer	93%	95%	75%	71%
I17N: The share of specific locations that are equipped with at least one Internet-connected device by location (e.g., households, hospitals or outpatient physician's offices)	-+	-+	100%	100%
I18: The number of Internet-connected devices per 100 people by the device (e.g., computers, smartphones, tablets, or notebooks)	-+	-+	69%	73%
I19: The percentage of the overall population with access to the Internet by speed	-+	-+	69%	93%
I20: The percentage of the overall population with access to computers	-+	-+	75%	73%
I21: The percentage of the population covered by at least a 3G mobile network	79%	79%	85%	87%
I22: The telephone lines per 100 people	43%	32%$	-	-
I23: The percentage of the overall population with access to smartphones	-+	-+	100%	100%

Indicators on interoperability and infrastructure	Round 2		Round 3	
	% Balanced Panel (n=14)	% Total (n=20)	% Balanced Panel (n=13)	% Total (n=15)
I24: The share or paperless hospitals of all hospitals, when delivering and managing patient care	100%	89%	83%	79%
I24N: The percentage of all hospitals that use electronic documentation systems for patient care	-+	-+	100%	100%
I25: The availability of health information exchange platforms	100%	100%	92%	93%
I26: The existence of a decentralized infrastructure that leaves the data where they originated & accessible based on consent and ID approval	85%	89%	75%	71%
I26N1: The existence of a decentralized data infrastructure	-+	-+	64%	62%$
I26N2: The existence of the possibility to access personal health data based on consent and ID approval	-+	-+	100%	100%
I27: The degree of existing telematic infrastructure	83%	83%	92%	93%
I28: The degree of interoperability of health systems	92%	95%	100%	100%
I29: The degree of compliance of specific interventions with the data exchange and interoperability standards	92%	95%	91%	92%
I30: The existence of interoperable data end-to-end encryption	75%	82%	82%	85%
I31: The number of integrated digital systems in the healthcare system	-+	-+	71%	72%
**Indicators on other topics**				
I32: The percentage of operators with a waiting time less than the guaranteed maximum response time	69%	76%	67%	64%$
I33: The number of adverse events	64%	73%	73%	69%$
I33N: The number of technical malfunctions per digital public health tool reported per year	-+	-+	83%	86%
I34: The percentage of users with first hospital specialty appointments performed within the reference time	55%	60%$	-	-
I35: The degree of internationalization	50%	53%$	-	-

*The calculation includes only those experts, who voted on the Likert scale unimportant (1), barely important (2), somewhat important (3), and very important (4). Those that selected the “I can’t rate this indicator due to a lack of expertise” were excluded from the consensus calculation.
+Indicators or DiPH tools that were first given (as alternative formulations) during the second panel round.
$Indicators or DiPH tools with less than 70% votes for “somewhat important” or “very important”, which were, therefore, excluded.

Interventions with a share of less than 70% for “somewhat important” or “very important” votes were excluded for the next panel round.
Regarding alternative formulations of indicators, only the phrasing with the highest share of “somewhat important” or “very important” votes were kept.

Supplementary Table 24. Balanced panel analysis with % of "somewhat important" and "very important" ratings: Legal

	Round 2		Round 3	
**Indicators on health data (access, exchange, security)**	**% Balanced Panel (n=15)**	**% Total (n=22)**	**% Balanced Panel (n=13)**	**% Total (n=17)**
L01: The percentage of user consent to health data	77%	85%	73%	75%
L02: The coverage of international standards in stored data	75%	78%	83%	76%
L03: The existence of a legal framework for exchanging health data digitally between different stakeholders	100%	100%	100%	100%
L04: The degree of political support in data transfer & exchange	79%	81%	75%	76%
L05: The existence of a legal framework for the secondary use of health data	93%	90%	92%	94%
L06: The existence of regulations for access to health data through electronic health records	100%	95%	100%	100%
L07: The existence of legislation regulating the interaction between the digital data of a patient's health and the data of bioinformatics / genetic information of the biomaterials of this patient	-[+]	-[+]	67%	79%
L08: The level of encryption of personal and health data	92%	90%	75%	82%
L09: The ratio of encrypted records to the total number of records	75%	74%	73%	69%[$]
L10: The existence of regulations that the digital data collected via electronic health applications are only stored on one's own electronic health card	82%	76%	70%	67%[$]
L11: The coverage of metadata labels in sensitive data	82%	88%	80%	79%
L12: The number of critical failures in security points	92%	95%	82%	88%
L13: The number of weaknesses in security perimeters	83%	89%	80%	86%
L14: The level of data security	100%	100%	80%	87%
**Indicators on digital assets**				
L15: The existence of a policy or legislative reform to allow live-time digital asset	67%	81%	64%	62%[$]
L16: The existence of policy/legislative reform to allow access to all digital assets that would fit into the World Health Organizations' definition of health and may be considered non-health assets (education, Social system, work, income, etc.) traditionally	64%	72%	55%	50%[$]
L17: The existence of a policy or legislative reform to allow access to digital assets	82%	82%	82%	75%
**Indicators on the digital public health strategy**				
L18: The existence of political briefs/policies that mention digital health	64%	67%[$]	-	-
L19: The existence of a political strategy to digitalize the healthcare system	100%	95%	92%	88%
L20: The existence of a digital public health strategy within the governmental health strategy	92%	90%	92%	88%
L21: The existence of guidelines for planning & implementing digital public health tools	86%	81%	92%	94%
L22: The level of harmonization of legal regulations in electronic health	100%	95%	67%	65%[$]
L23: The existence of legal supervision of the implementation of national digital public health programs	79%	81%	92%	88%
L24: The existence of a department for digital health in the health ministry	71%	81%	82%	81%
L25: The existence of regulation on the function of electronic health products (e.g., Medical Devices Act)	77%	80%	91%	94%

Indicators on the digital public health strategy	Round 2		Round 3	
	% Balanced Panel (n=15)	% Total (n=22)	% Balanced Panel (n=13)	% Total (n=17)
L26: The existence of a legal right for citizens to be provided with digital health services	-+	-+	70%	73%
L27: The existence of a digital public health policy engaged with the protection of fundamental rights of vulnerable groups (e.g., children, adolescents, mentally disabled people)	-+	-+	83%	76%
L28: The existence of a policy to promote innovation and the development of digital tools in the public health system	-+	-+	92%	82%
**Indicators on finances and reimbursement**				
L29: The existence of a public funding scheme for digital public health interventions on a regional and national level	77%	70%	83%	82%
L30: The existence of investment and reimbursement possibilities from the government	75%	79%	92%	88%
L31: The annual spending by the government as support for the implementation of digital technologies in healthcare	85%	85%	73%	75%
L32: The number of digital applications remunerated	45%	44%$	-	-
L33: The existence of financial incentives for health professionals to participate in offering digital public health services	85%	75%	83%	82%
**Indicators on other topics**				
L34: The availability of legal tools, such as contracts templates	50%	58%$	-	-
L35: The rate of Direct Public Offering (DPO) approval and other activities	14%	18%$	-	-
L36: The existence of a law/regulation on prevention on the regional/national level	57%	62%$	-	-
L37: The efficiency of the alarm system at all levels of healthcare	33%	56%$	-	-
L38: The number of civic bodies that have the autonomy to organize the health care services at least at the primary and secondary levels of care (decentralization component)	25%	32%$	-	-
L39: The existence of an action plan to prevent discrimination based on race, gender, sexual orientation & age in digital health services	-+	-+	73%	63%$
L40: The existence of a statutory provision that places restrictions and quarantine periods on public health leadership professionals for future private digital health activities after they have ceased their previous public service activities	-+	-+	40%	40%$
L41: The existence of legal liability for public health managers for digital health contracts that harm patients or the public interest	-+	-+	80%	73%
L42: The existence of policy standards for transparency and the protection of fundamental rights in using artificial intelligence in digital public health	-+	-+	92%	94%
L43: The level of transparency and accessibility of digital health contracts to public oversight and law enforcement agencies for anti-corruption purposes	-+	-+	83%	71%
L44: The effectiveness of informed patient consent for using personal health data in adult patients	-+	-+	92%	76%
L45: The existence of unique procedures to protect children, adolescents and mentally ill patients who are unable to give their consent	-+	-+	92%	76%

Supplementary Table 25. Balanced panel analysis with % of "somewhat important" and "very important" ratings: Social

Indicators on potential and actual users	Round 2 % Balanced Panel (n=15)	Round 2 % Total (n=19)	Round 3 % Balanced Panel (n=11)	Round 3 % Total (n=16)
S01: The number of people that are willing to use a digital public health tool or participate in a digital public health intervention	93%	95%	92%	88%
S02: The number of potential users of the same digital public health tool	80%	79%	91%	87%
S03: The number of potential users of a digital public health tool who have adequate access to the web	86%	89%	83%	81%
S04: The share of the eligible population who have used at least one digital public health intervention for routine care and health promotion in the previous year	80%	74%	83%	88%
S05: The share of the eligible population who have signed up (i.e., have an account) for at least one digital public health intervention focused on routine care and health promotion	67%	68%[$]	-	-
S06: The number of patients using apps to interact with local services	87%	84%	83%	88%
S07: The number of digital contacts	69%	65%[$]	-	-
S08: The adherence of users to the intervention (in percentage)	77%	71%	73%	73%
S09: The ratio of electronic health records to the total number of user records	77%	81%	73%	73%
S10: The number of patients enrolled after communication campaigns	67%	63%[$]	-	-
S11: The number of patients enrolled in app use	73%	74%	33%	38%[$]
S11N1: The number of patients enrolled in the use of specific health apps	-[+]	-[+]	75%	75%
S11N2: The share of the population that uses any health or medical app by reason (e.g., health promotion, wellness, tracing)	-[+]	-[+]	75%	81%
S12: The number of digital public health professionals	-[+]	-[+]	71%	76%
S13: The number of patients interacting with apps	80%	68%[$]	-	-
S14: The number of digital platforms used and views reach and interaction	60%	63%[$]	-	-
S15: The number of posts placed on digital platforms	33%	32%[$]	-	-
S16: The utilization rate of specific groups per digital public health intervention (by health insurance claims data coding)	86%	88%	64%	57%[$]
**Indicators on awareness, trust & motivation**				
S17: The perceived usefulness of specific digital public health tools by groups on a Likert scale	79%	76%	80%	79%
S18: The share of the population that trusts digital health services	87%	89%	92%	88%
S19: The motivation to access electronic health services by groups of people	71%	78%	82%	73%
S20: The awareness of health professionals about the value of data and the possibility of their use by information-telecommunication-technology	87%	89%	92%	88%
S21: The awareness of groups of people that the intervention exists (by target groups)	87%	79%	100%	94%
S22: The level of trust in the government by different groups of the population	-[+]	-[+]	67%	63%[$]
S23: The satisfaction rate	71%	74%	55%	53%[$]

Indicators on awareness, trust & motivation	Round 2 % Balanced Panel (n=15)	Round 2 % Total (n=19)	Round 3 % Balanced Panel (n=11)	Round 3 % Total (n=16)
S23N1: The self-reported satisfaction rate with digital public health intervention by user group	-[+]	-[+]	75%	75%
S23N2: The share of the population that is more satisfied with using the digital public health intervention compared to standard care	-[+]	-[+]	83%	81%
**Indicators on literacy**				
S24: The average level of digital literacy by different target groups on a Likert Scale	93%	89%	100%	93%
S25: The average level of digital health literacy by different target groups on a Likert Scale	100%	94%	91%	87%
S26: The average level of health literacy by different target groups on a Likert Scale	77%	76%	91%	87%
S27: The average level of information-telecommunication-technology skills by groups of people	86%	89%	92%	88%
**Indicators on use of smart- and cellular phones**				
S28: The share of the population that uses a smartphone by different target groups	93%	95%	83%	81%
S29: The share of the population that owns a smartphone by different target groups	80%	84%	75%	75%
S30: The smartphone penetration rate versus the use of mobile devices for routine care and health promotion in the population	100%	95%	92%	87%
S31: The mobile cellular subscriptions per 100 people	53%	44%[$]	-	-
S32: The proportion of healthcare users using smartphones and other digital devices	80%	83%	75%	81%
**Indicators on use of the Internet**				
S33: The active mobile-broadband subscriptions per 100 inhabitants	79%	75%	91%	92%
S34: The fixed broadband Internet subscriptions per 100 inhabitants	67%	74%	85%	80%
S35: The percentage of Internet users in the country	93%	95%	85%	87%
S36: The share of the population that uses the Internet for gathering health information by different target groups	87%	89%	75%	81%
S37: The number of searches for specific digital public health interventions measured on Google Trends	-[+]	-[+]	75%	75%
**Indicators on other topics**				
S38: The number of various health promotion campaigns led by students/youth	47%	42%[$]	-	-
S39: The number of professional bodies consulted for patient-provider interactions regarding the service of routine care	50%	44%[$]	-	-
S40: The number of patients contacted to obtain feedback on routine care for chronic diseases	57%	61%[$]	-	-
S41: The degree of impact of communication campaigns	50%	47%[$]	-	-
S42: The number of disease-specific patient groups consulted for discussing care and treatment modalities in chronic diseases	79%	72%	56%	62%[$]
S43: The number of prospective parents reached out for counseling on child immunization	50%	44%[$]	-	-
S44: The number of institutions (schools/workplaces and others) that develop mechanisms through the consultative process (with children/employees) for promoting/adopting the government-sponsored/facilitated preventive physical activities	69%	59%[$]	-	-

Supplementary Table 26. Balanced panel analysis with % of "somewhat important" and "very important" ratings: Application

	Round 2		Round 3	
**Indicators on access to the service and information**	**% Balanced Panel (n=22)**	**% Total (n=31)**	**% Balanced Panel (n=17)**	**% Total (n=26)**
A01: The number of patients reached through telemedicine	65%	67%[$]	-	-
A02: The availability of digital information on public health interventions as national prevention programs	85%	85%	78%	77%
A02N1: The share of available digital information on public health interventions	-[+]	-[+]	67%	69%[$]
A02N2: The availability of reliable information on specific digital public health services	-[+]	-[+]	94%	92%
A03: The number of individuals reached through digital public health system (via mobile messaging) for health promotion information/communication	70%	67%[$]	-	-
A04: The percentage of the overall population with access	89%	92%	71%	68%[$]
A04N: The percentage of the overall population with access to the digital public health tool	-[+]	-[+]	100%	100%
A05: The availability of reliable health information in a digital format	85%	85%	76%	84%
A06: The proportion of persons who cannot access their digital data and the provision of alternative access	-[+]	-[+]	89%	88%
**Indicators on secondary use of health data**				
A07: The share of patient health data used for monitoring or evaluating the healthcare system	89%	85%	71%	72%
A07N1: The share of patient health data used for evaluating healthcare services	-[+]	-[+]	72%	73%
A07N2: The share of population health data used for public health monitoring	-[+]	-[+]	78%	73%
A08: The percentage run-throughs completed (re)use of data	64%	58%[$]	-	-
A09: The degree of data interoperability	100%	96%	71%	75%
A09N: The degree of technical/syntactic/semantic interoperability	-[+]	-[+]	71%	91%
A10: The degree of data linkage	89%	88%	94%	72%
A10N: The availability of a unique identifier to link health data for a person between different digital public health tools/platforms	-[+]	-[+]	72%	96%
A11: The degree of data accessibility	100%	92%	100%	96%
**Indicators on service implementation**				
A12: The degree to which an intervention is established (e.g., local pilot, communal, regional, national)	88%	87%	94%	77%
A12N: The level to which an intervention is established (e.g., local pilot, communal, regional, national)	-[+]	-[+]	89%	61%[$]
A13: The degree to which the intervention complements existing workflows or duplicates them	76%	75%	60%	50%[$]
A13N: The extent of redundancy in workflows created by introducing digital public health tools/interventions to complement existing workflows	-[+]	-[+]	47%	75%
A14: The percentage of health promotion programs integrated into national health system digital platforms	65%	67%[$]	-	-
A15: The proportion of (specific) public health services delivered via digital technologies (may be disaggregated by technology type)	74%	65%[$]	-	-
A16: The proportion of digital public health interventions considering health equity in their planning, implementation & evaluation	85%	85%	88%	92%

Indicators on service implementation	Round 2 % Balanced Panel (n=22)	Round 2 % Total (n=31)	Round 3 % Balanced Panel (n=17)	Round 3 % Total (n=26)
A17: The proportion of public health services implementing at least one digital technology intervention	44%	48%$	-	-
A18: The number of regulated digital health services included in routine care	80%	78%	78%	73%
A19: The total number of records	55%	61%$	-	-
**Indicators on traditional health services and prevention**				
A20: The number of patients recommended by their treating physician to adopt regular physical activities (such as joining fitness club/swimming)	60%	59%$	-	-
A21: The number of patients suffering from non-communicable chronic diseases	80%	81%	50%	42%$
A22: The number of patients suffering from communicable diseases (separately: prevalence & incidence)	85%	81%	50%	42%$
A23: The number of patients suffering from communicable diseases and in need of a continuum of care	85%	81%	47%	38%$
A24: The number of patients reached through community-based mental health services	75%	74%	53%	48%$
A25: The number of individuals/children excluded from the incentivization scheme of preventive physical activities	59%	52%$	-	-
A26: The number of parents of young children had a discussion on immunization with their doctors (in the case of voluntary vaccination policy)	44%	48%$	-	-
A27: The number of institutions incentivized by the government to initiate preventive physical activities at the micro level	56%	56%$	-	-
A28: The number of patients who avail continuum of care for communicable diseases	67%	67%$	-	-
A29: The number of individuals reimbursed through their health insurance for preventative physical activities	63%	54%$	-	-
A30: The number of children fully immunized (in the case of mandatory vaccination policy)	85%	81%	65%	63%$
A30N: The share of all insured people listed in a digital vaccination register by population group	-+	-+	47%	52%$
A31: The number of children/individuals who have a vaccination card (needed in the case of an emergency outbreak where authority can verify the vaccination status)	75%	78%	63%	58%$
A32: The percentage of health promotion programs with communication plans clearly stated	68%	65%$	-	-
A33: The percentage of health promotion programs with measurable outputs and outcomes and a proper registry system	75%	78%	53%	44%$
A34: The number of schools/workplaces/municipalities made physical activities mandatory for children/employees/residents of different age groups	72%	68%$	-	-
**Indicators on other topics**				
A35: The degree of change of specific health indicators as an outcome measure of the impact of digital tools	80%	74%	80%	79%
A36: The number of hard-copy processes	38%	38%$	-	-
A36N: The number of hard-copy processes in contrast to digital processes in the healthcare system	-+	-+	71%	64%$
A37: The percentage of a process run-through accomplished with the tool	64%	75%	53%	55%$
A38: The number of skilled professionals in Business Intelligence tools	73%	64%$	-	-
A39: The number of active Business Intelligence reports	57%	52%$	-	-
A40: The average rating of a digital health service in a relevant rating portal	-+	-+	75%	79%

Supplementary Table 27. Balanced panel analysis with % of "somewhat important" and "very important" ratings: DiPH Tools

Digital alternatives to traditional health services	Round 2		Round 3	
	% Balanced Panel (n=22)	% Total (n=31)	% Balanced Panel (n=17)	% Total (n=26)
T01: Electronic referral	85%	90%	83%	88%
T02: Electronic prescription	86%	90%	89%	92%
T03: Electronic patient folder	90%	90%	83%	88%
T04: Electronic registries (e.g., for vaccination)	95%	97%	100%	100%
T05: Electronic medical records	91%	87%	100%	96%
T06: The use of Business Intelligence for monitoring, audit & data exploration	84%	78%	88%	88%
T07: Telelectronic health/telemedicine/telecare	86%	84%	83%	88%
T08: Electronic health record	100%	97%	100%	96%
T09: Digital Health ID (each person can claim 1 unique ID)	95%	90%	89%	88%
T10: Social media chat with health promotion professionals/health care providers	55%	58%[$]	-	-
T11: Video consultation	76%	77%	67%	77%
T12: Digital twin' of a patient	31%	33%[$]	-	-
T13: Electronic health insurance card	57%	59%[$]	-	-
T14: Decision support systems	77%	81%	67%	73%
T15: The use of Business Intelligence for communication and collaboration	58%	61%[$]	-	-
T16: Surveillance tools	95%	93%	83%	85%
T17: Digital chronic disease management tools	-[+]	-[+]	76%	84%
T18: Digital screening programs	-[+]	-[+]	71%	80%
T19: Smart care homes and residences	-[+]	-[+]	61%	68%[$]
**Mobile health tools**				
T20: Wearables	91%	77%	61%	65%[$]
T21: Sensors	86%	77%	61%	65%[$]
T22: Mobile tools for data collection	100%	90%	83%	88%
T23: Smartphone & web health or medical apps	90%	90%	78%	85%
T24: Tracking devices (e.g., physical activity, blood pressure)	86%	83%	83%	88%
**Information and education services**				
T25: Dashboards	95%	96%	94%	91%
T26: Data visualization tools	95%	97%	83%	73%
T27: Health information websites by public health authorities/institutions	91%	94%	94%	92%

Information and education services	Round 2 % Balanced Panel (n=22)	Round 2 % Total (n=31)	Round 3 % Balanced Panel (n=17)	Round 3 % Total (n=26)
T28: Healthcare alert systems	91%	90%	83%	75%
T28N: Digital safety alarms	-+	-+	94%	83%
T29: Serious games for training purposes	48%	47%$	-	-
T30: School curricula on digital public health	68%	67%$	-	-
T31: Digital health education materials	-+	-+	83%	77%
**Infrastructure service**				
T32: Digital progress hubs	95%	63%$	-	-
T33: National health data repositories	68%	90%	82%	79%
T34: Telematikinfrastructure (so that data can be used beyond interfaces on an outpatient and clinical basis)	89%	67%$	-	-
T35: Solutions for the transfer of digital measures to regional care that optimize practice	86%	89%	87%	67%$
T36: Risk models and algorithms	95%	84%	79%	68%$
T37: Real-world evidence generation and follow-up agencies	-+	-+	88%	71%

*The calculation includes only those experts, who voted on the Likert scale unimportant (1), barely important (2), somewhat important (3), and very important (4). Those that selected the “I can’t rate this indicator due to a lack of expertise” were excluded from the consensus calculation.
+Indicators or DiPH tools that were first given (as alternative formulations) during the second panel round.
$Indicators or DiPH tools with less than 70% votes for “somewhat important” or “very important”, which were, therefore, excluded.

Interventions with a share of less than 70% for “somewhat important” or “very important” votes were excluded for the next panel round.
Regarding alternative formulations of indicators, only the phrasing with the highest share of “somewhat important” or “very important” votes were kept.
