# Supplementary material for: International perspectives on measuring national digital public health system maturity through a multidisciplinary Delphi study
Source: NPJ Digit Med. 2024 Apr 12;7:92. doi: 10.1038/s41746-024-01078-9 (PMC11014962; doi:10.1038/s41746-024-01078-9)
Supplement: Supplementary file 2 — Reporting Summary [file 41746_2024_1078_MOESM2_ESM.pdf]

nature portfolio

Corresponding author(s): Laura Maaß

Last updated by author(s): 28.02.2024

Reporting Summary

Nature Portfolio wishes to improve the reproducibility of the work that we publish. This form provides structure for consistency and transparency in reporting. For further information on Nature Portfolio policies, see our Editorial Policies and the Editorial Policy Checklist.

Statistics

For all statistical analyses, confirm that the following items are present in the figure legend, table legend, main text, or Methods section.

n/a	Confirmed	
☐	☒	The exact sample size (*n*) for each experimental group/condition, given as a discrete number and unit of measurement
☐	☒	A statement on whether measurements were taken from distinct samples or whether the same sample was measured repeatedly
☐	☒	The statistical test(s) used AND whether they are one- or two-sided<br>*Only common tests should be described solely by name; describe more complex techniques in the Methods section.*
☒	☐	A description of all covariates tested
☐	☒	A description of any assumptions or corrections, such as tests of normality and adjustment for multiple comparisons
☐	☒	A full description of the statistical parameters including central tendency (e.g. means) or other basic estimates (e.g. regression coefficient) AND variation (e.g. standard deviation) or associated estimates of uncertainty (e.g. confidence intervals)
☒	☐	For null hypothesis testing, the test statistic (e.g. F, t, r) with confidence intervals, effect sizes, degrees of freedom and P value noted<br>*Give P values as exact values whenever suitable.*
☒	☐	For Bayesian analysis, information on the choice of priors and Markov chain Monte Carlo settings
☒	☐	For hierarchical and complex designs, identification of the appropriate level for tests and full reporting of outcomes
☒	☐	Estimates of effect sizes (e.g. Cohen's d, Pearson's r), indicating how they were calculated

Our web collection on statistics for biologists contains articles on many of the points above.

Software and code

Policy information about availability of computer code

Data collection	Online survey conducted through commercial tool QuestionPro (GDPR conformative online survey platform)
Data analysis	Microsoft Excel 2019

For manuscripts utilizing custom algorithms or software that are central to the research but not yet described in published literature, software must be made available to editors and reviewers. We strongly encourage code deposition in a community repository (e.g. GitHub). See the Nature Portfolio guidelines for submitting code & software for further information.

Data

Policy information about availability of data

All manuscripts must include a data availability statement. This statement should provide the following information, where applicable:

- Accession codes, unique identifiers, or web links for publicly available datasets
- A description of any restrictions on data availability
- For clinical datasets or third party data, please ensure that the statement adheres to our policy

The anonymized datasets generated during and/or analyzed during the current study are available from the corresponding author on reasonable request.

Human research participants

Policy information about studies involving human research participants and Sex and Gender in Research.

Reporting on sex and gender	During the online pre-survey, participants were asked "What gender do you identify with?". They were able to select one of the following answers: female, male, transgender, non-binary, I prefer not to answer, other. We did not collect data on sex. We did not conduct sub-group analysis for different genders among the study population.
Population characteristics	We collected the following information of participants: Highest academic qualification, the professional background, the sector they currently work in, the years of experience in their professional field in general, the years of experience in digital (public) health, their age, gender, their country of residence, and to which of the four perspectives they'd like to contribute
Recruitment	Participants were recruited through different channels: First, experts in the network of the authors were approached and invited for participation Further, experts were identified through their association with research institutes/teaching obligations at Universities related to the topic. Also, people listed as editors for internationally published and peer-reviewed digital health journals were approached via e-Mail. The invitaltion e-Mail included a brief description of the study, the invitation to share the invitation (snowball method), and the link to a pre-survey where we checked if the inclusion criteria were met. The invitation for participation was further distributed through newsletters from relevant associations/institutes and via social media (Twitter and LinkedIn).<br>As the authors are based in Germany and have a background in Public Health, German experts and experts in Public Health were overrepresented in this study (due to personal network) compared to experts from other countries or with a different background. We conducted a sub-group and sensitivity analysis to see if these groups decided significantly differently than other participants.
Ethics oversight	N/A

Note that full information on the approval of the study protocol must also be provided in the manuscript.

Field-specific reporting

Please select the one below that is the best fit for your research. If you are not sure, read the appropriate sections before making your selection.

☐ Life sciences ☒ Behavioural & social sciences ☐ Ecological, evolutionary & environmental sciences

For a reference copy of the document with all sections, see nature.com/documents/nr-reporting-summary-flat.pdf

Behavioural & social sciences study design

All studies must disclose on these points even when the disclosure is negative.

Study description	We conducted a qualitative Delphi Study (with three rounds) to collect and rate quality indicators to measure the maturity of digital public health (DiPH) systems on a national scale. Participants were invited to name and rank indicators from 4 perspectives: The information-communication-technological requirements, the needed legal framework, the collective social willingness and capability to use DiPH tools in routine care and health promotion, and the level of implementation of such tools in the national healthcare system. We did not provide the experts with any indicators to reduce risk of bias. Instead, the experts were asked to name indicators on their own and re-define indicators proposed by other participants if needed during following panel rounds.
Research sample	Participants needed to have at least a Bachelor's degree in Computer Science, Epidemiology, Ethics, Law, Medical Informatics, Medicine, Politics, Public Health, Sociology, or a comparable discipline. They needed to have at least three years of experience in their field and at least one year experience in designing, implementing, regulating, or evaluating digital (public) health tools. Further, they needed to be at least 21 years old, had to read, write, and understand English, and needed to have access to the internet during the study period to participate in the surveys.
Sampling strategy	Participants were recruited through different channels: First, experts in the network of the authors were approached and invited for participation. Further, experts were identified through their association with research institutes/teaching obligations at Universities related to the topic. Also, people listed as editors for internationally published and peer-reviewed digital health journals were approached via e-Mail. The invitation for participation was further distributed through newsletter from relevant associations/ institutes and via social medica (Twitter and digital health groups on LinkedIn). The E-Mail included a brief description of the study, the invitation to share the invitation (snowball method), and the link to a pre-survey where we checked if the inclusion criteria were met. As scientific literature suggested that 10-15 participants are sufficient for a Delphi Study and we caclulated with a response rate of 50%, we opted for 30 participating experts per perspective (as defined above). Recruitment was, therefore, continued until each sub-domain listed at least 30 participating experts as indicated in the pre-survey.
Data collection	All surveys were conducted through the commercial and GDPR-conformative tool QuestionPro. Participants created an alias during the pre-survey, which they used to log in each of the three Delphi rounds. This allowed the authors to link the responses to the same person. The participant-related information from the pre-survey was collected in a locally stored and password secured Microsoft

	Excel 2019 file, while all responsed from the three Delphi panels were collected in another Microsoft Excel 2019 file (also password secured and locally stored) to blind the authors during data analysis.
Timing	The first panel happened from 16th May to 6th June 2022, the second panel was from 13th to 27th July 2022, and the third survey panel was from 8th July to 15th September. Between each round, the responses were analyzed and prepared for the next panel survey. Additionally, each panel questionnaire and design was tested among colleagues who did not belong to the research team.
Data exclusions	During the first survey, experts named 489 indicators and digital public health tools. Of these, 157 were excluded as they used the same terminology as other indicators. Another 147 indicators were excluded as they displayed constructs but were not phrased as measurable indicators. Finally, the results from the first panel were summarized into 136 individual indicators and 32 DiPH-tools. These were reduced by the participants to 96 indicators and 25 DiPH-tools after the third Delphi survey. Indicators and tools were excluded if less than 70% of the participants ranked them as 3-4 on a 4-point Likert scale ("somewhat important" or "very important").
Non-participation	346 experts were contacted directly by the authors.It is unclear, how many experts were reached through newsletters or social media. In total, 87 experts signed up for the study n the pre-survey, however, five did not meet the inclusion criteria and were excluded. Of the final 82 experts, 40 participated in the first Delphi round, 47 participated in the second panel, and 41 in the third panel. 32 experts tool part in all three rounds and 54 contributed to at least one panel round. For each round, one invitation and two reminder E-Mails were send to all non-participating experts to decrease the non-participation rate.
Randomization	N/A

Reporting for specific materials, systems and methods

We require information from authors about some types of materials, experimental systems and methods used in many studies. Here, indicate whether each material, system or method listed is relevant to your study. If you are not sure if a list item applies to your research, read the appropriate section before selecting a response.

Materials & experimental systems

n/a	Involved in the study
☒	☐ Antibodies
☒	☐ Eukaryotic cell lines
☒	☐ Palaeontology and archaeology
☒	☐ Animals and other organisms
☒	☐ Clinical data
☒	☐ Dual use research of concern

Methods

n/a	Involved in the study
☒	☐ ChIP-seq
☒	☐ Flow cytometry
☒	☐ MRI-based neuroimaging
